# TRPML1 inhibitor ML-SI1 suppresses prostate cancer through dual regulation of androgen receptor and polyamine metabolism

**DOI:** 10.3389/fmed.2026.1959805

**Published:** 2026-09-28

**Authors:** Jiongzheng Li, Jipeng Ding, Hongfei Wei, Rongrong Ding, Jintong Zhu, Yufei Yan, Yiming Yang, Jia Zhao, Jing Li

**Affiliations:** 1 Department of Pharmacology, College of Basic Medical Sciences, The Medical Basic Research Innovation Center of Airway Disease in North China, Key Laboratory of Pathobiology, Ministry of Education, Jilin University, Changchun, China; 2 Department of Cell Biology and Medical Genetics, College of Basic Medical Sciences, Jilin University, Changchun, China; 3 Department of Histology and Embryology, College of Basic Medical Sciences, Jilin University, Changchun, China

**Keywords:** androgen receptor, cell migration, ML-SI1, polyamine metabolism, prostate cancer, spermine, TRPML1

## Abstract

**Background:**

Prostate cancer (PCa) is the second leading cause of cancer-related death in men worldwide, with metastasis and drug resistance representing major drivers of mortality. Transient receptor potential mucolipin 1 (TRPML1), a member of the transient receptor potential channel superfamily, is associated with several malignancies; however, its role in PCa remains unclear.

**Methods:**

TRPML1 expression in PCa is analyzed using public datasets from The Cancer Genome Atlas, Human Protein Atlas, and Cancer Cell Line Encyclopedia. Functional studies are performed in prostate cancer cell lines, particularly LNCaP and 22Rv1, using the TRPML1 inhibitor ML-SI1 and TRPML1-knockout models. Cell proliferation, cell cycle distribution, migration, and invasion are assessed using colorimetric cell viability assays, flow cytometry, and Transwell assays. Transcriptomic analysis, quantitative real-time PCR, western blotting, and spermine measurement are performed to investigate the underlying mechanisms.

**Results:**

TRPML1 expression is elevated in PCa tissues compared to adjacent normal tissues. In LNCaP cells, ML-SI1 reduces cell viability and migration and induces G0/G1-phase arrest, whereas in 22Rv1 cells, it exerts limited effects on cell viability and cell-cycle distribution but reduces migration and invasion. ML-SI1 reduces androgen receptor (AR) protein stability and spermine levels in LNCaP cells, whereas increased spermine levels are observed in 22Rv1 cells at 24 h.

**Conclusions:**

*In vitro*, TRPML1 inhibition produces distinct cellular responses in LNCaP and 22Rv1 cells and is accompanied by differential changes in AR regulation and polyamine metabolism. Further mechanistic and *in vivo* studies are required to determine the biological and therapeutic relevance of these findings.

## 1 Introduction

Prostate cancer (PCa) is one of the most common malignancies in men (1), accounting for approximately 29% of newly diagnosed cancers among men in the United States in 2024. Worldwide, more than 1.4 million new cases and approximately 390,000 deaths have been estimated (2–4). PCa is characterized by early hormone dependence, subsequent hormone independence, metastasis, and drug resistance (5). Pharmacological treatment, surgery, and radiotherapy can effectively inhibit PCa growth and progression; however, for castration-resistant PCa, current treatments primarily slow disease progression, and patient survival is substantially reduced once PCa cells metastasize. Therefore, investigating the mechanisms underlying the malignant progression of PCa and developing drugs that effectively inhibit this progression are essential (6, 7).

Transient receptor potential (TRP) channels sense a wide range of cellular and environmental signals (8). The TRPML family of the TRP ion channel superfamily consists of TRPML1, TRPML2, and TRPML3 and is closely associated with cellular autophagy and energy metabolism *in vivo* (9, 10). Most studies have focused on TRPML1 because of its well-established association with human diseases, particularly cancer. In pancreatic cancer models, TRPML1 inhibition induces cancer cell death while altering cellular metabolic profiles, particularly through the regulation of spermidine-related pathways (11). In breast cancer models, pharmacological inhibition with ML-SI1 or genetic knockout prevents lysosomal iron release, resulting in reduced stem cell marker expression and mammosphere-forming capacity and ultimately suppressing tumor initiation and growth (12). In ovarian cancer models, TRPML1-mediated lysosomal exocytosis promotes cisplatin resistance by facilitating drug efflux. This process is accompanied by reduced intracellular levels of arginine, glutamate, cysteine, and creatine, indicating a potential association between arginine metabolism and TRPML1-driven chemoresistance (13). In contrast, TRPML1-activated lysosomal autophagy enhances energy metabolism, supports cancer cell metabolism, and contributes to drug resistance in cancer (14). However, the involvement of TRPML1 in PCa progression remains unexplored. Previous studies have largely focused on non-prostate malignancies, leaving it unclear whether TRPML1-mediated lysosomal signaling contributes to the AR-driven biology of PCa. This study combines database and omics analyses with functional perturbation approaches to investigate the role of TRPML1 in PCa progression. These analyses aim to clarify the functional and mechanistic relevance of TRPML1 in PCa and evaluate its potential as a therapeutic target.

Several TRPML inhibitors have been reported, including ML-SI1 (15), ML-SI3 (16), ML-SI4 (17), ML-SI5 (18), and ML-SI6 (19), although only ML-SI1 and ML-SI3 are commercially available. ML-SI3 consists of cis and trans isomers, with the trans isomer exhibiting greater activity than the cis isomer. The pure (−)-trans-ML-SI3 isomer potently inhibits TRPML1 and TRPML2 and exhibits weaker inhibitory activity against TRPML3, whereas the pure (+)-trans-ML-SI3 enantiomer inhibits TRPML1 but activates TRPML2 and TRPML3 (20). ML-SI1 is an inseparable racemic mixture of cis and trans isomers that inhibits TRPML1 at lower concentrations (IC50, 15 μM) and TRPML1 and TRPML3 at higher concentrations (IC50, 50 μM) (21).

This study examines the expression of TRPML family members in PCa using public datasets, followed by an investigation of the effects of TRPML1 inhibition in PCa cells. LNCaP and 22Rv1 cells are selected for subsequent experiments because they represent two commonly used AR-positive PCa models with distinct androgen-response characteristics. LNCaP cells are androgen-sensitive, whereas 22Rv1 cells are castration-resistant and express full-length AR and AR-V7 (22). Accordingly, these two cell lines are utilized to determine whether TRPML1 inhibition produces distinct cellular responses in androgen-sensitive and castration-resistant PCa models. Functional assays, transcriptomic analysis, qRT-PCR, western blotting, and spermine measurements are performed to characterize the phenotypic and molecular changes associated with ML-SI1 treatment.

## 2 Materials and methods

### 2.1 Cell culture

Human prostate cancer cell lines were obtained from commercial cell banks as follows: LNCaP (catalog no. SCSP-5021), 22Rv1 (catalog no. SCSP-5022), PC-3 (catalog no. TCHu158), and DU145 (catalog no. TCHu222) were obtained from the Cell Bank of the Chinese Academy of Sciences (Shanghai, China), whereas LNCaP-C4-2 (catalog no. iCell-h626) was obtained from Cellverse Co., Ltd. (Shanghai, China). All cell lines were authenticated by short tandem repeat (STR) profiling and confirmed to be free of mycoplasma contamination before use. Mycoplasma contamination was routinely monitored during culture, and only mycoplasma-negative cells were used in the experiments. PC-3 and DU145 cells were cultured in Dulbecco's modified Eagle's medium (Gibco, 11965092) supplemented with 10% fetal bovine serum (Gibco, A5256701). LNCaP, 22Rv1, and LNCaP-C4-2 cells were cultured in RPMI 1640 medium (Gibco, 11875093) supplemented with 10% fetal bovine serum (FBS; Gibco, A5256701). All cells were maintained at 37°C in a humidified incubator containing 5% CO_2_. For the experiments, cells in the logarithmic growth phase were harvested using 0.25% trypsin–EDTA (Gibco, 25200056) and seeded at appropriate densities. All media were supplemented with 1% penicillin–streptomycin (Gibco, 15140122) to prevent bacterial contamination. LNCaP and 22Rv1 cells were used as the principal experimental models to represent androgen-responsive and castration-resistant PCa, respectively.

### 2.2 MTT assay

Cell viability was assessed using the 3-(4,5-dimethylthiazol-2-yl)-2,5-diphenyltetrazolium bromide (MTT) assay. LNCaP and 22Rv1 cells were seeded in 96-well plates at a density of 5 × 10^3^ cells per well and treated with ML-SI1 at concentrations of 5, 10, 15, 20, 25, 30, 40, or 60 μM for 24 or 48 h. Untreated cells and dimethyl sulfoxide (DMSO)-treated cells served as the control and vehicle control groups, respectively. Following treatment, 10 μL of MTT solution (5 mg/mL; Beyotime, C0009S) was added to each well, and the plates were incubated for 3–5 h. The culture medium was then removed, and 150 μL of DMSO was added to each well to dissolve the formazan crystals. The plates were shaken for 10 min, after which absorbance was measured at 570 nm using a microplate reader. Cell viability was calculated by normalizing the absorbance values to those of the corresponding DMSO-treated control group, which was set to 100%.

### 2.3 CCK-8 assay

Cell viability was assessed using a Cell Counting Kit-8 (CCK-8) assay (GLPBIO, GK10001). Briefly, cells were seeded in 96-well plates at a density of 4,000 cells per well in 100 μL of complete culture medium and allowed to adhere overnight. The cells were then treated with the indicated compounds or vehicle control for the specified durations (24 and 48 h). At each time point, 10 μL of CCK-8 reagent was added to each well, and the plates were incubated at 37°C in the dark for 4 h. Absorbance was measured at 450 nm. Wells containing medium and CCK-8 reagent without cells served as blank controls, and their absorbance values were subtracted from the experimental readings. Cell viability was calculated by normalizing the absorbance values to those of the corresponding vehicle-treated control group, which was set to 100%.

### 2.4 Cell migration and invasion assay

For the migration assay, 5 × 10⁴ cells suspended in serum-free medium were seeded into the upper chamber of a Transwell insert (8 μm pore size; Corning Inc.), and medium containing 20% FBS was added to the lower chamber as a chemoattractant. After incubation at 37°C in 5% CO_2_ for 24 h, the Transwell chamber was removed, and the medium was discarded before the chamber was washed with calcium-free phosphate-buffered saline (PBS). For the invasion assay, the upper chamber was coated with Matrigel (Corning, 354230) and seeded with 1 × 10⁵ cells, whereas the lower chamber contained medium supplemented with 20% FBS. After incubation at 37°C in 5% CO_2_ for 24 h, the Transwell chamber was removed, and the medium was discarded before the chamber was washed with calcium-free PBS. The cells were fixed with methanol for 30 min and stained with 0.1% crystal violet for 20 min. Nonmigrated cells remaining on the upper surface of the membrane were gently removed using a cotton swab, and the migrated or invaded cells were counted under a microscope.

### 2.5 Cell-cycle analysis

Cell cycle distribution was analyzed using a Cell Cycle and Apoptosis Analysis Kit (Beyotime, C1052). After treatment, cells were harvested using 0.25% trypsin, collected by centrifugation at approximately 1,000 × g for 3 − 5 min, and washed once with PBS. The cells were fixed in ice-cold 70% ethanol at 4°C for 24 h and washed with PBS. Fixed cells were incubated with propidium iodide (PI; 50 μg/mL) and DNase-free RNase A (100 μg/mL) in PBS containing 0.1% Triton X-100 for 30 min at 37°C in the dark. PI fluorescence was measured by flow cytometry using 488 nm excitation. Cell doublets were excluded, and the percentages of cells in the G0/G1, S, and G2/M phases were determined using FlowJo software. Cell-cycle analysis was performed in both LNCaP and 22Rv1 cells following treatment with DMSO or 15 μM ML-SI1 for 24 h.

### 2.6 Generation of TRPML1-knockout cell lines

TRPML1 (MCOLN1) knockout cells were generated using a lentiviral CRISPR/Cas9 system. Lentiviral supernatant was added to the target cells in 1 mL of fresh complete medium supplemented with 3.2μL of polybrene (10 mg/mL) (Beyotime, C0351). After 24 h of incubation, an additional 1 mL of complete medium was added. At 72 h post-transduction, the medium was replaced, and the cells were subjected to puromycin selection (0.6 μg/mL; Beyotime, ST551) to establish a stable pooled knockout cell population. Following selection, the resulting cells were used in subsequent experiments.

sgTRPML1 oligos:

Forward: 5′-CACCGTGCGACAAGTTTCGAGCCAA-3′
Reverse: 5′-AAACTTGGCTCGAAACTTGTCGCAC-3′

### 2.7 Western blotting

Cells were lysed on ice for 30 min using cell lysis buffer (Beyotime, P0013) supplemented with 1% phenylmethylsulfonyl fluoride (Beyotime, ST505) to prevent proteolytic degradation. Following lysis, the samples were centrifuged at 12,000 × g for 10 min at 4°C, and the supernatants were collected for protein quantification using a bicinchoninic acid protein assay kit (Thermo Fisher Scientific, 23225). Protein samples were mixed with 5 ×  SDS loading buffer and boiled for 10 min. A total of 30 μg of protein from each sample was loaded onto 10% SDS-PAGE gels for electrophoretic separation, followed by transfer onto 0.45 μm polyvinylidene fluoride membranes (Merck, IPFL00010). The membranes were then blocked with 5% bovine serum albumin (Solarbio, A8020) for 1 h. Then, the membranes were incubated overnight with diluted antibodies on a shaker at 4°C, including anti-AR (Proteintech, 22089-1-AP), anti-AR-V7 (Abcam, ab198394), anti-LAMP1 (Thermo Fisher Scientific, MA5-29385), anti-β-actin (Proteintech, 81115-1-RR), and anti-GAPDH (Proteintech, 60004-1-Ig). The membranes were washed three times for 10 min each with TBS-T (0.1% Tween-20) at room temperature, incubated with goat anti-rabbit IgG H&L (HRP) or goat anti-mouse IgG H&L (HRP) for 1 h, and washed three times for 10 min each with TBS-T (0.1% Tween-20) at room temperature. The membranes were then exposed to an enhanced chemiluminescence substrate detection solution. Densitometric analyses of the western blots were performed using data from three independent biological experiments.

### 2.8 qRT-PCR analysis

Total RNA from various cancer cells and corresponding normal cells was isolated using RNAiso TRIzol reagent (TransGens Biotech, 15596026CN) and reverse-transcribed using PrimeScript™ RT Master Mix (TaKaRa, RR037Q) based on the manufacturer's instructions. qRT-PCR was performed using a QuantStudio 3 real-time fluorescent quantitative PCR system (Thermo Fisher Scientific). All qRT-PCR assays were performed in triplicate, and the results were normalized to GAPDH.

Relative fold expression was calculated using the comparative cycle threshold (2-*ΔΔ*Ct) method. All primer sequences used for qRT-PCR were as follows:

AR-F: AAAGAGCCGCTGAAGGGAAA
AR-R: AAGCCTCTCCTTCCTCCTGT
ODC1-F: CTGGGCGCTCTGAGATTGTC
ODC1-R: CCAGCTTCTCACAAAGGCAAC
SMS-F: GCCAAAGCTGATGGTGAGAC
SMS-R: TATGGGGTGGTAATCTGCCGT
GAPDH-F: AATGGGCAGCCGTTAGGAAA
GAPDH-R: GCCCAATACGACCAAATCAGAG

### 2.9 Transcriptomics analysis

LNCaP cell samples were collected 48 h after drug administration. The cells were lysed with TRIzol and stored at −80°C. The samples were transported on dry ice to Wuhan Huada Genetics for transcriptomic and statistical analyses. Statistical results were obtained from the official website of Wuhan Huada Genetics (https://biosys.bgi.com/#/report/login).

### 2.10 Spermine content measurement

Spermine content was measured using a Human Spermine enzyme-linked immunosorbent assay kit (COI BO Biologicals, CB13658-Hu). LNCaP and 22Rv1 cells were seeded in 24-well plates at 2 × 10⁵ cells per well in 600 μL of medium and allowed to grow overnight before treatment with ML-SI1 (MCE, HY-134818) for 24 or 48 h. Cell supernatants were collected in microcentrifuge tubes and centrifuged at 3000 rpm for 10 min to remove pellets and polymers, after which the resulting supernatants were processed for the assay. Adherent cells were gently washed with cold PBS, digested with trypsin, and collected by centrifugation at 1000 × g for 5 min. The collected cells were washed three times with cold PBS and disrupted ultrasonically in an ice bath. The extracts were centrifuged at 1500 × g for 10 min, and the supernatants were collected for detection. Detection reagents were added according to the prescribed protocol, and the optical density of each well was measured at 450 nm.

### 2.11 Statistical analysis

All experiments were repeated at least three times. Data are presented as the mean ± SEM. The statistical significance of differences was evaluated using analysis of variance (ANOVA), followed by Tukey's test. Statistical significance was defined as *p* < 0.05.

## 3 Results

### 3.1 Expression of TRPML genes in tumor tissues

The Human Protein Atlas (HPA; https://www.proteinatlas.org/) was first queried to examine the tissue distribution of TRPML1, TRPML2, and TRPML3 at the RNA and protein levels to investigate the expression patterns of TRPML family members (Supplementary Figures S1A–D). Among the three TRPML family members, TRPML1 exhibited relatively broad expression across human tissues and organs, whereas TRPML2 and TRPML3 exhibited more restricted expression patterns (Supplementary Figures S1B–D). HPA immunohistochemistry data for TRPML1, TRPML2, and TRPML3 were examined across different cancer types (Figures 1A–C). The data presented in Figure 1 represent the percentage of patients with detectable positive protein staining and therefore reflect protein-level expression rather than mRNA abundance. TRPML1 and TRPML2 exhibited relatively high protein-positive staining rates in several cancer types compared to TRPML3. PCa was among the cancer types with high proportions of TRPML1- and TRPML2-positive samples, with positive staining observed in approximately 80% and 90% of cases, respectively. These HPA data indicate that TRPML1 and TRPML2 proteins are frequently detectable in PCa tissues and provide a rationale for further analysis of TRPML family expression in PCa.

**Figure 1:**
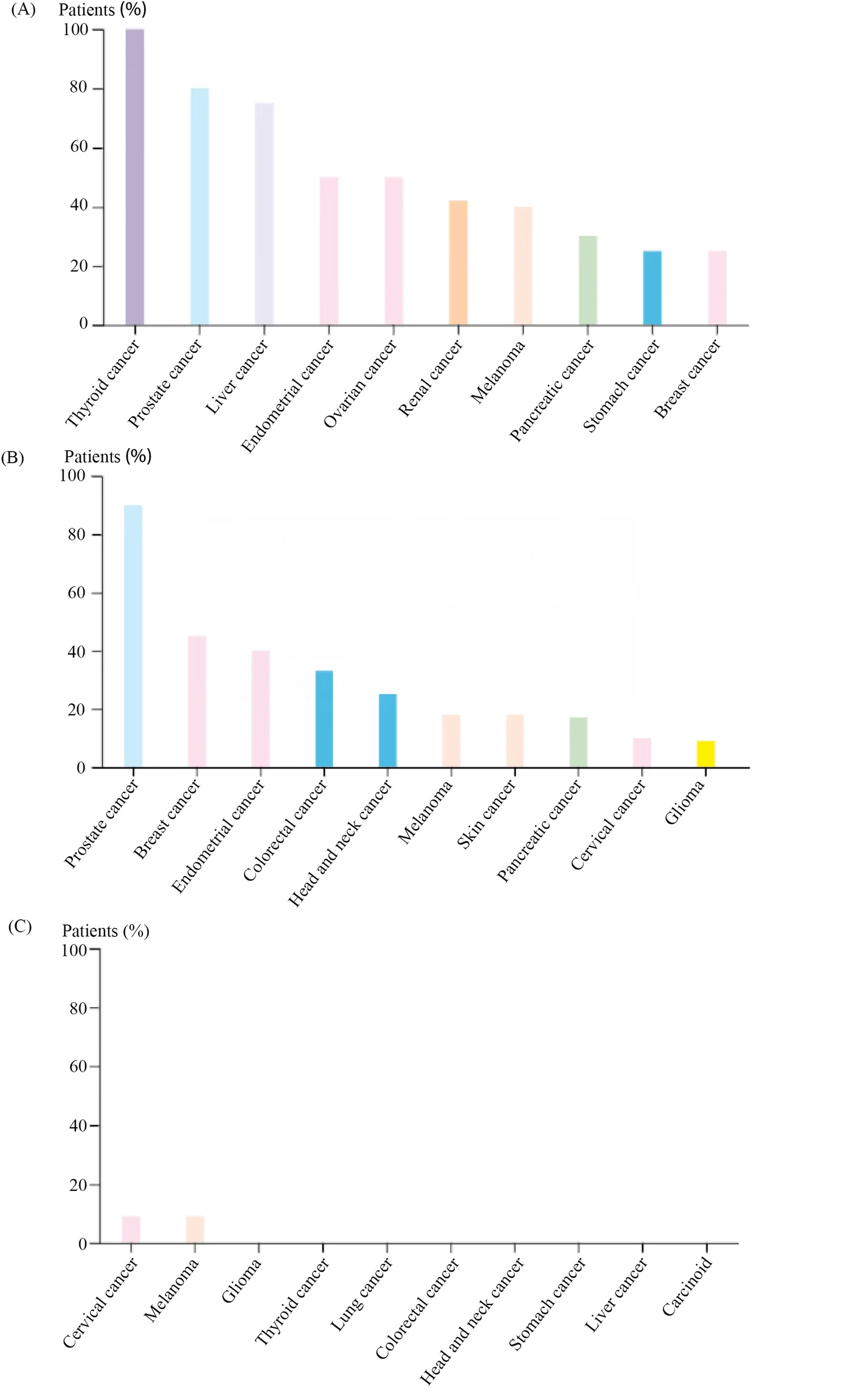
Protein expression of (**A**) TRPML1, (**B**) TRPML2, and (**C**) TRPML3 across cancer types based on Human Protein Atlas (HPA) immunohistochemistry data. The y-axis represents the percentage of patients with detectable protein-positive staining. Reproduced from the Human Protein Atlas (HPA), licensed under CC BY 4.0. Image credit: Human Protein Atlas. Sources: MCOLN1, https://www.proteinatlas.org/ENSG00000090674-MCOLN1/cancer; MCOLN2, https://www.proteinatlas.org/ENSG00000153898-MCOLN2/cancer; MCOLN3, https://www.proteinatlas.org/ENSG00000055732-MCOLN3/cancer.

Because the HPA immunohistochemistry data reflect protein-level staining, the mRNA expression of TRPML family members was examined separately using The Cancer Genome Atlas (TCGA) dataset. TRPML1 and TRPML2 expression levels were higher in PCa tissues than in adjacent normal tissues, whereas TRPML3 expression was comparable between the groups (Figures 2A–C). TRPML1 and TRPML2 expression levels were also comparable between the primary PCa (G1) and metastatic/recurrent PCa (G2) groups (Figures 2A, B). When the three TRPML family members were visualized together, TRPML1 showed relatively higher expression levels than TRPML2 and TRPML3 in this dataset (Figure 2D). These expression patterns provided a rationale for further examining TRPML1 in PCa.

**Figure 2:**
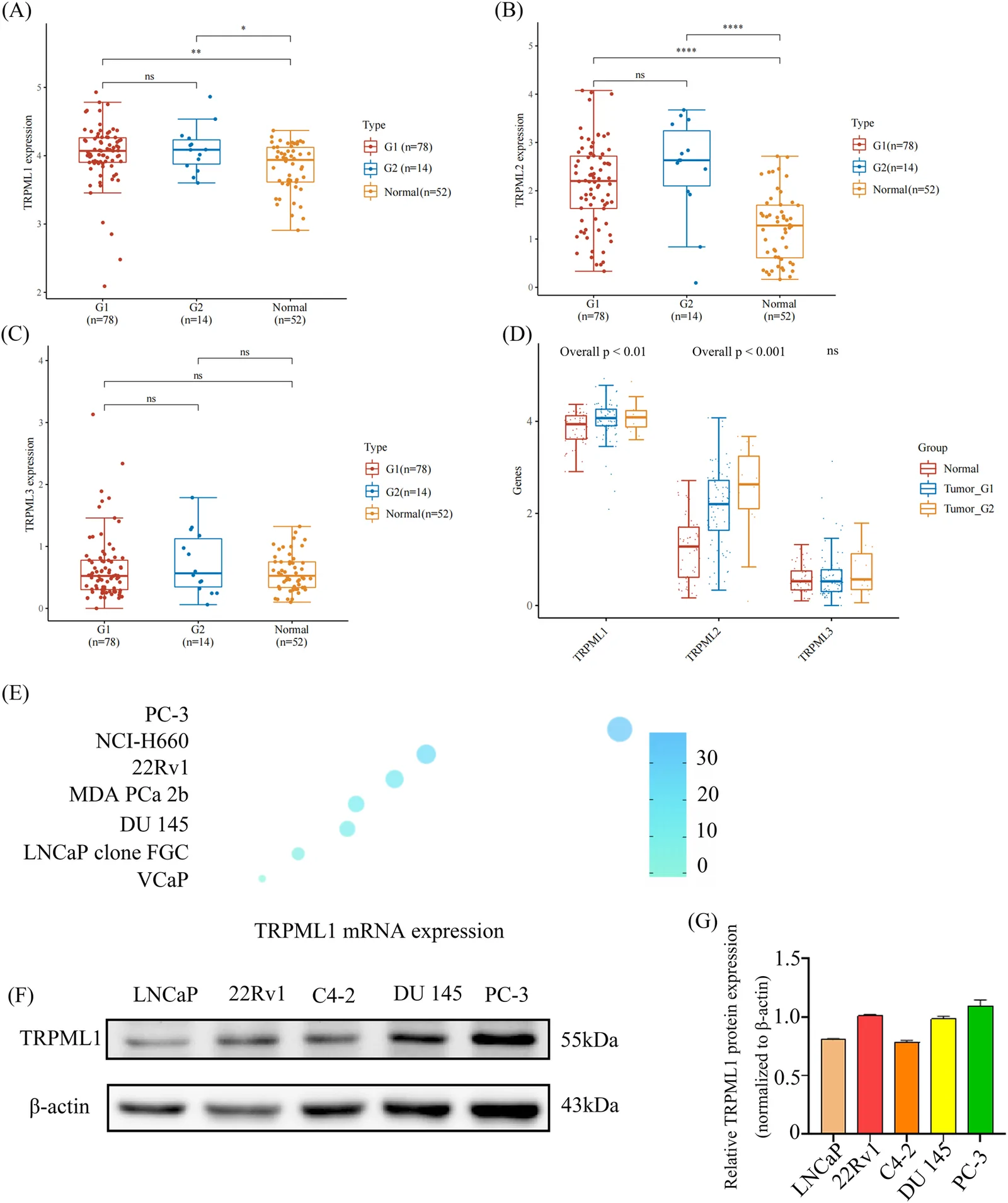
Expression of TRPML family members in prostate cancer tissues and cell lines. **(A–D)** TRPML1–3 mRNA expression based on TCGA data. Samples were grouped as primary prostate cancer (G1), metastatic/recurrent prostate cancer (G2), and adjacent normal tissues. Statistical significance is indicated as *p < 0.05, **p < 0.01, ****p < 0.0001, and ns, not significant. Pairwise comparisons were performed using the Wilcoxon rank-sum test, whereas overall differences among G1, G2, and adjacent normal tissues for each gene in **(D)** were assessed using the Kruskal–Wallis test. **(A)** TRPML1 mRNA expression. **(B)** TRPML2 mRNA expression. **(C)** TRPML3 mRNA expression. **(D)** Comparative expressions of TRPML1, TRPML2, and TRPML3. **(E)** MCOLN1 (TRPML1) mRNA expression across seven prostate cancer cell lines in the CCLE database: PC-3, NCI-H660, 22Rv1, MDA PCa 2b, DU145, LNCaP clone FGC, and VCaP. **(F)** TRPML1 protein expression in LNCaP, 22Rv1, C4-2, DU145, and PC-3 cells. **(G)** Densitometric analysis of TRPML1 protein expression **(F)**, normalized to β-actin.

Next, TRPML1 mRNA expression was examined in seven prostate cancer cell lines in the Cancer Cell Line Encyclopedia (CCLE) database, including PC-3, NCI-H660, 22Rv1, MDA PCa 2b, DU145, LNCaP clone FGC, and VCaP (Figure 2E). TRPML1 expression varied among these cell lines, with relatively higher levels observed in PC-3 and NCI-H660 cells and lower levels observed in LNCaP clone FGC and VCaP cells. TRPML1 protein expression was examined in LNCaP, 22Rv1, C4-2, DU145, and PC-3 cells (Figures 2F, G). TRPML1 protein levels also varied among these cell lines. Thus, TRPML1 expression was heterogeneous across prostate cancer cell models, and these findings alone do not establish a linear relationship between TRPML1 expression and malignant progression.

### 3.2 ML-SI1 inhibits proliferation and metastasis in LNCaP cells

The effects of the TRPML1 inhibitor ML-SI1 were evaluated in LNCaP cells *in vitro*. LNCaP cells were treated with ML-SI1 at concentrations ranging from 5 to 60 μM, and cell viability was assessed by MTT assay after 24 and 48 h. Cell viability remained comparable to that of the DMSO control at 5 and 10 μM, whereas higher concentrations progressively reduced cell viability at both time points. At 20 μM, cell viability was reduced to 83.17% ± 0.92% at 24 h and 82.44% ± 1.68% at 48 h, and further decreased to 51.02% ± 1.09% and 50.07% ± 1.03%, respectively, at 60 μM (Figures 3A, B). Cell-cycle analysis revealed that treatment with 15 μM ML-SI1 for 24 h increased the proportion of LNCaP cells in the G0/G1 phase and decreased the proportion of cells in the S phase, whereas no statistically significant change was observed in the G2/M phase (*p* > 0.05; Figure 3C). These findings indicate that ML-SI1 induces cell cycle arrest in the G0/G1 phase. Cell viability was compared between parental LNCaP cells and TRPML1-knockout (KO-TRPML1) LNCaP cells treated with DMSO or ML-SI1 (20 μM) using a CCK-8 assay to determine whether the antiproliferative effect of ML-SI1 was mediated by TRPML1. In parental LNCaP cells, ML-SI1 treatment reduced cell viability to 83.79% ± 10.42% at 24 h and 84.08% ± 4.61% at 48 h relative to the DMSO control (Figures 3D, E). In contrast, TRPML1 knockout alone markedly decreased cell viability to 35.33% ± 3.76% at 24 h and 38.43% ± 10.62% at 48 h (Figures 3D, E). ML-SI1 treatment did not further reduce viability in KO-TRPML1 cells, with cell viability remaining at 38.92% ± 2.41% at 24 h and 38.35% ± 6.88% at 48 h relative to the KO-TRPML1 + DMSO group (Figures 3D, E). Successful ablation of TRPML1 in knockout LNCaP cells was further confirmed by immunoblotting (Figure 3F). Therefore, these results indicate that the growth-inhibitory effects of ML-SI1 in LNCaP cells are TRPML1-dependent. The results of the Transwell cell migration assay showed that ML-SI1 effectively inhibited LNCaP cell migration at concentrations of 10 and 15 μM in a dose-dependent manner, with a maximum inhibition rate of 55.5% ± 4.02% (Figures 3G, H). Therefore, ML-SI1 effectively inhibited the proliferation of LNCaP cells at high doses and their migratory capacity at low doses. These results show that the proliferative and migratory abilities of hormone-dependent PCa cells are suppressed by inhibition of TRPML1 activity.

**Figure 3:**
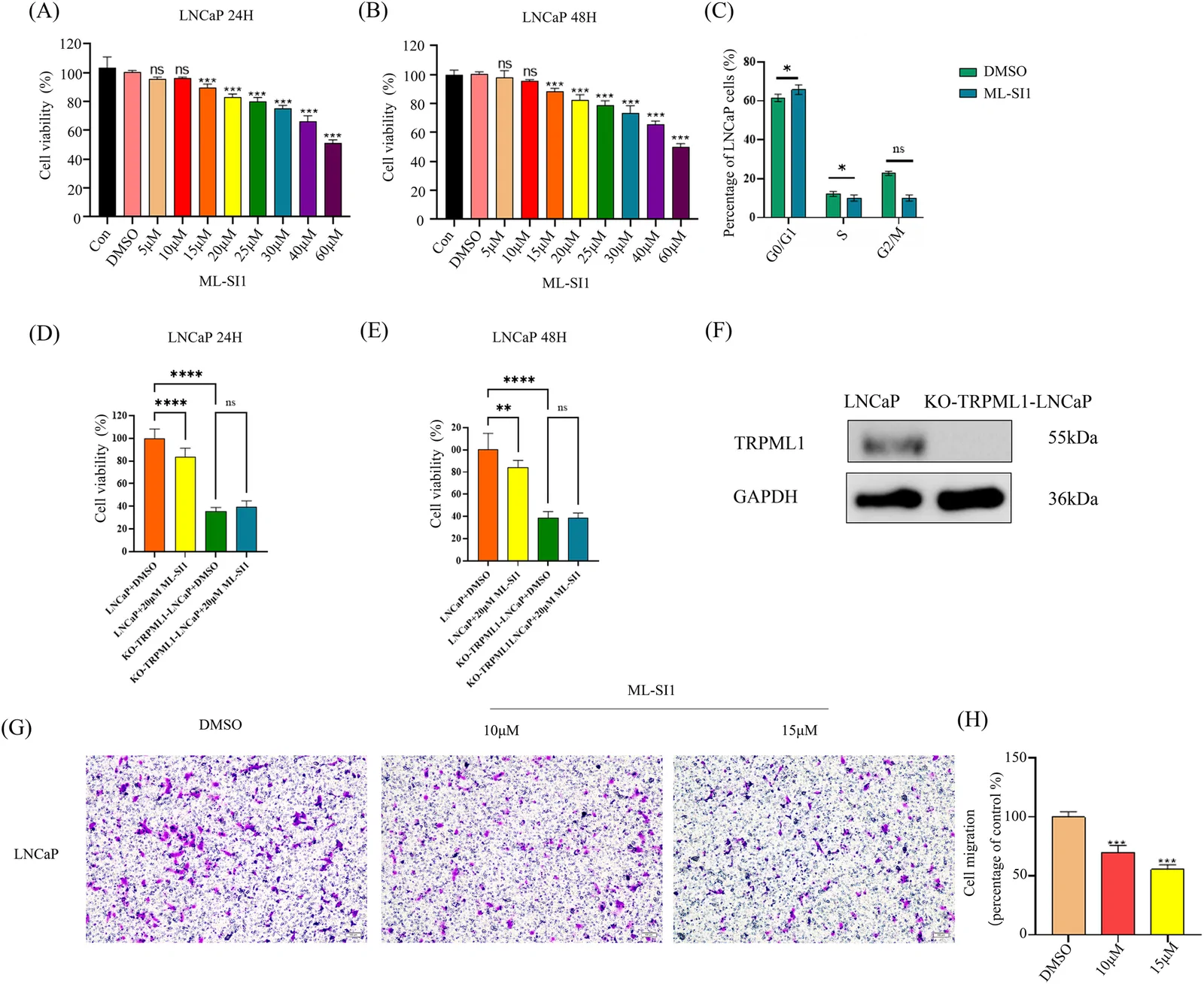
MTT experiments of ML-SI1 effects on LNCaP prostate cancer cells. Results of MTT experiments with LNCaP cells treated for **(A)** 24 and **(B)** 48 h. **(C)** Cell-cycle experiments of ML-SI1 effects on LNCaP cells. **(D)** CCK-8 analysis of LNCaP cell viability at 24 h under DMSO, ML-SI1 (20 μM), KO-TRPML1 + DMSO, and KO-TRPML1 + ML-SI1 (20 μM) conditions. *p < 0.05, **p < 0.01, ***p < 0.001, compared to the LNCaP + DMSO group. **(E)** CCK-8 analysis of LNCaP cell viability at 48 h under the indicated conditions. *p < 0.05, **p < 0.01, ***p < 0.001, compared to the LNCaP + DMSO group. **(F)** Immunoblotting confirming TRPML1 depletion in KO-TRPML1 LNCaP cells. **(G)** Representative images of LNCaP cell migration. Scale bars, 100 μm. **(H)** Statistical analysis of **(G)** *p < 0.05, **p < 0.01, ***p < 0.001, compared to the control group.

### 3.3 Effects of ML-SI1 on 22Rv1 cells

The effects of ML-SI1 on 22Rv1 cells were examined. 22Rv1 cells were treated with ML-SI1 at concentrations ranging from 5 to 60 μM, and cell viability was assessed by MTT assay after 24 and 48 h. ML-SI1 produced no clear dose-dependent reduction in 22Rv1 cell viability across the tested concentration range at either time point (Figures 4A, B). Cell-cycle distribution was examined by flow cytometry following treatment with DMSO or 15 μM ML-SI1 for 24 h. No statistically significant differences were observed in the percentages of cells in the G0/G1, S, or G2/M phases between the two groups (all *p* > 0.05; Supplementary Figure S2). These results are consistent with the limited effect of ML-SI1 on 22Rv1 cell proliferation under the tested conditions. In contrast, treatment with 20 μM ML-SI1 for 24 h reduced 22Rv1 cell migration (Figures 4C, D), with a maximum inhibition rate of 59.4% ± 0.27%. ML-SI1 also inhibited 22Rv1 cell invasion, with a maximum inhibition rate of 44.2% ± 4.79% (Figures 4C, E). TRPML1-knockout (KO-TRPML1) 22Rv1 cells were generated, and migration was assessed using a Transwell migration assay at 24 h to determine whether the inhibitory effect of ML-SI1 on cell migration was TRPML1-dependent. The four groups were 22Rv1 + DMSO, 22Rv1 + ML-SI1 (20 μM), KO-TRPML1 22Rv1 + DMSO, and KO-TRPML1 22Rv1 + ML-SI1 (20 μM). Representative images showed that ML-SI1 treatment reduced the number of migrated parental 22Rv1 cells compared to the DMSO control (Figure 4F). Quantification indicated that ML-SI1 decreased migration to 76.26% ± 9.74% relative to the control group at 24 h (Figure 4G). In contrast, TRPML1 knockout alone resulted in a more pronounced reduction in migration, decreasing it to 32.16% ± 11.44% (Figure 4G). ML-SI1 treatment of KO-TRPML1 cells resulted in migration of 33.65% ± 8.52%, with no apparent difference compared to the KO-TRPML1 + DMSO group (Figure 4G). Successful depletion of TRPML1 in knockout 22Rv1 cells was confirmed by immunoblotting (Figure 4H). Therefore, these results indicate that ML-SI1 reduced migration and invasion in 22Rv1 cells despite its limited effects on cell viability and cell cycle distribution. Together with the results obtained in LNCaP cells, these findings demonstrate that the cellular response to ML-SI1 differs between the two models, with broader effects on proliferation and migration in LNCaP cells and predominantly antimigratory and anti-invasive effects in 22Rv1 cells.

**Figure 4:**
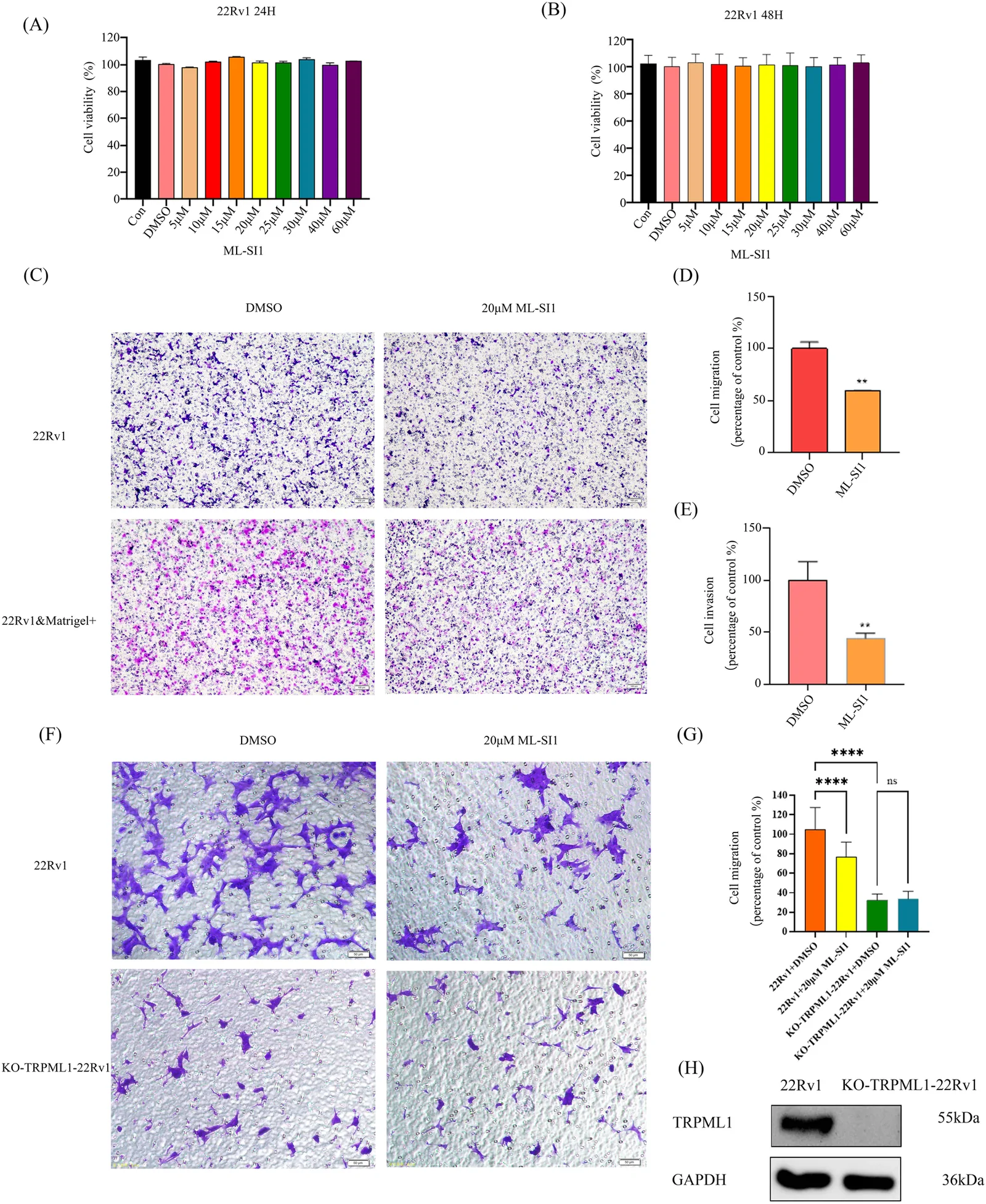
MTT experiments of ML-SI1 effects on 22Rv1 prostate cancer cells. Results of MTT experiments with 22Rv1 cells treated for **(A)** 24 and **(B)** 48 h. **(C)** Representative images of migration and invasion of 22Rv1 cells. Scale bars, 100 μm. **(D,E)** Statistical analysis of **(C)** **p < 0.01, ****p < 0.0001, compared to the control group. **(F)** Representative images of Transwell migration at 24 h in 22Rv1 cells treated with DMSO, ML-SI1 (20 μM), KO-TRPML1 + DMSO, and KO-TRPML1 + ML-SI1 (20 μM). Scale bars, 50 μm. **(G)** Quantification of migrated cells at 24 h under the indicated conditions, normalized to the control. **(H)** Immunoblotting confirming TRPML1 depletion in KO-TRPML1 22Rv1 cells.

### 3.4 ML-SI1 alters polyamine metabolism-related gene expression in LNCaP cells

Because ML-SI1 inhibited the proliferation and migration of LNCaP cells, transcriptomic analysis was performed to identify molecular changes associated with ML-SI1 treatment. A total of 1,700 differentially expressed genes were identified after 48 h of treatment, including 938 upregulated and 762 downregulated genes (Supplementary Figures S3A–C). The 20 most strongly upregulated and downregulated genes were examined (Figure 5A). Among the downregulated genes, KLK3 is a well-established marker of prostate cancer and AR signaling, MPC2 is involved in mitochondrial energy metabolism, and ODC1 and SMS encode key enzymes involved in polyamine biosynthesis (23, 24). These transcriptomic changes indicated that AR-related signaling, cellular metabolism, and polyamine metabolism can be affected by ML-SI1 treatment, warranting further examination of these pathways in subsequent experiments.

**Figure 5:**
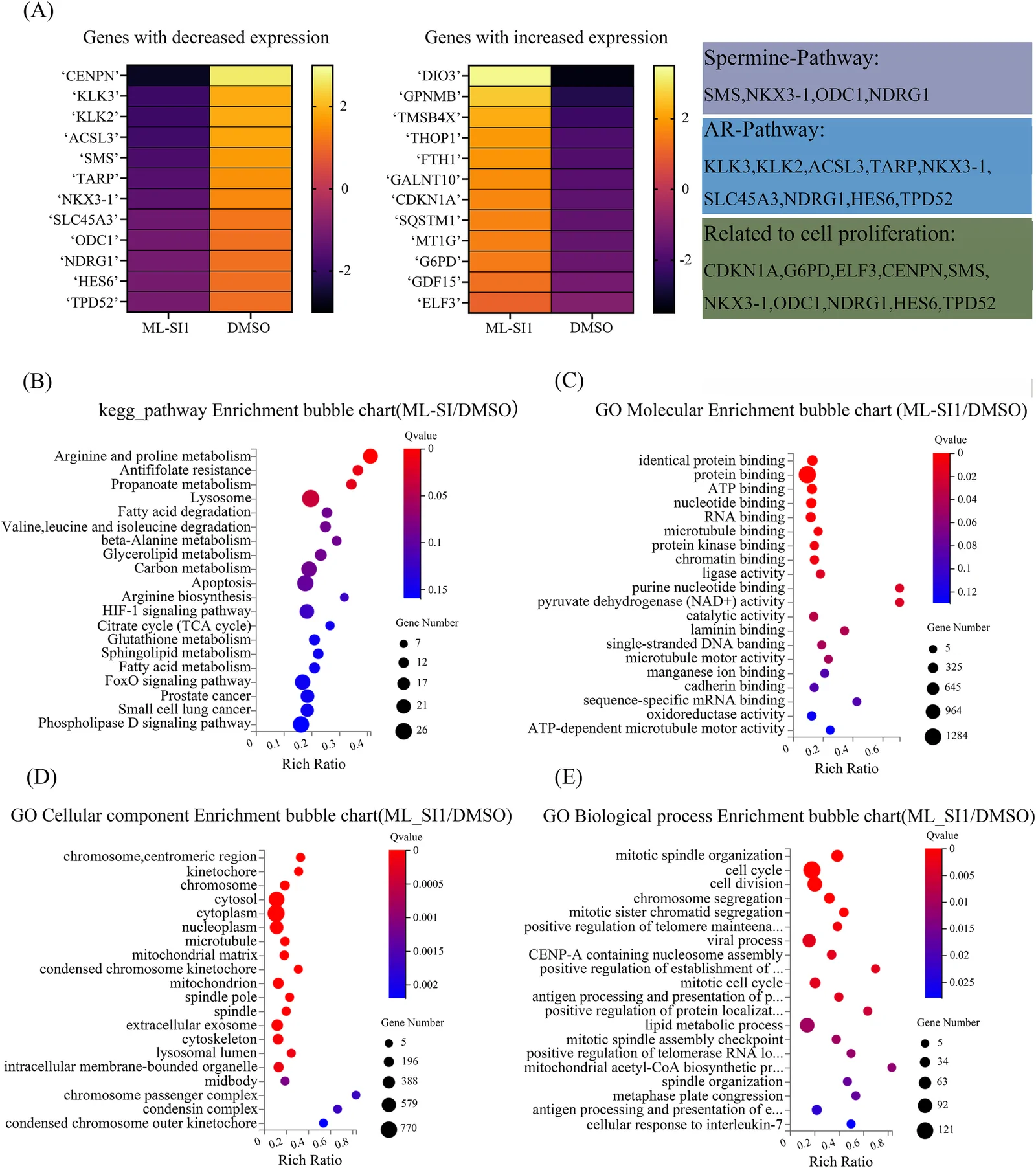
Transcriptomic analysis of the effect of ML-SI1 on LNCaP cells. **(A)** Heat map analysis of significantly downregulated and upregulated genes between ML-SI1-treated (20 μM, 48 h) and DMSO control (vehicle control) groups. Genes involved in the AR pathway, spermine pathway, and cell proliferation are listed. **(B)** KEGG enrichment analysis. **(C)** Molecular function (MF), **(D)** CC, and **(E)** BP enrichment analyses in the GO data map.

Kyoto Encyclopedia of Genes and Genomes (KEGG) metabolic signaling pathway enrichment and Gene Ontology (GO) molecular function (MF), cellular component (CC), and biological process (BP) enrichment analyses were performed using the UW Genetics online website to clarify the mechanisms associated with ML-SI1 treatment in LNCaP cells (Figures 5B–E). In the KEGG enrichment map, the four most highly enriched signaling pathways were arginine and proline metabolism, antifolate resistance, propionate metabolism, and lysosomal pathways. The pathways identified by KEGG enrichment analysis were primarily associated with lysosome-related amino acid metabolism, drug resistance, and energy metabolism. In addition, arginine metabolism, which is closely linked to polyamine biosynthesis, was among the highly enriched pathways, further supporting the possibility that ML-SI1 affects polyamine-related metabolism in LNCaP cells. Based on the GO enrichment results, the molecular functions affected by ML-SI1 in LNCaP cells were primarily associated with protein assembly and ATP-related functions. Regarding cellular components, the enriched genes were associated with structures involved in mitosis, including the mitotic spindle and chromosomes; these structures are primarily located in the nucleus and are crucial for cell proliferation and division. Differentially expressed genes were also enriched in biological processes related to cell proliferation, including mitotic spindle organization, the cell cycle, cell division, and chromosome segregation.

Transcriptomic analysis showed that ML-SI1 treatment was associated with changes in genes and pathways related to cell proliferation, amino acid metabolism, and polyamine metabolism (Figures 5A–E). These data identified candidate pathways for investigation; however, they did not independently establish a causal relationship between TRPML1 and AR or polyamine metabolism. Therefore, AR expression and polyamine-related changes were examined in LNCaP cells.

### 3.5 Effect of ML-SI1 on AR expression in PCa cells

LNCaP and 22Rv1 cells were treated with 20 μM ML-SI1, and AR mRNA and protein levels were measured to examine the effect of ML-SI1 on AR expression. AR mRNA levels transiently increased in LNCaP cells after 8 h and returned to levels comparable to those of the control by 24 h (Figure 6A). In contrast, AR protein abundance was reduced by more than 50% following ML-SI1 treatment (Figures 6B, C). The difference between the mRNA and protein responses indicated that the reduction in AR protein abundance was not attributable to decreased AR transcription and prompted further examination of AR protein stability. In 22Rv1 cells, ML-SI1 did not significantly alter the protein levels of either full-length AR or AR-V7 (Supplementary Figure S4). Thus, the reduction in AR protein observed in LNCaP cells was not evident in 22Rv1 cells.

**Figure 6:**
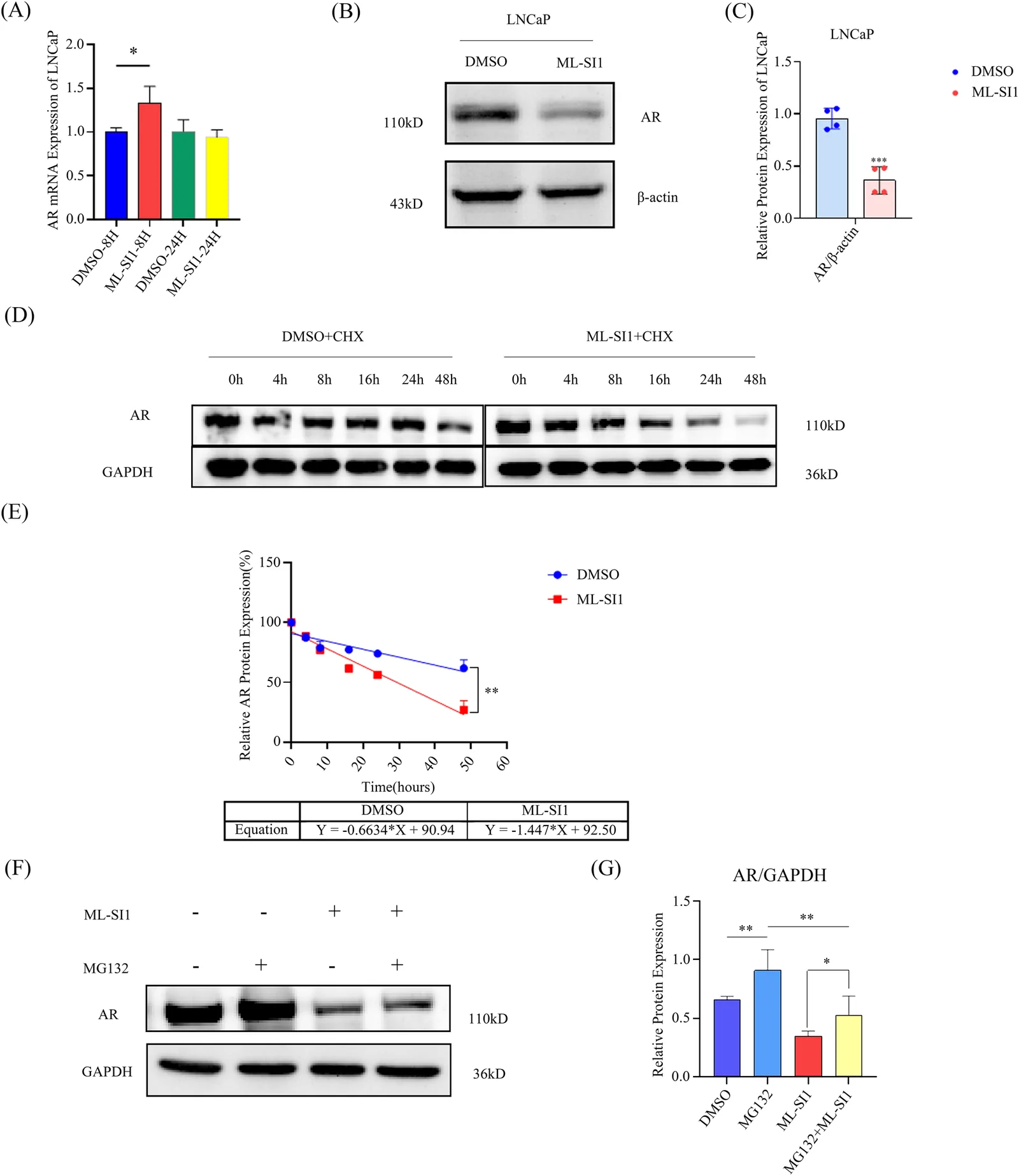
Effect of ML-SI1 on the expression of AR and related proteins in LNCaP cells. **(A)** AR mRNA changes in LNCaP cells. **(B)** AR protein expression changes in LNCaP cells. (**C**) Statistical analysis of (**B**). (**D**) AR half-life under different conditions. (**E**) Statistical analysis of (**D**). (**F**) AR protein degradation pathway assessment under different conditions. (**G**) Statistical analysis of (**F**). *p < 0.05, **p < 0.01, ***p < 0.001, compared to the control group.

AR protein stability was examined using a cycloheximide (CHX)-chase assay. Following inhibition of protein synthesis with CHX, the apparent half-life of AR was longer than 50 h in control LNCaP cells but was reduced to approximately 30 h in cells treated with 20 μM ML-SI1 (Figures 6D, E), corresponding to an approximately 40% reduction in the apparent AR half-life. These results indicate that ML-SI1 accelerates AR protein turnover in LNCaP cells.

LNCaP cells were treated with the proteasome inhibitor MG132 to determine whether proteasomal degradation contributed to the ML-SI1-induced reduction in AR. MG132 alone increased AR protein abundance by approximately 40% compared to the DMSO control, whereas ML-SI1 alone reduced AR protein abundance by approximately 40% (Figures 6F, G). In cells treated with MG132 and ML-SI1, AR abundance remained lower than that in the DMSO- and MG132-treated groups; however, was approximately 30% higher than that in cells treated with ML-SI1 alone. Thus, MG132 partially reversed the ML-SI1-induced decrease in AR protein abundance. These findings indicate that proteasomal degradation contributes to AR turnover under these conditions. However, the incomplete recovery of AR indicates that additional mechanisms can also be involved, and the present experiments do not identify the other degradation pathway (s).

### 3.6 Effect of ML-SI1 on polyamine metabolic pathways in LNCaP and 22Rv1 PCa cells

Polyamines, including putrescine, spermidine, and spermine, participate in multiple cellular processes. Spermine is the final product of the polyamine biosynthetic pathway and is generated from ornithine through a series of enzymatic reactions involving ODC1 and SMS. Intracellular and extracellular spermine levels were measured, and the expression of ODC1 and SMS, two key enzymes involved in polyamine biosynthesis, was analyzed to examine whether ML-SI1 altered polyamine metabolism in prostate cancer cells.

Intracellular and extracellular spermine levels were significantly reduced in LNCaP cells after 24 and 48 h of treatment with 20 μM ML-SI1 (all *p* < 0.05; Figures 7A, B). ODC1 mRNA expression did not change significantly at 24 h (*p* > 0.05), whereas SMS mRNA expression increased (*p* < 0.05; Figures 7C, D). At 48 h, both ODC1 and SMS mRNA levels were reduced (both *p* < 0.01; Figures 7C, D). ODC1 protein abundance decreased after 24 h (*p* < 0.05) and 48 h (*p* < 0.001), whereas SMS protein abundance decreased at both time points (both *p* < 0.05; Figures 7E–H). These results demonstrate that ML-SI1 treatment is associated with a sustained decrease in spermine levels and reduced ODC1 and SMS protein abundance in LNCaP cells.

**Figure 7:**
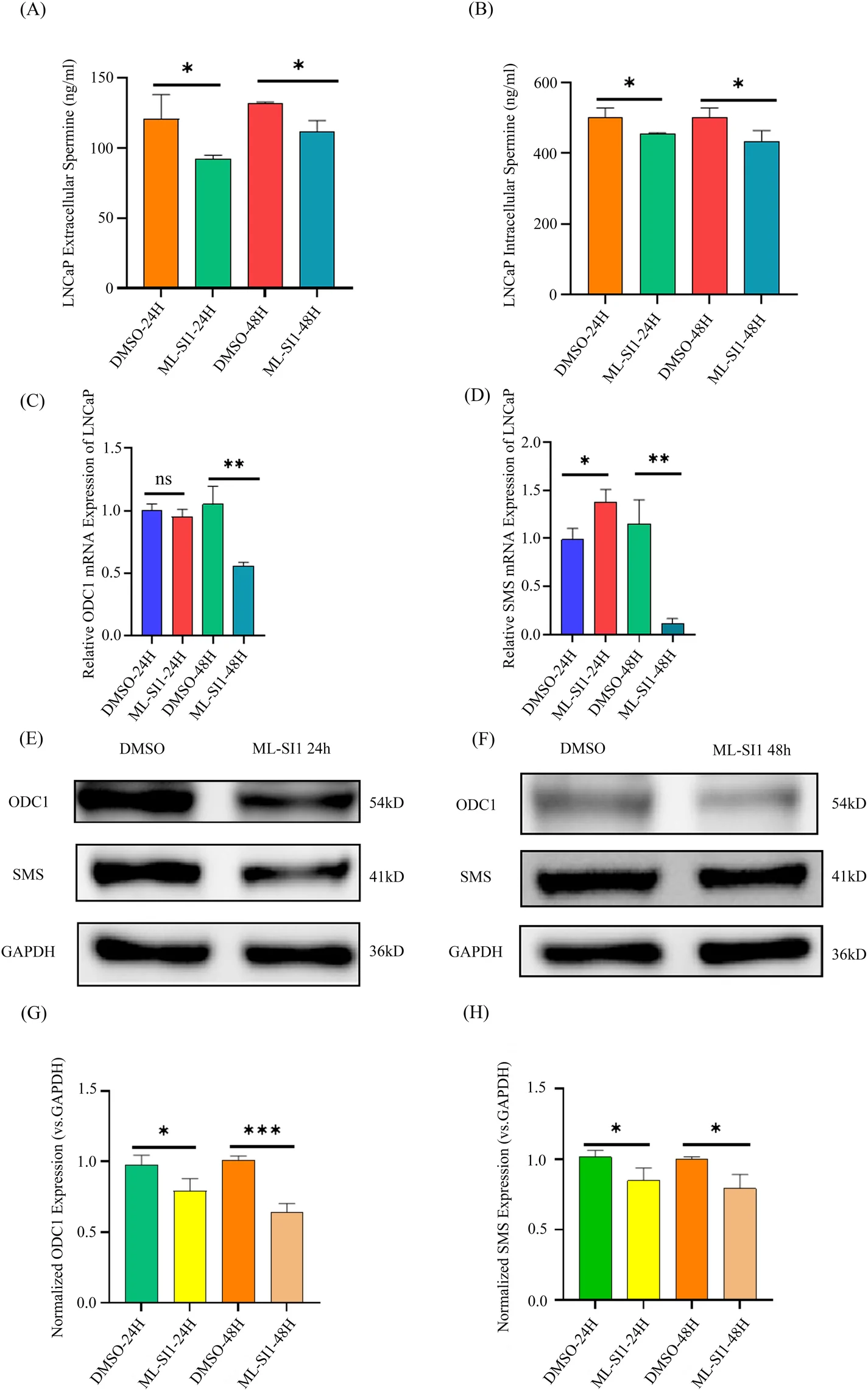
Intracellular and extracellular spermine contents in LNCaP cells and evaluation of the polyamine metabolic pathway ODC1 and SMS mRNAs and relative protein expression. **(A,B)** Spermine concentrations. **(C)** ODC1 mRNA expression in LNCaP cells after ML-SI1 treatment. **(D)** SMS mRNA expression in LNCaP cells after ML-SI1 treatment. **(E,F)** ODC1 and SMS protein expression after ML-SI1 treatment for 24 and 48 h. **(G,H)** Densitometric analysis of ODC1 and SMS protein expression normalized to GAPDH. *p < 0.05, **p < 0.01, ***p < 0.001, compared to the model group.

Intracellular and extracellular spermine levels in 22Rv1 cells increased following treatment with 20 μM ML-SI1 for 24 h (Figures 8A, B). ODC1 and SMS mRNA levels also increased at 24 h (Figures 8C, D), and ODC1 and SMS protein abundance increased following ML-SI1 treatment (Figures 8E–H). Thus, in contrast to the response observed in LNCaP cells, ML-SI1 treatment in 22Rv1 cells was associated with increased spermine levels and increased expression of ODC1 and SMS.

**Figure 8:**
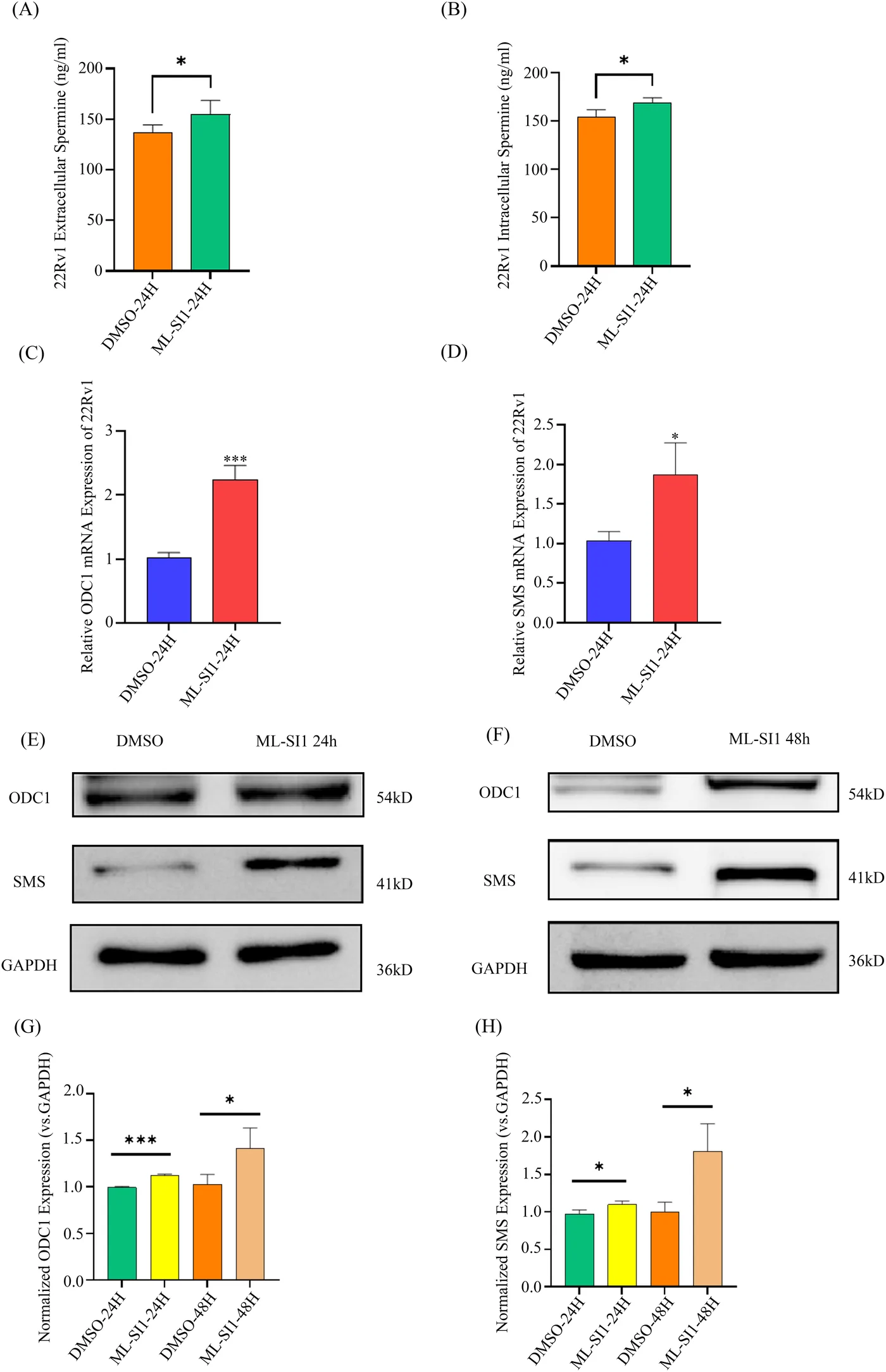
Intracellular and extracellular spermine contents in 22Rv1 cells and evaluation of polyamine metabolic pathway ODC1 and SMS mRNAs and relative protein expression. **(A,B)** Spermine concentrations. **(C)** ODC1 mRNA expression in 22Rv1 cells after ML-SI1 treatment. **(D)** SMS mRNA expression in 22Rv1 cells after ML-SI1 treatment. **(E,F)** ODC1 and SMS protein expression after ML-SI1 treatment for 24 and 48 h. **(G,H)** Densitometric analysis of ODC1 and SMS protein expression normalized to GAPDH. *p < 0.05, ***p < 0.001, compared to the model group.

## 4 Discussion

Prostate cancer (PCa) is one of the most prevalent malignancies in men, with its incidence strongly associated with age, particularly among men older than 65 years. Despite extensive research, the underlying causes of PCa remain incompletely understood and likely involve a complex interplay among genetic, hormonal, environmental, and psychological factors. Therapeutic options for advanced PCa remain limited because current approaches, such as combined antiandrogen therapy and chemotherapy, provide only transient suppression of tumor growth and progression. These challenges highlight the necessity to identify key signaling pathways and molecular targets that drive PCa malignancy and provide a rationale for the development of more effective and targeted therapeutic strategies.

TRPML1 is a lysosomal ion channel involved in autophagy and lysosomal homeostasis, both of which are processes associated with tumor development and treatment resistance (12, 14, 25, 26). TRPML1 expression in PCa has not been systematically examined. Analysis of public datasets in this study showed that TRPML1 expression was higher in prostate cancer tissues than in adjacent normal tissues. However, no statistically significant difference was detected between primary and metastatic/recurrent tumors in the TCGA dataset. In contrast, TRPML1 expression varied considerably among the prostate cancer cell lines analyzed in the CCLE dataset and in the protein expression analysis conducted in the present study. These findings should not be interpreted as contradictory evidence regarding disease progression because the cell lines do not constitute a defined malignancy series and differ substantially in their molecular backgrounds. In addition, clinical tumor samples and established cell lines represent distinct biological settings, and the heterogeneity present in patient tumors cannot be reproduced in individual cultured cell models. Accordingly, the findings indicate that TRPML1 expression is heterogeneous across prostate cancer models rather than progressively increasing with malignancy. The current data therefore do not support TRPML1 expression as a biomarker of clinical progression. However, pharmacological inhibition or genetic depletion of TRPML1 reduced selected tumor-related phenotypes in LNCaP and 22Rv1 cells, indicating that TRPML1 can contribute to the regulation of prostate cancer cell behavior in these models. However, its relationship with clinical disease progression remains to be established.

Transcriptomic analysis of LNCaP cells identified changes in genes related to AR signaling and polyamine metabolism following ML-SI1 treatment, prompting further examination of these pathways. In LNCaP cells, ML-SI1 reduced AR protein abundance without a corresponding decrease in AR mRNA levels. The CHX-chase assay further demonstrated that the apparent half-life of AR was shortened following ML-SI1 treatment, indicating increased AR protein turnover. MG132 partially restored AR protein levels, indicating that proteasomal degradation contributes to this effect, although the incomplete recovery of AR indicates that additional mechanisms can also be involved. A different response was observed in 22Rv1 cells. The 22Rv1 cell line is a castration-resistant prostate cancer model that expresses full-length AR and the constitutively active splice variant AR-V7 (27). No statistically significant change in AR or AR-V7 protein abundance was observed in 22Rv1 cells following ML-SI1 treatment, whereas AR protein abundance was markedly reduced in LNCaP cells. This difference may be relevant to the distinct effects of ML-SI1 on proliferation in the two cell models. In particular, persistent AR-V7 signaling may contribute to the reduced dependence of 22Rv1 cells on androgen-responsive pathways. However, AR-V7 was not directly manipulated in this study; therefore, the data do not establish AR-V7 as the cause of the limited antiproliferative response to ML-SI1 in 22Rv1 cells.

TRPML1 is primarily localized to late endosomes and lysosomes and has established roles in lysosomal function and autophagy (9, 10, 28). Given this cellular localization, lysosome-associated processes may contribute to the changes in AR stability observed following TRPML1 inhibition. However, the present experiments did not directly examine AR trafficking to lysosomes or determine whether inhibition of lysosomal degradation prevents ML-SI1-induced AR loss. Therefore, a lysosomal mechanism remains hypothetical and cannot be established based on the present data. Further experiments examining the lysosomal localization of AR and the effects of lysosomal inhibitors will be required to determine whether TRPML1 regulates AR turnover through a lysosome-associated pathway.

Genetic depletion of TRPML1 exerted inhibitory effects on LNCaP cell viability and 22Rv1 cell migration, and ML-SI1 did not induce a further reduction in the corresponding TRPML1-deficient cells. These results support a TRPML1-dependent component of the cellular effects of ML-SI1. However, because AR abundance and turnover were not directly examined in TRPML1-deficient cells, the present data do not establish that the effect of ML-SI1 on AR stability is itself TRPML1-dependent.

Polyamines, including putrescine, spermidine, and spermine, are small, positively charged molecules involved in DNA replication, transcription, protein function, cell proliferation, and differentiation (23, 29–31). Increased polyamine synthesis is a common feature of rapidly proliferating cells, and dysregulated expression of enzymes such as ODC1 and SMS has been associated with tumor growth (23, 24). Polyamine metabolism is particularly active in the prostate, where spermine is present at relatively high levels and contributes to normal prostate physiology (32). In contrast, spermine levels are lower in malignant prostate tissue than in benign prostate tissue, and reduced urinary spermine levels have also been reported in patients with PCa (33, 34). These observations indicate that alterations in polyamine and spermine metabolism are associated with prostate malignancy. Spermine can influence prostate cancer biology through several processes, including the regulation of cellular metabolism, oxidative stress, apoptosis, and antitumor immune responses (34, 35). Smith et al. reported that spermine inhibited prostate carcinoma cell growth *in vitro* and *in vivo*, although the response varied among tumor models with different metastatic potential (36). More recently, Li et al. demonstrated that spermine suppressed AR-FL and AR-V7 signaling in castration-resistant prostate cancer through the inhibition of PRMT1 and reduced CRPC growth *in vivo* (37). These findings show that the biological effects of spermine in prostate cancer can vary depending on the cellular and disease context.

In the present study, ML-SI1 induced distinct changes in polyamine metabolism in LNCaP and 22Rv1 cells. In LNCaP cells, ML-SI1 reduced ODC1 and SMS protein expression and decreased intracellular and extracellular spermine levels. In contrast, 22Rv1 cells exhibited increased spermine levels at 24 h, accompanied by increased ODC1 and SMS expression. These opposing responses indicate that the effects of ML-SI1 on polyamine metabolism differ between the two cell models. The molecular basis underlying this difference remains unclear and may be related to differences in the molecular backgrounds of the two cell models, including their dependence on AR signaling. In 22Rv1 cells, the increase in spermine levels occurred simultaneously with reduced migration and invasion. Considering the previously reported inhibitory effects of spermine on prostate cancer and AR signaling, one possible hypothesis is that increased spermine levels contribute to the reduction in migration and invasion following ML-SI1 treatment. However, this relationship was not directly examined in the present study. Spermine levels or ODC1/SMS activity were not manipulated to determine whether alterations in the polyamine pathway modify the effects of ML-SI1 on cell migration or invasion. Therefore, the increase in spermine levels should currently be regarded as an associated metabolic change rather than a demonstrated mechanism. Further experiments involving the direct manipulation of spermine metabolism will be required to test this hypothesis.

Accordingly, the findings support a role for TRPML1 in regulating selected cellular phenotypes in prostate cancer cells and demonstrate that ML-SI1 induces distinct responses in LNCaP and 22Rv1 cells. In LNCaP cells, ML-SI1 reduced AR protein stability and decreased spermine levels, whereas in 22Rv1 cells, the primary phenotypic effects included reduced migration and invasion, accompanied by increased spermine levels at 24 h. These observations provide a basis for further investigation of the relationships among TRPML1, AR regulation, and polyamine metabolism in prostate cancer.

Several limitations should be considered. Although TRPML1 is a lysosomal ion channel, the present experiments do not directly demonstrate lysosome-mediated AR degradation, and the pathway responsible for the nonproteasomal component of AR turnover remains unresolved. The relationship between altered spermine metabolism and changes in cell migration has not been experimentally established. In addition, all functional experiments were conducted using cultured prostate cancer cell lines, and the effects and safety of ML-SI1 have not been evaluated *in vivo*. Further mechanistic studies and validation using appropriate tumor models will therefore be required to determine the biological and potential therapeutic relevance of these findings.

## 5 Conclusions

The effects of TRPML1 inhibition differ between the two prostate cancer cell models examined in this study. In LNCaP cells, ML-SI1 inhibits proliferation and migration and is associated with reduced AR protein stability and decreased spermine levels. In contrast, in 22Rv1 cells, ML-SI1 inhibits migration and invasion without a clear effect on proliferation and is accompanied by increased spermine levels at 24 h. These *in vitro* findings indicate that TRPML1 inhibition differentially affects AR regulation and polyamine metabolism in LNCaP and 22Rv1 cells. Further mechanistic and *in vivo* studies are required to clarify the biological and therapeutic significance of these observations.

## Data Availability

The datasets presented in this study can be found in online repositories. The data generated and/or analyzed during the current study are available in the Figshare repository at https://doi.org/10.6084/m9.figshare.31910752. Publicly available datasets analyzed in this study were obtained from TCGA, HPA, and CCLE. Full-length original blot images are provided as an additional supplementary file entitled ‘Original Images for Blots’.
