# Supplementary material for: TRPML1 inhibitor ML-SI1 suppresses prostate cancer through dual regulation of androgen receptor and polyamine metabolism
Source: Front Med (Lausanne). 2026 Sep 28;13:1959805. doi: 10.3389/fmed.2026.1959805 (PMC13654739; doi:10.3389/fmed.2026.1959805)

Figure 2F   Group 1

All the Group 1 blots was used in the main Figureure

TRPML1

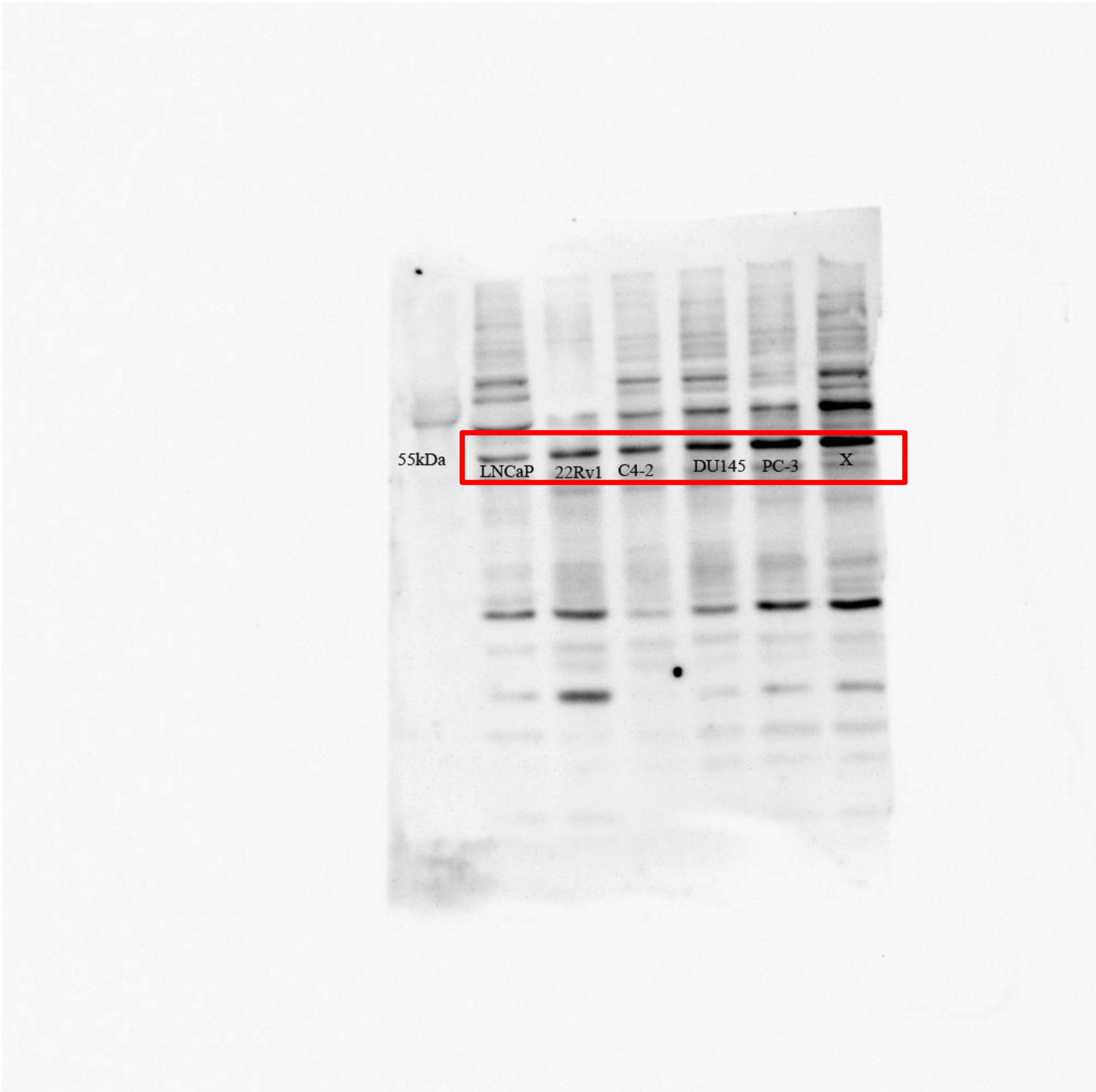

β -actin

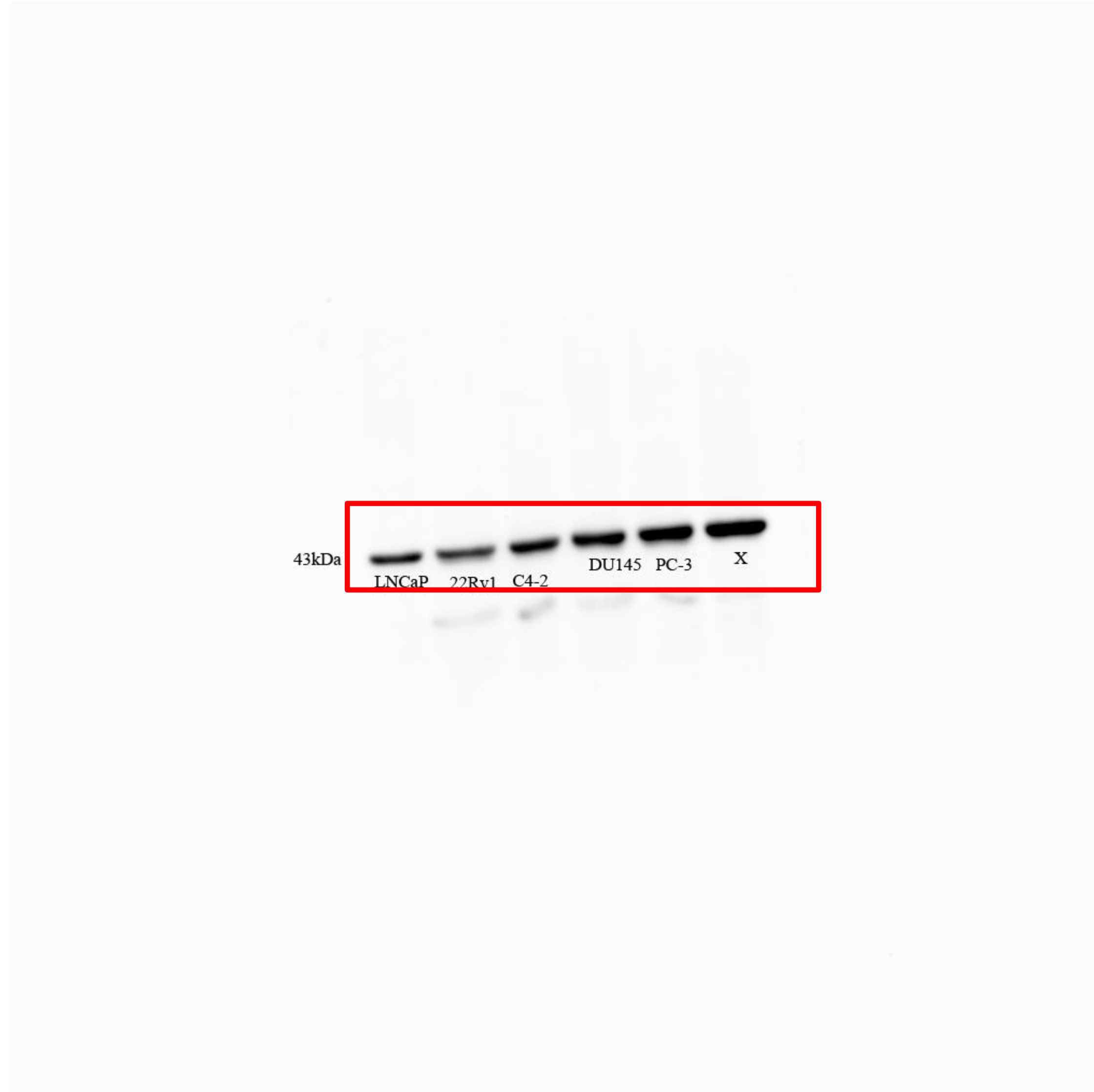

Figure 2F   Group 2

TRPML1

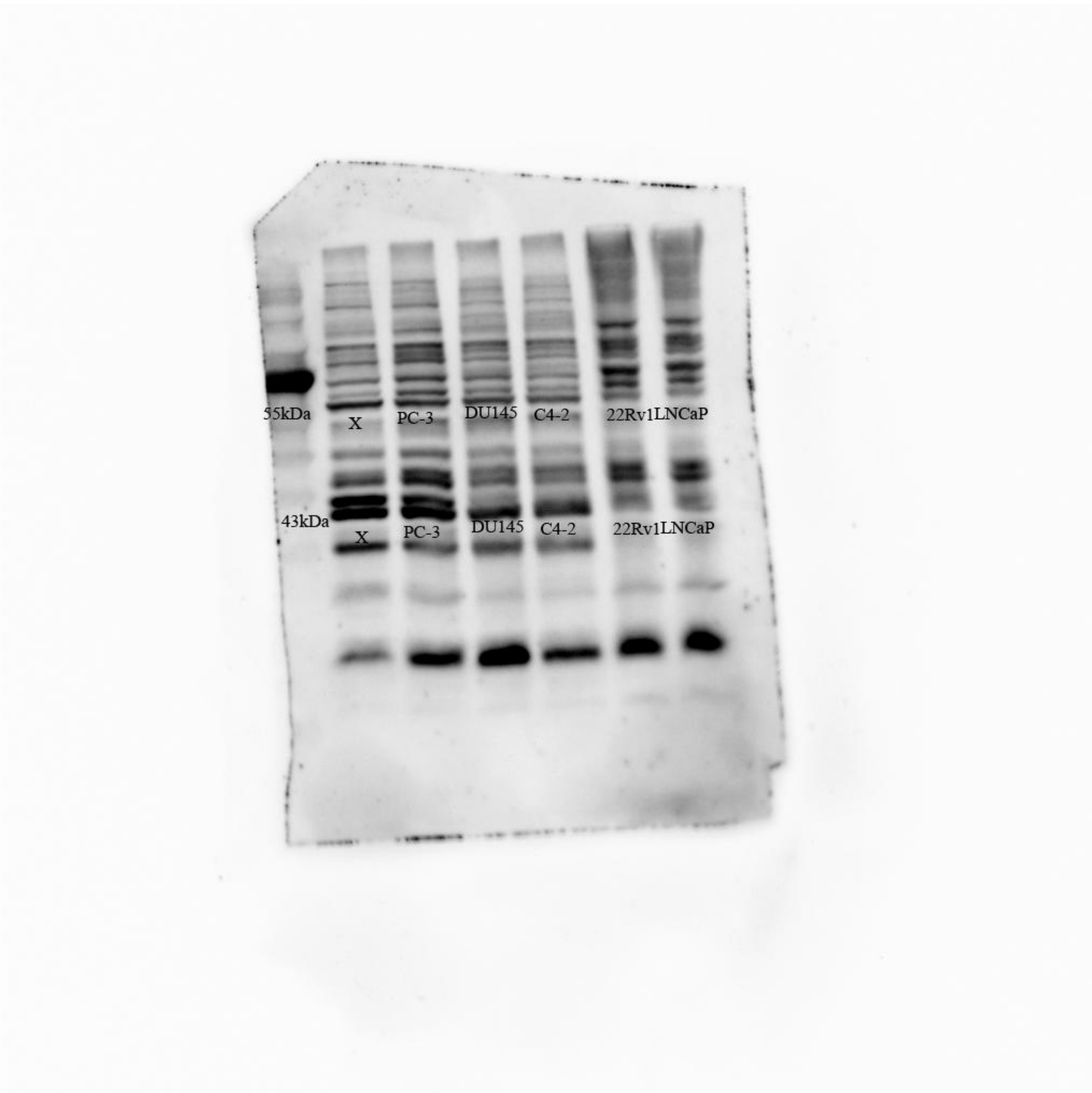

β -actin

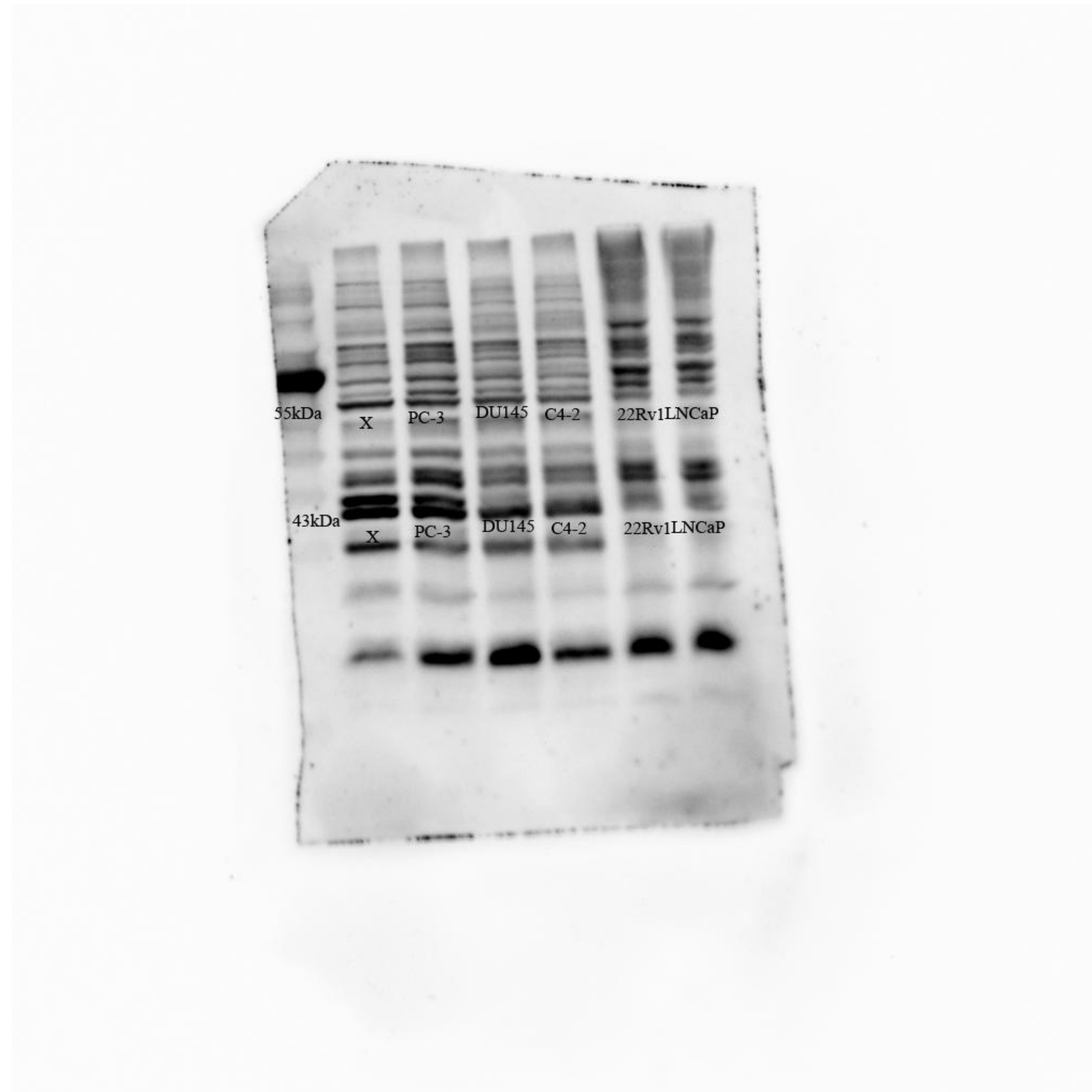

Figure 2F   Group 3

TRPML1

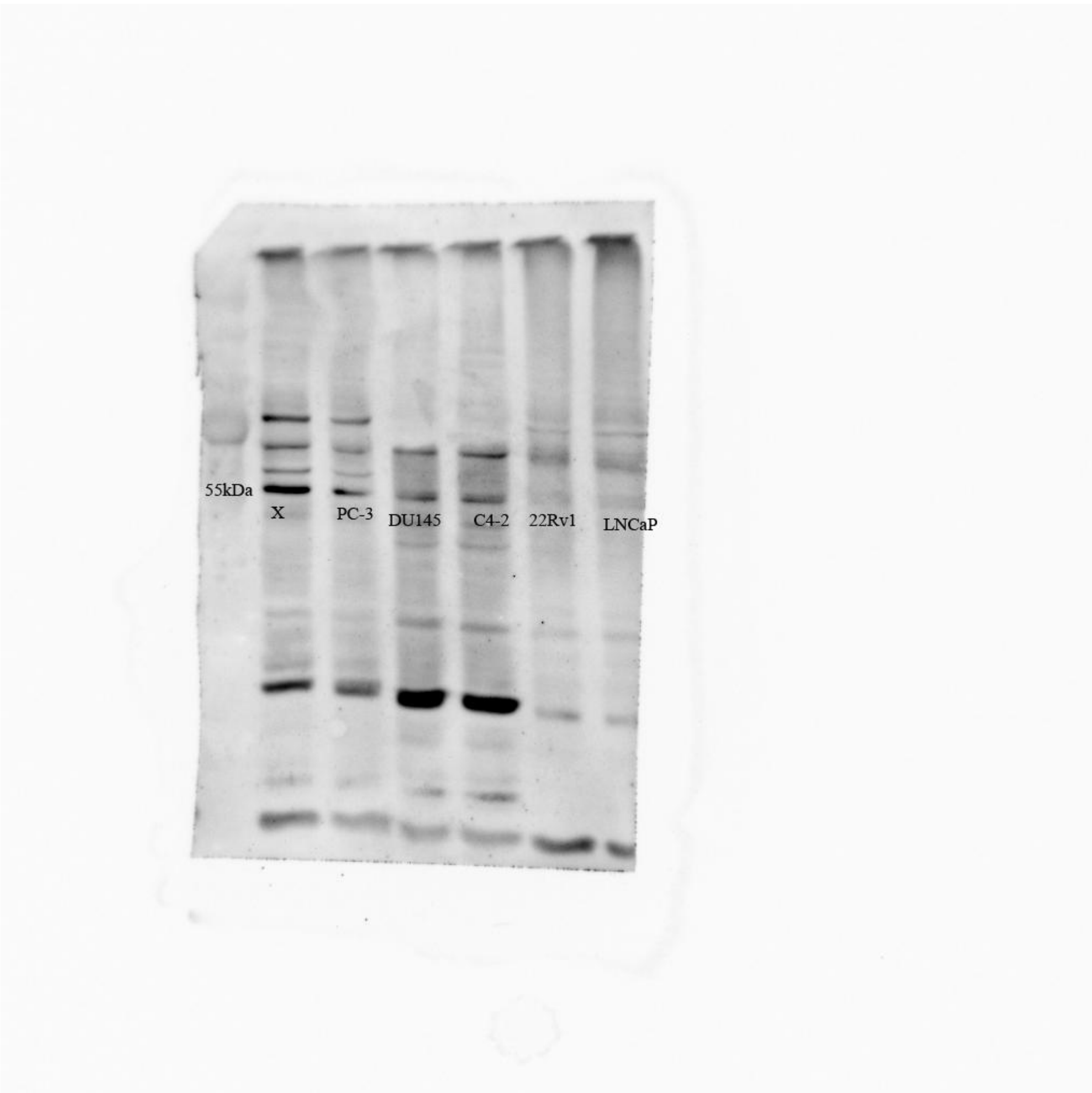

β -actin

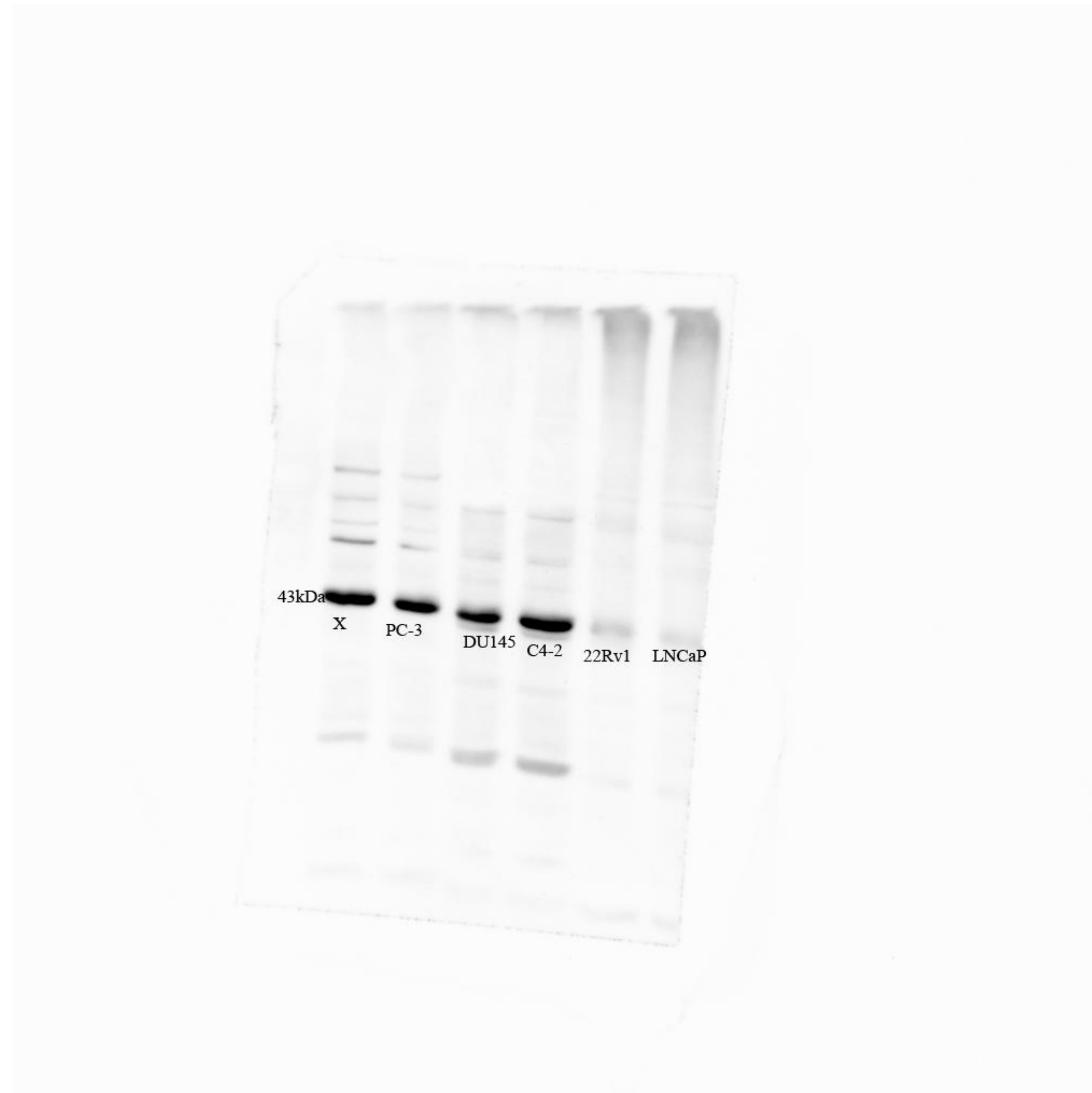

Figure 3F Group 1

TRPML1

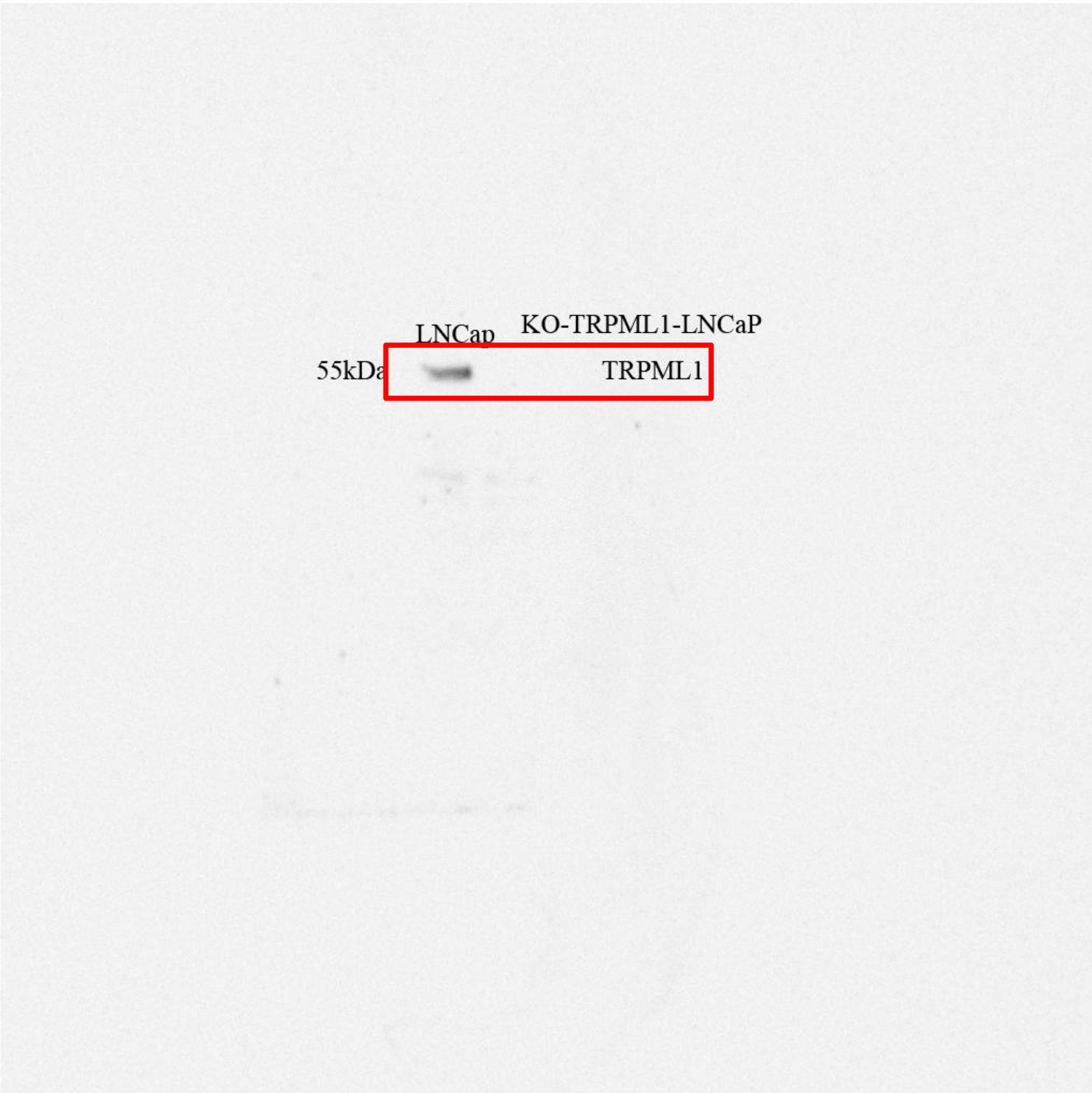

GAPDH

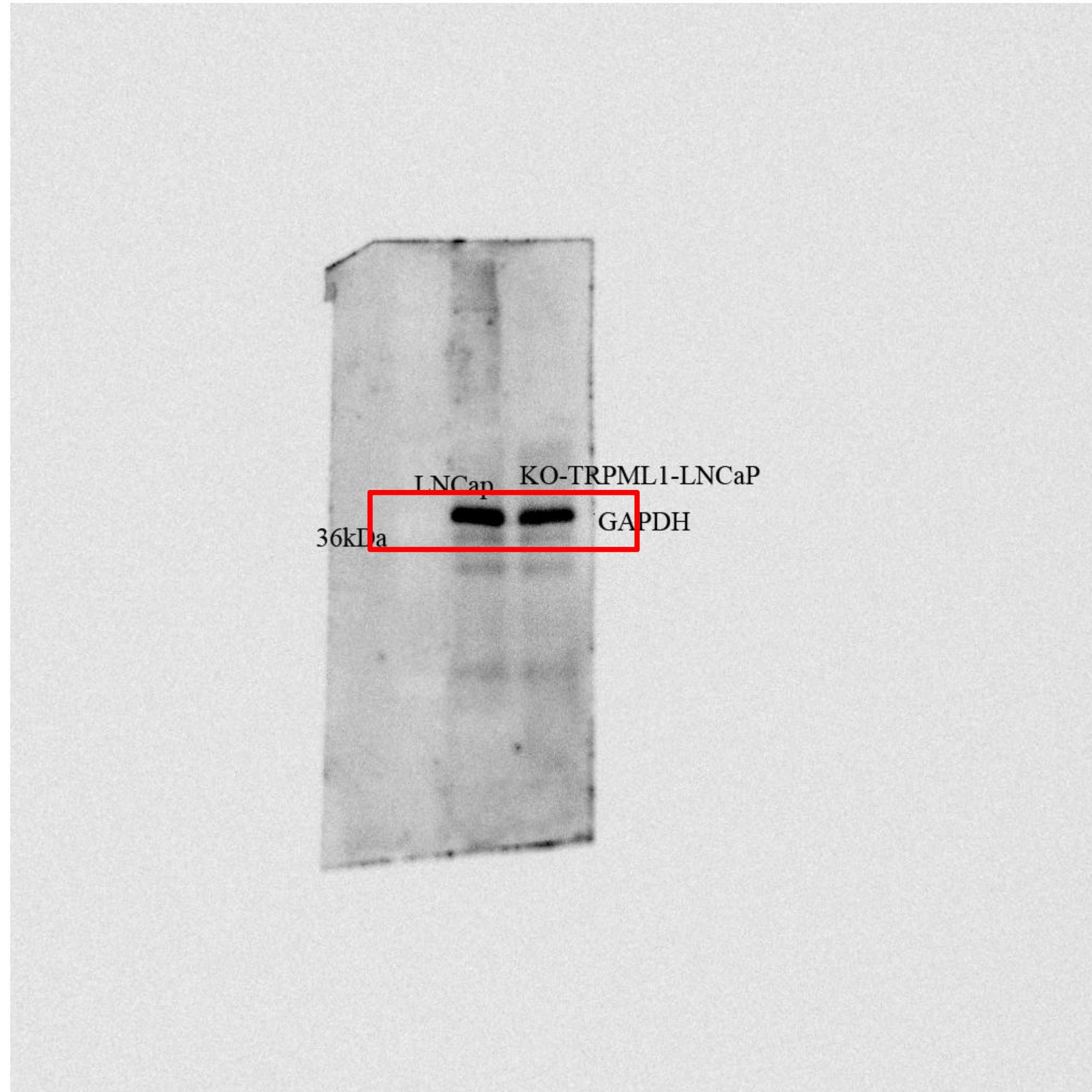

Figure 3F Group 2

TRPML1

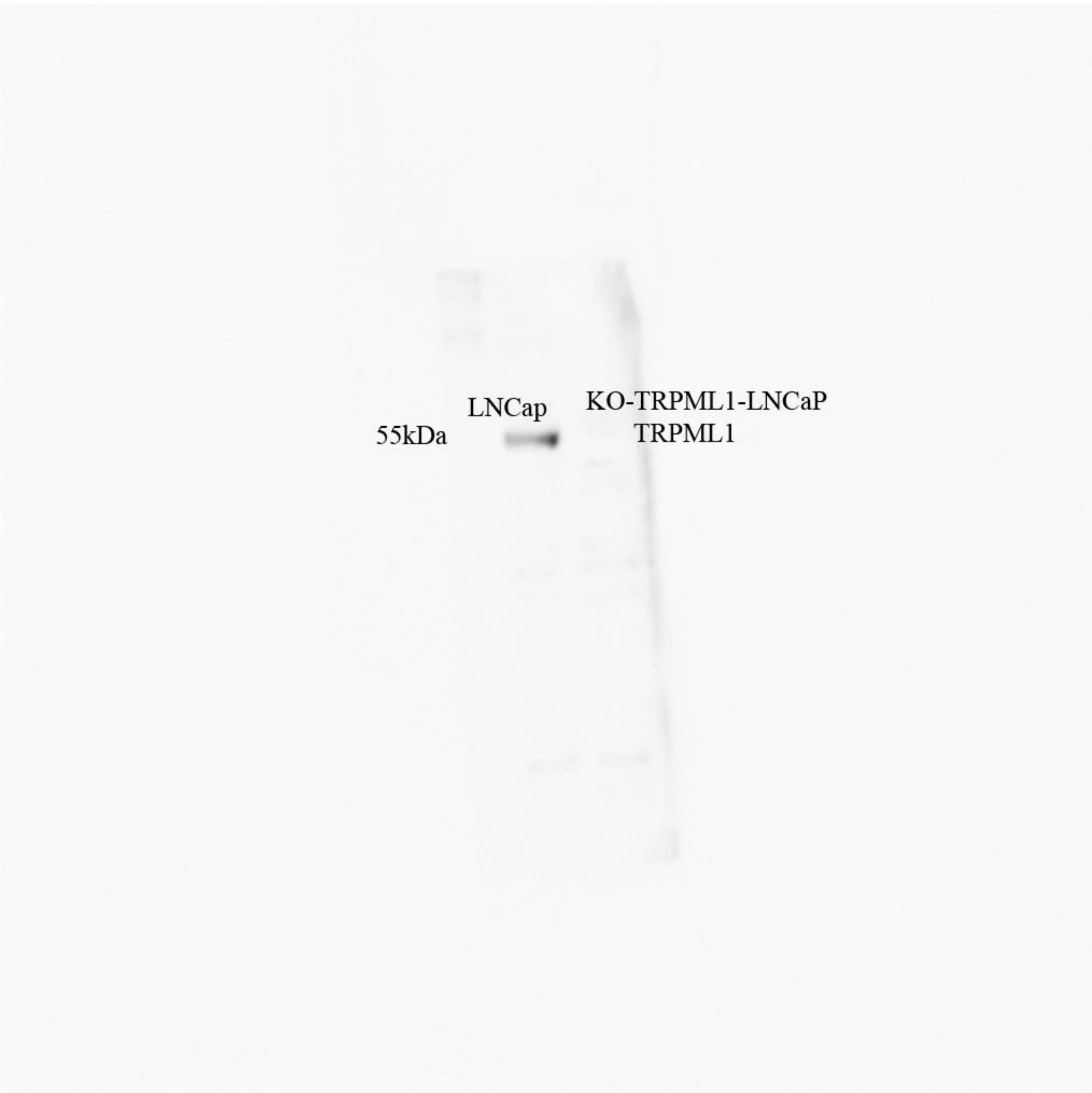

GAPDH

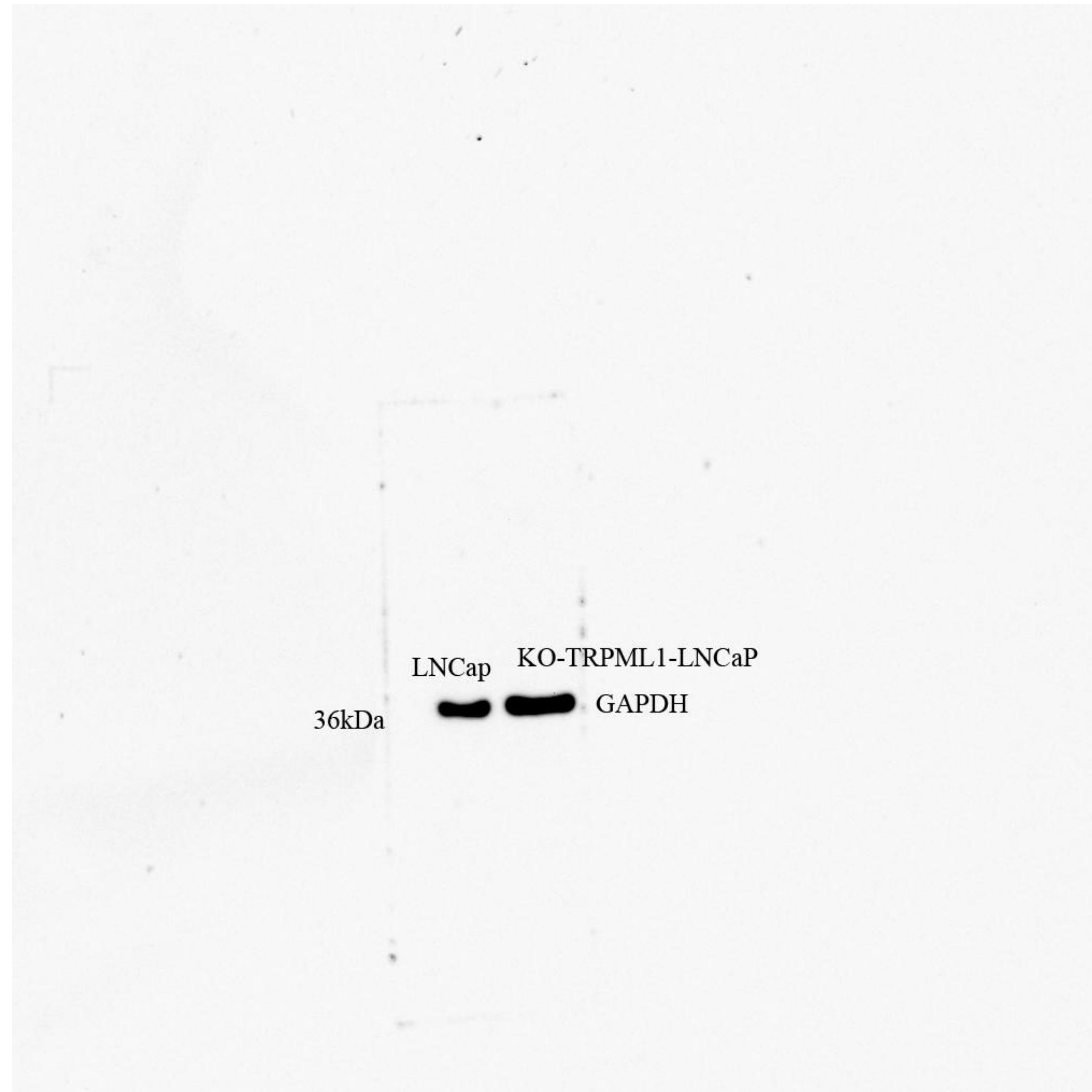

Figure 3F Group 3

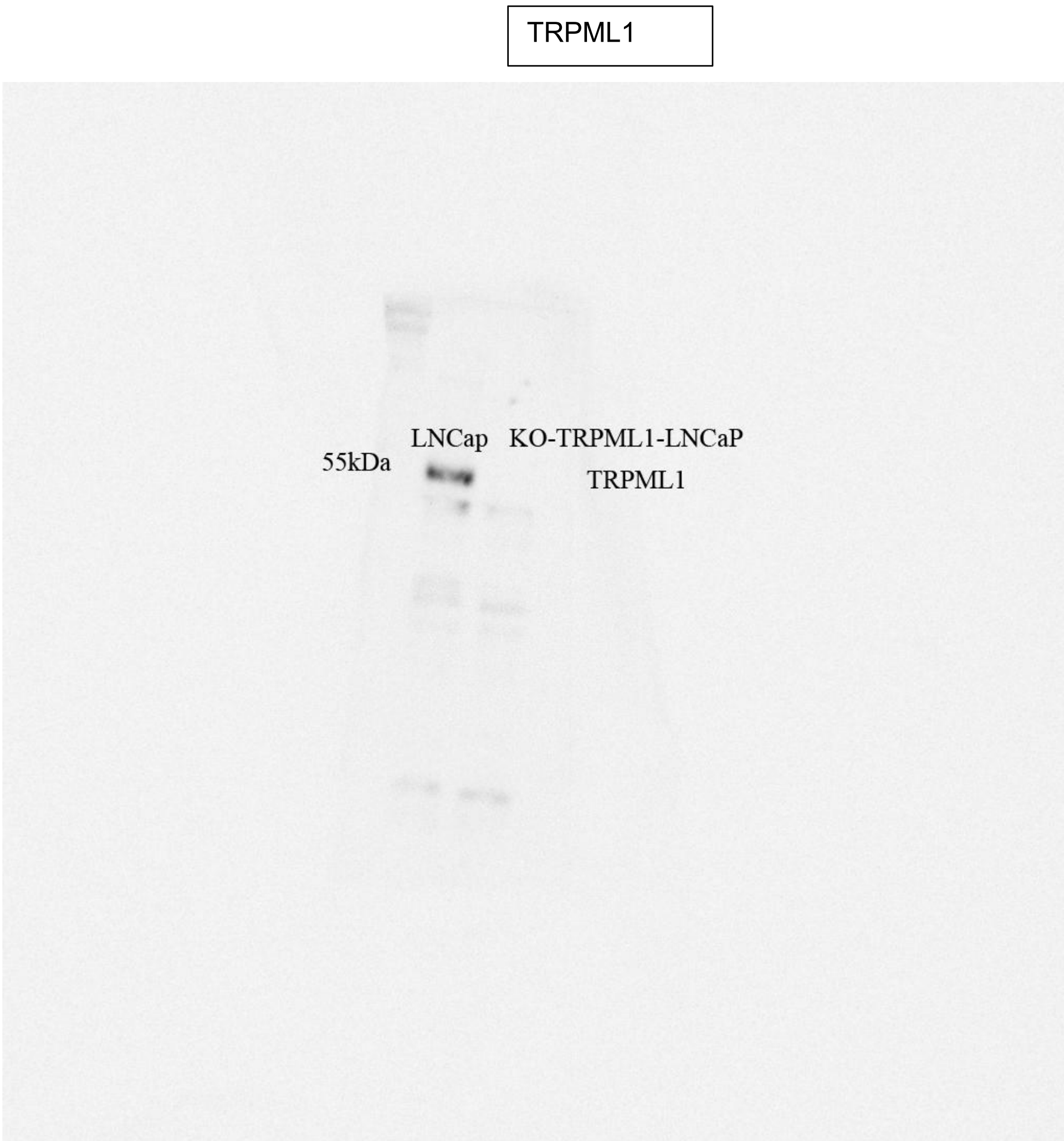

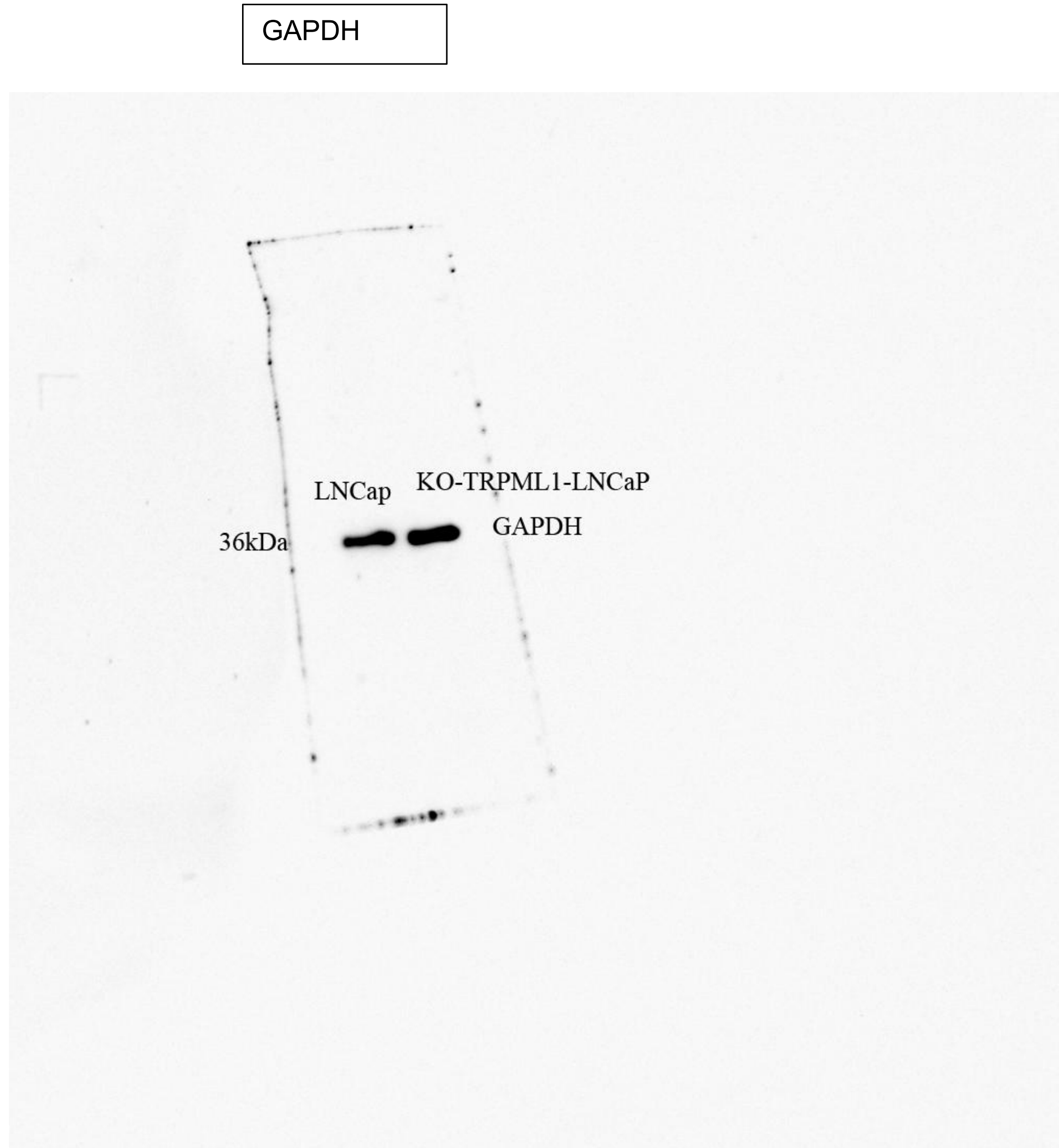

Figure 4H Group 1

TRPML1

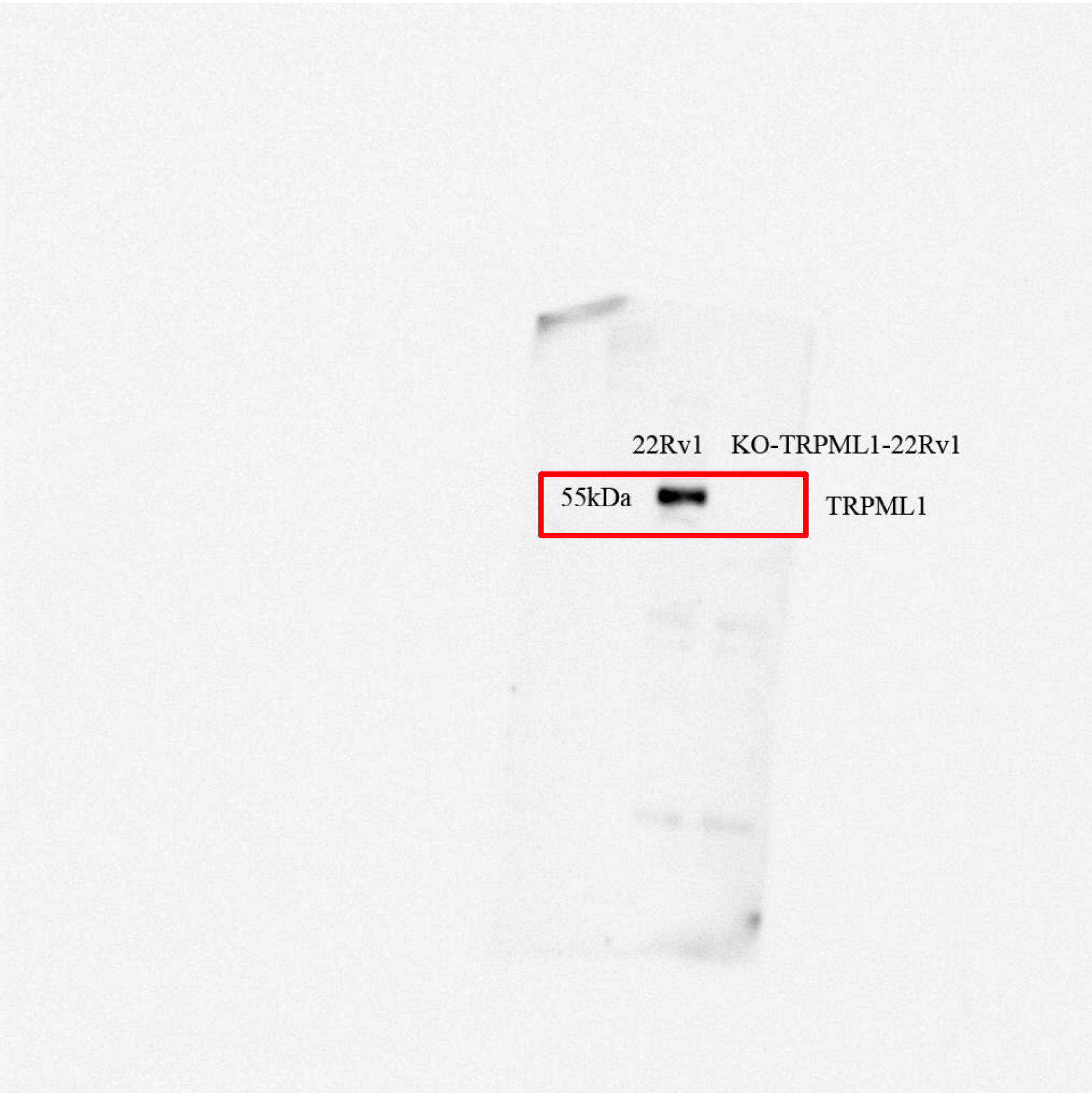

GAPDH

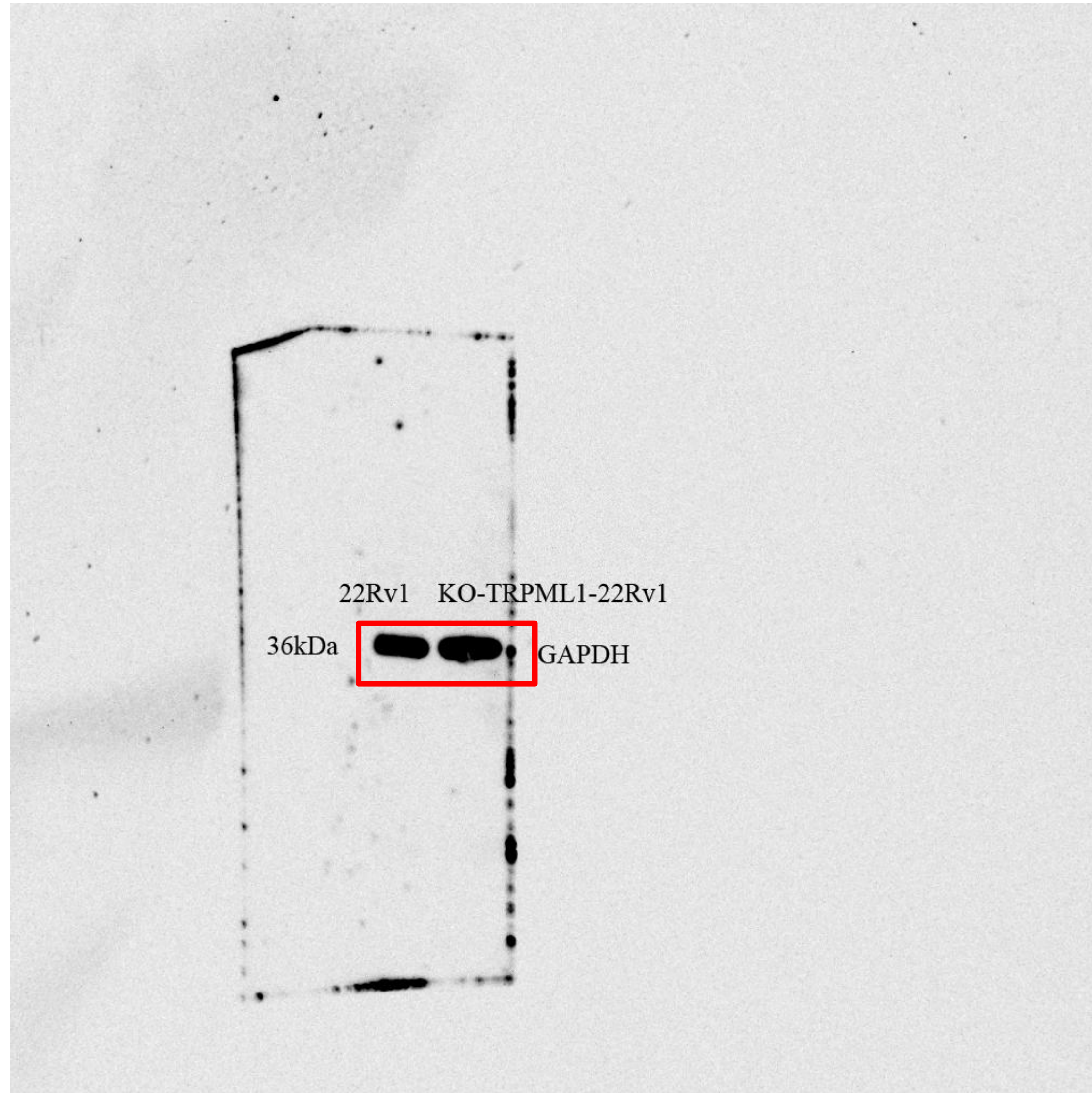

Figure 4H   Group 2

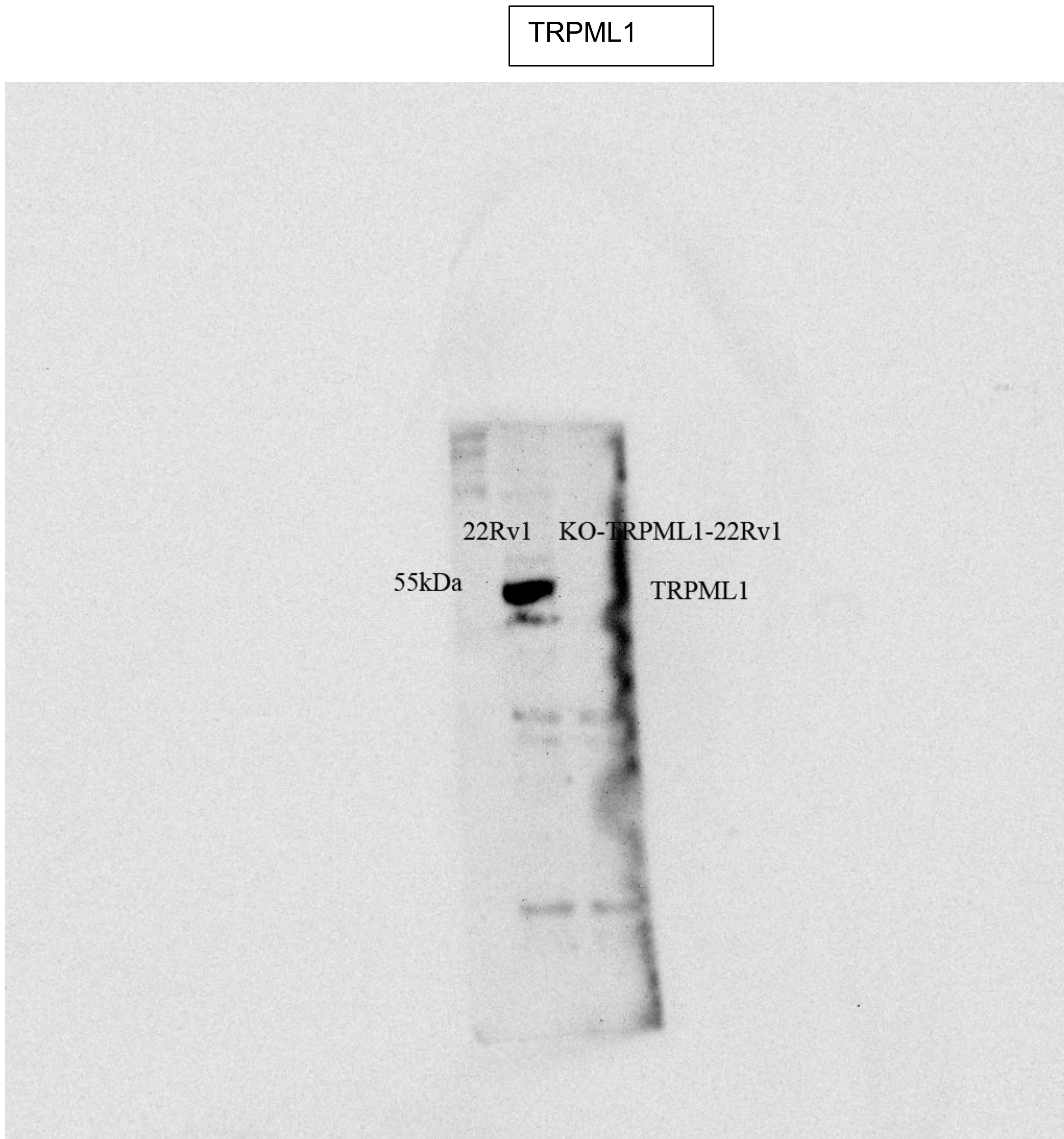

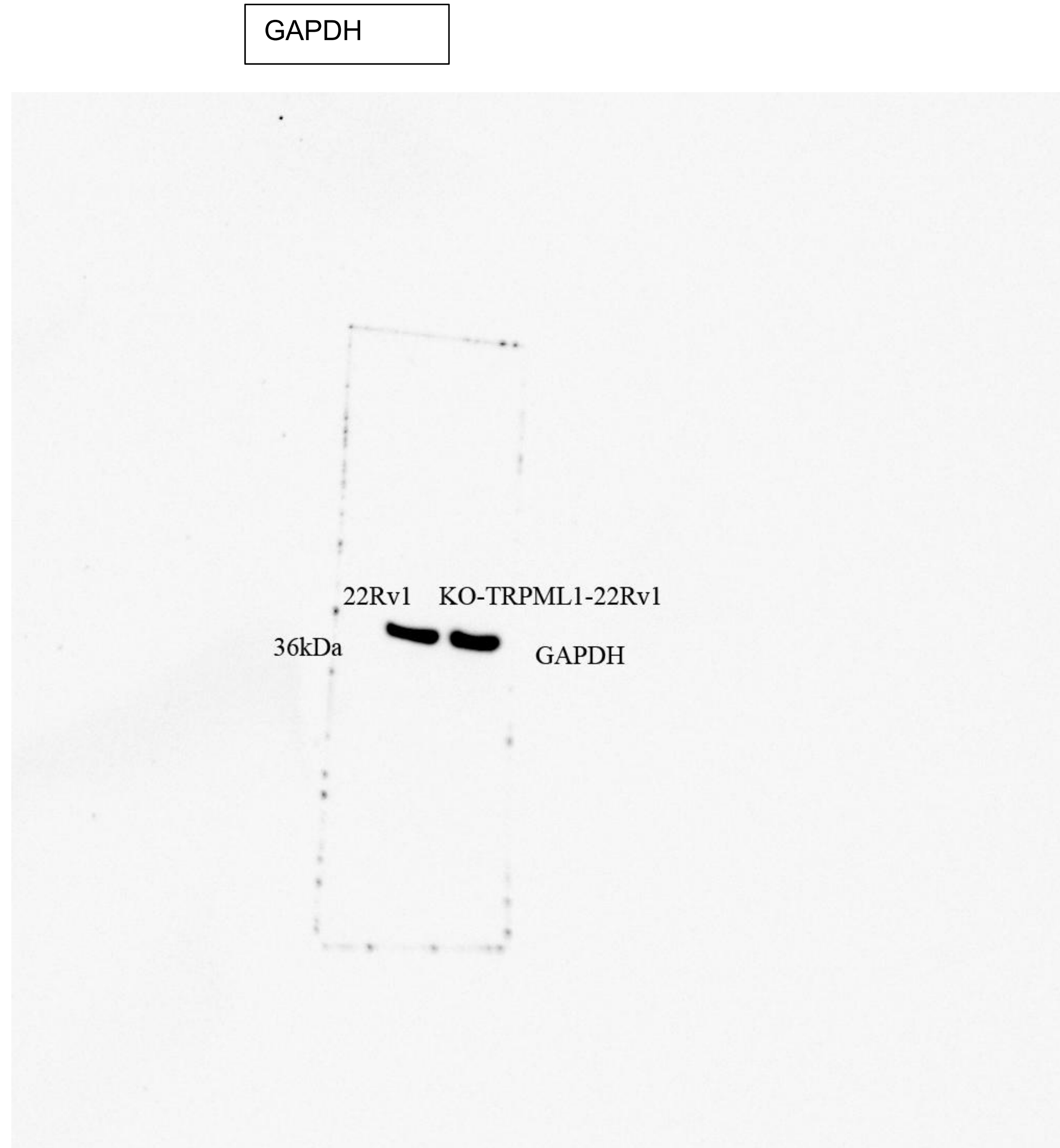

Figure 4H   Group 3

TRPML1

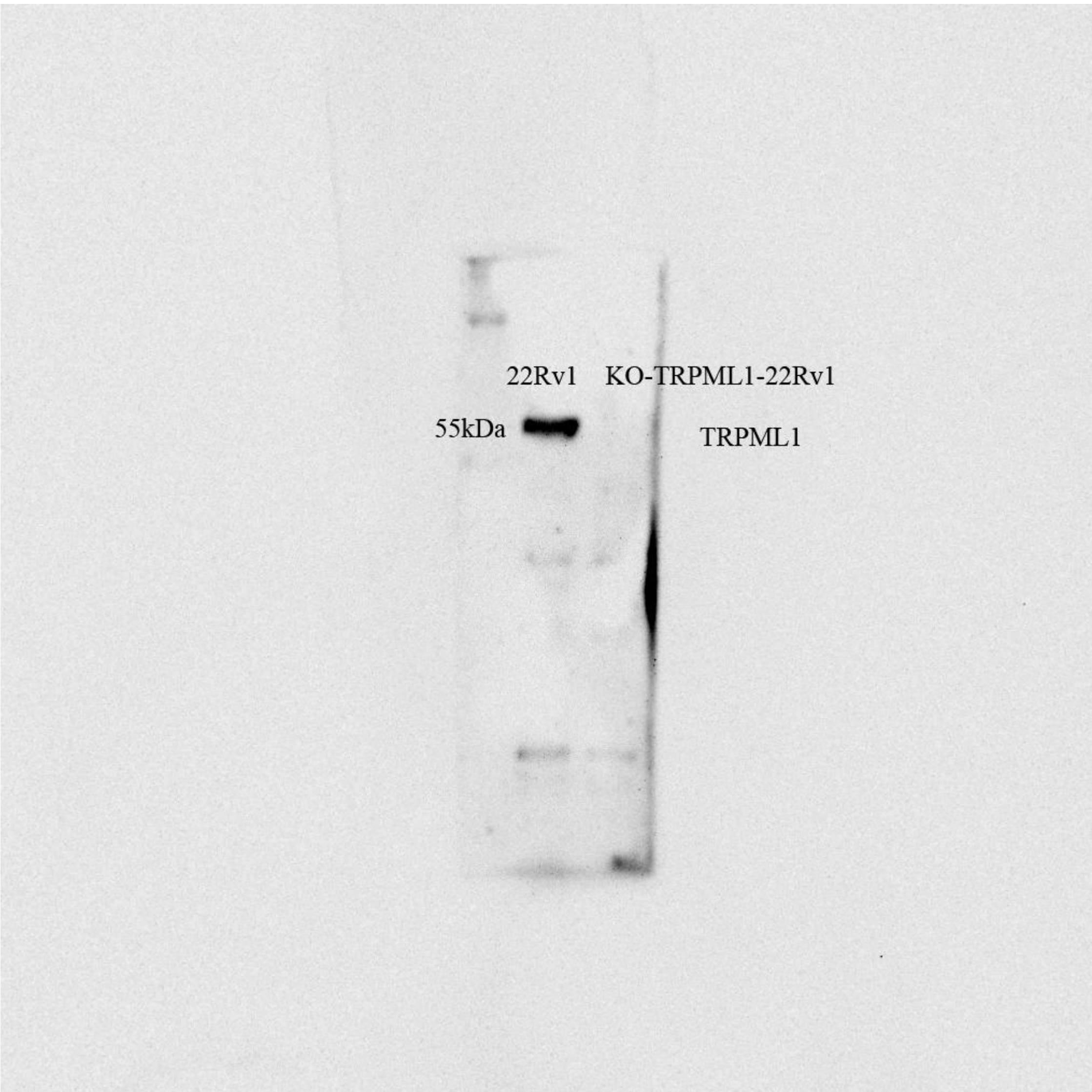

GAPDH

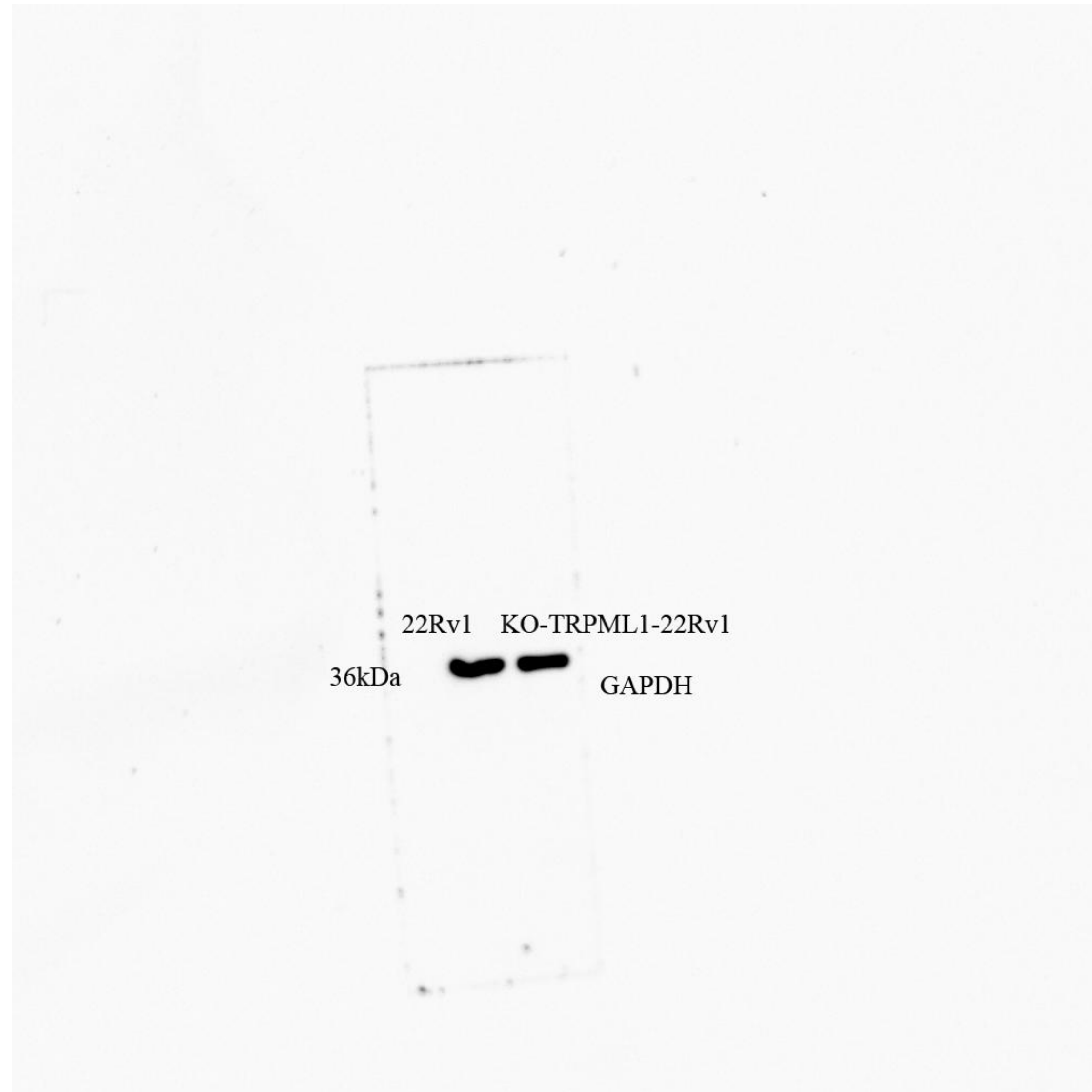

Figure 6B Group 1

AR

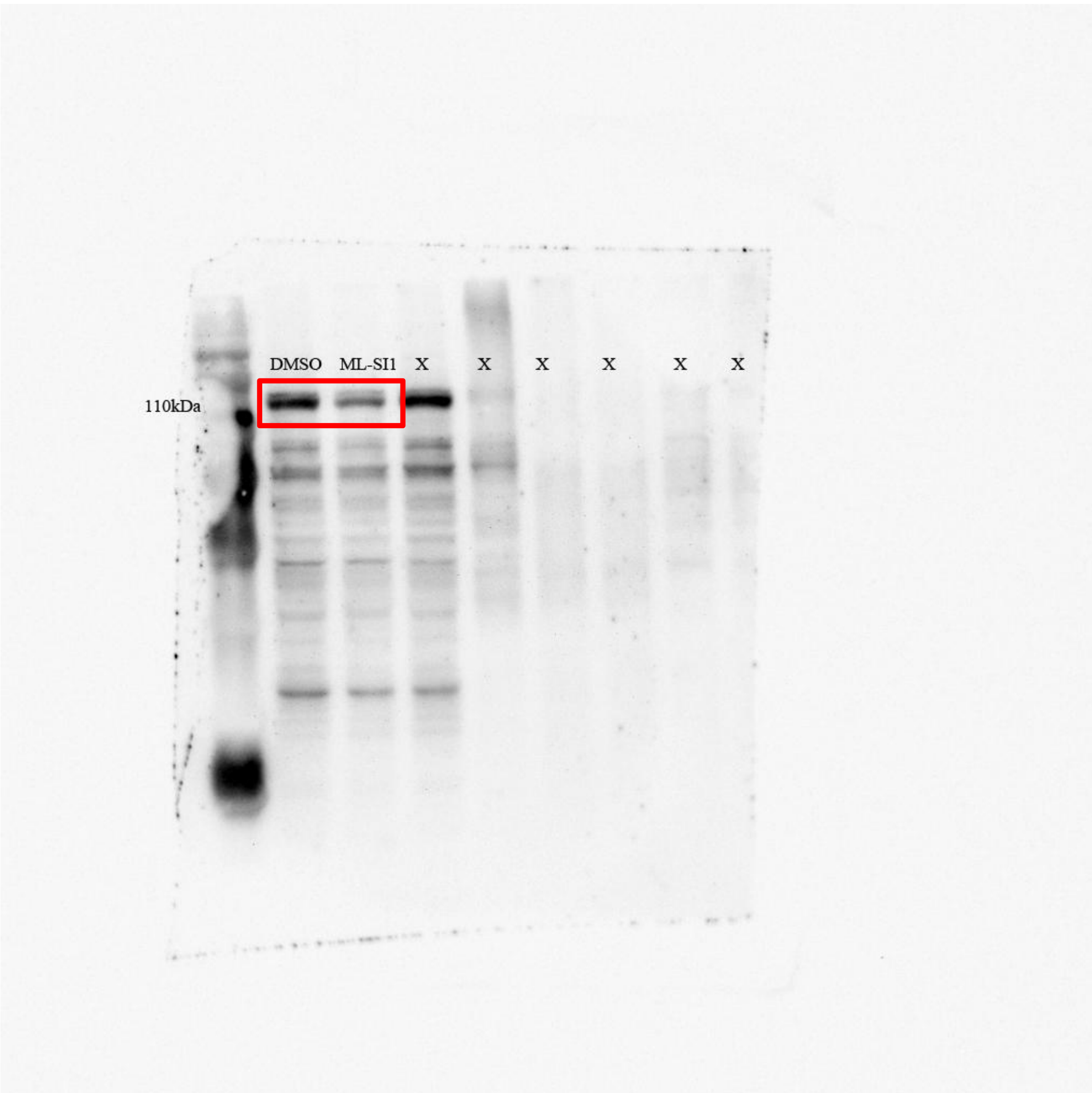

β -actin

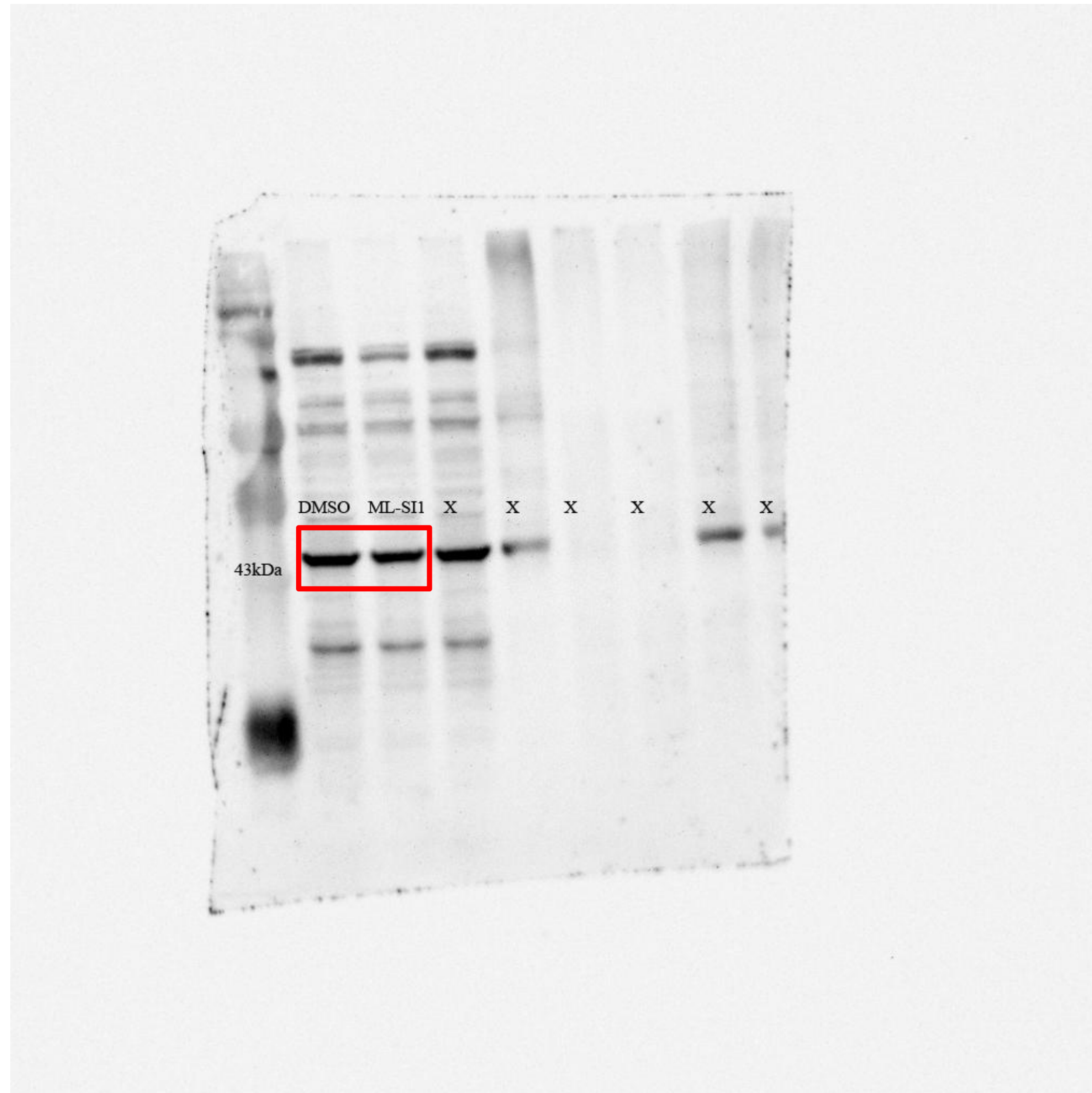

Figure 6B Group 2

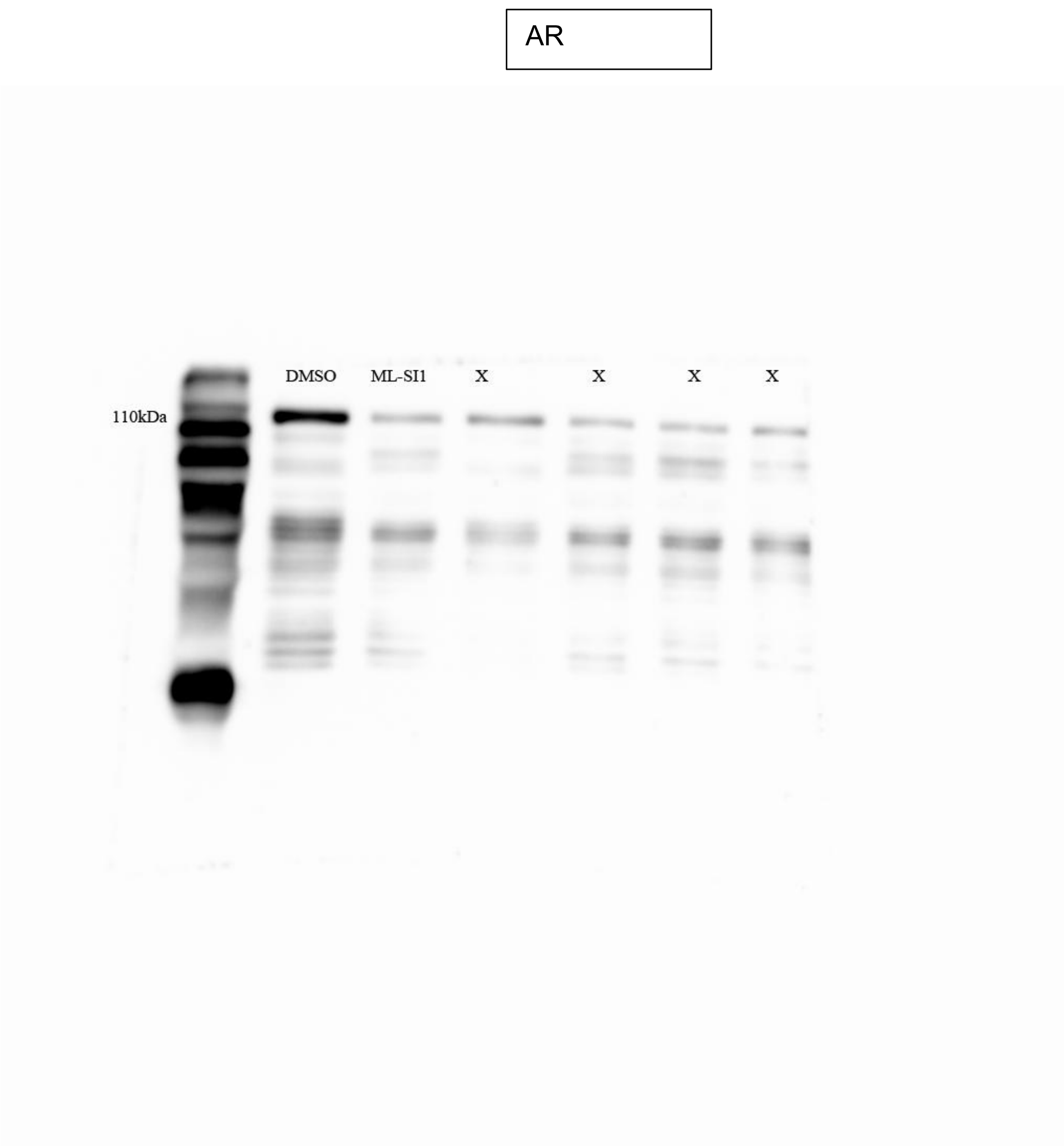

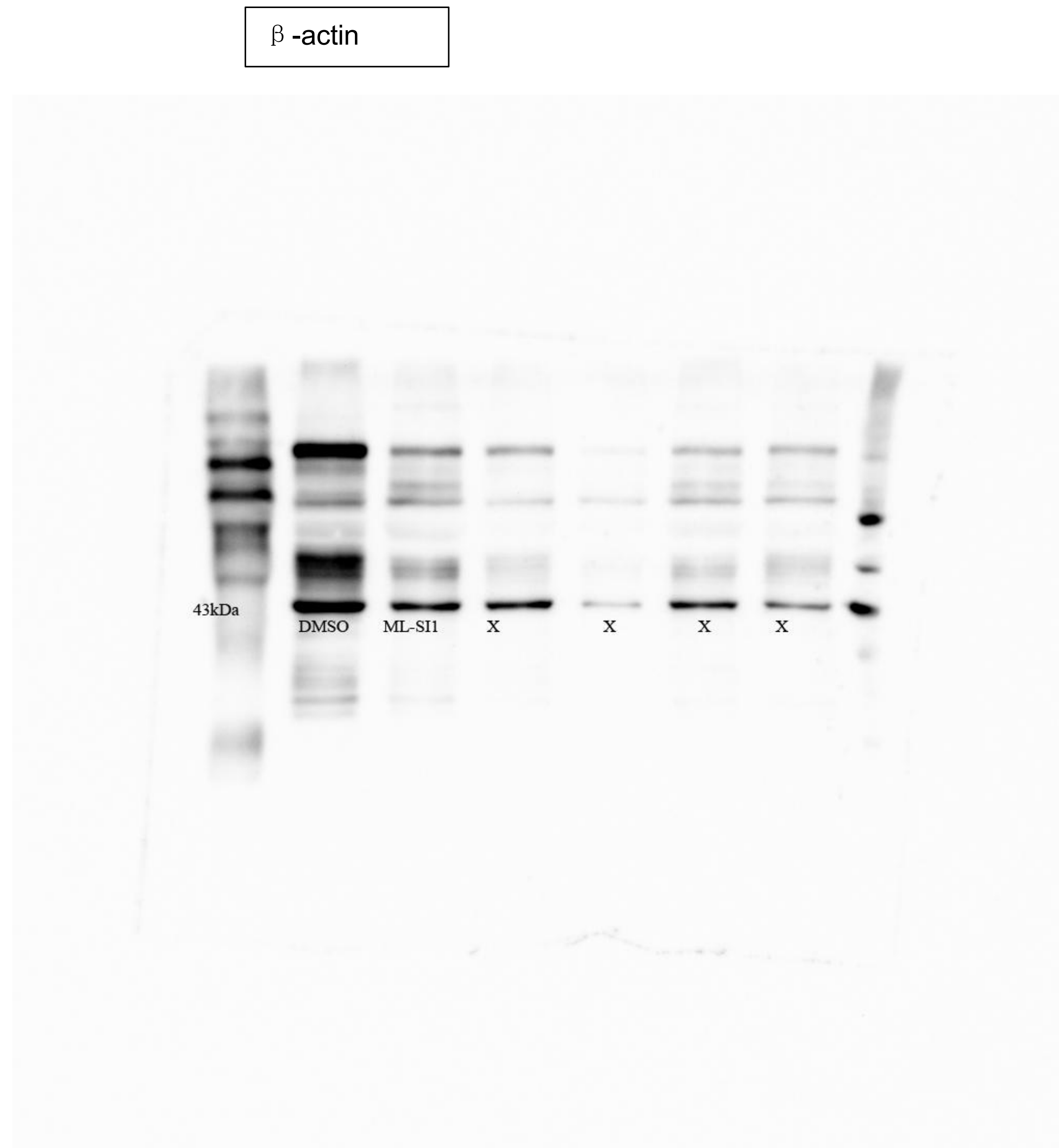

Figure 6B Group 3

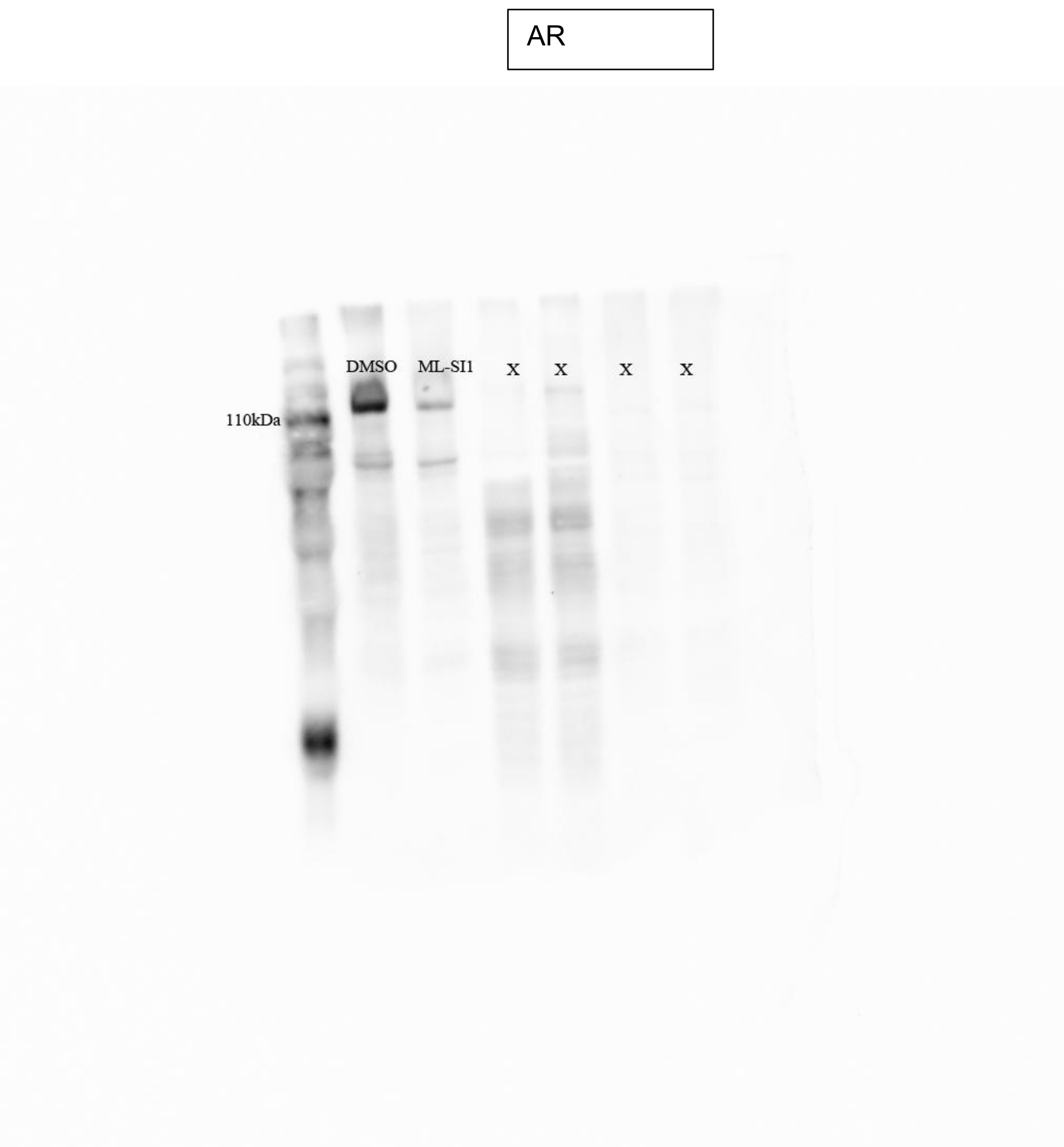

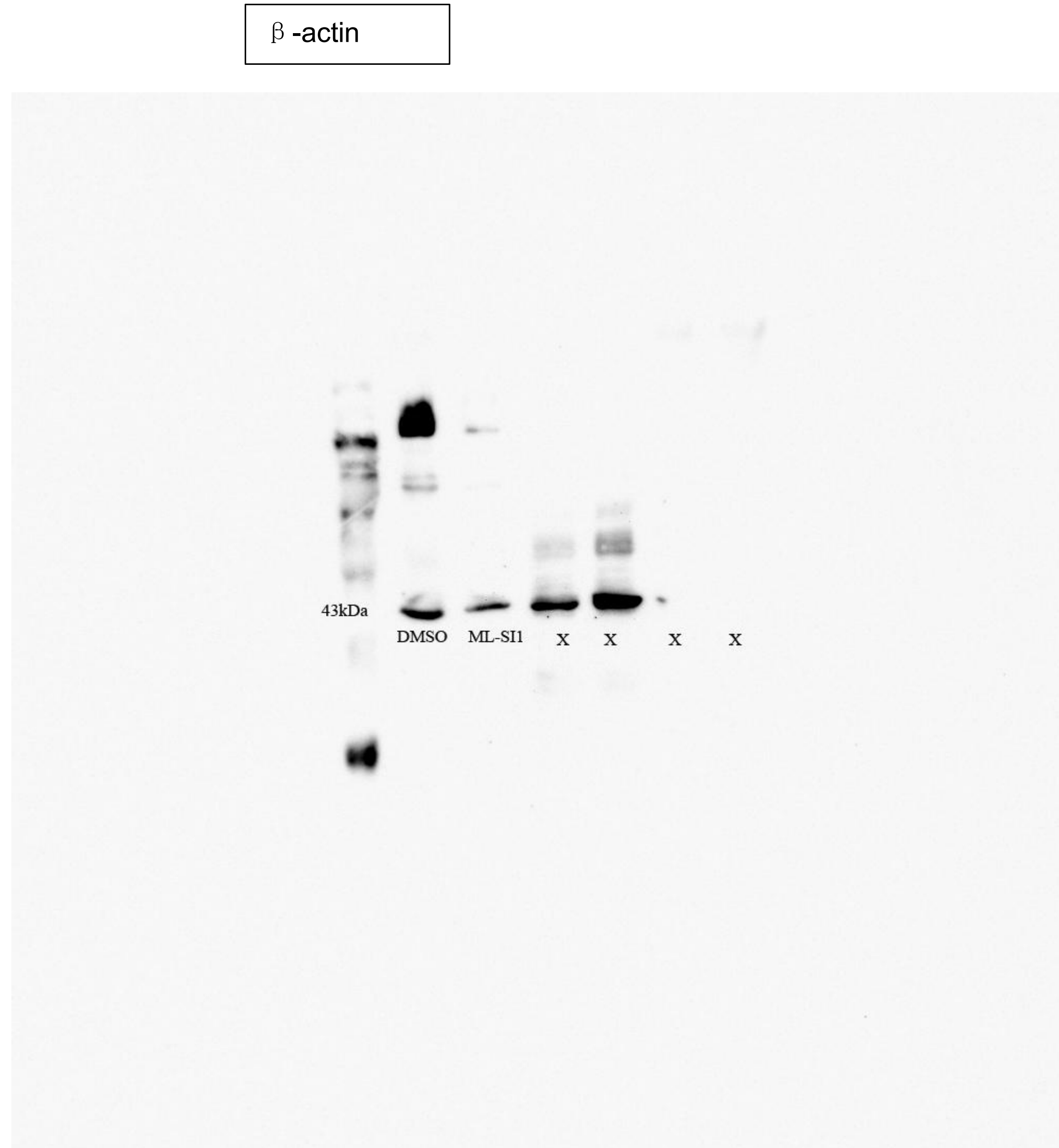

Figure 6D Group 1

AR

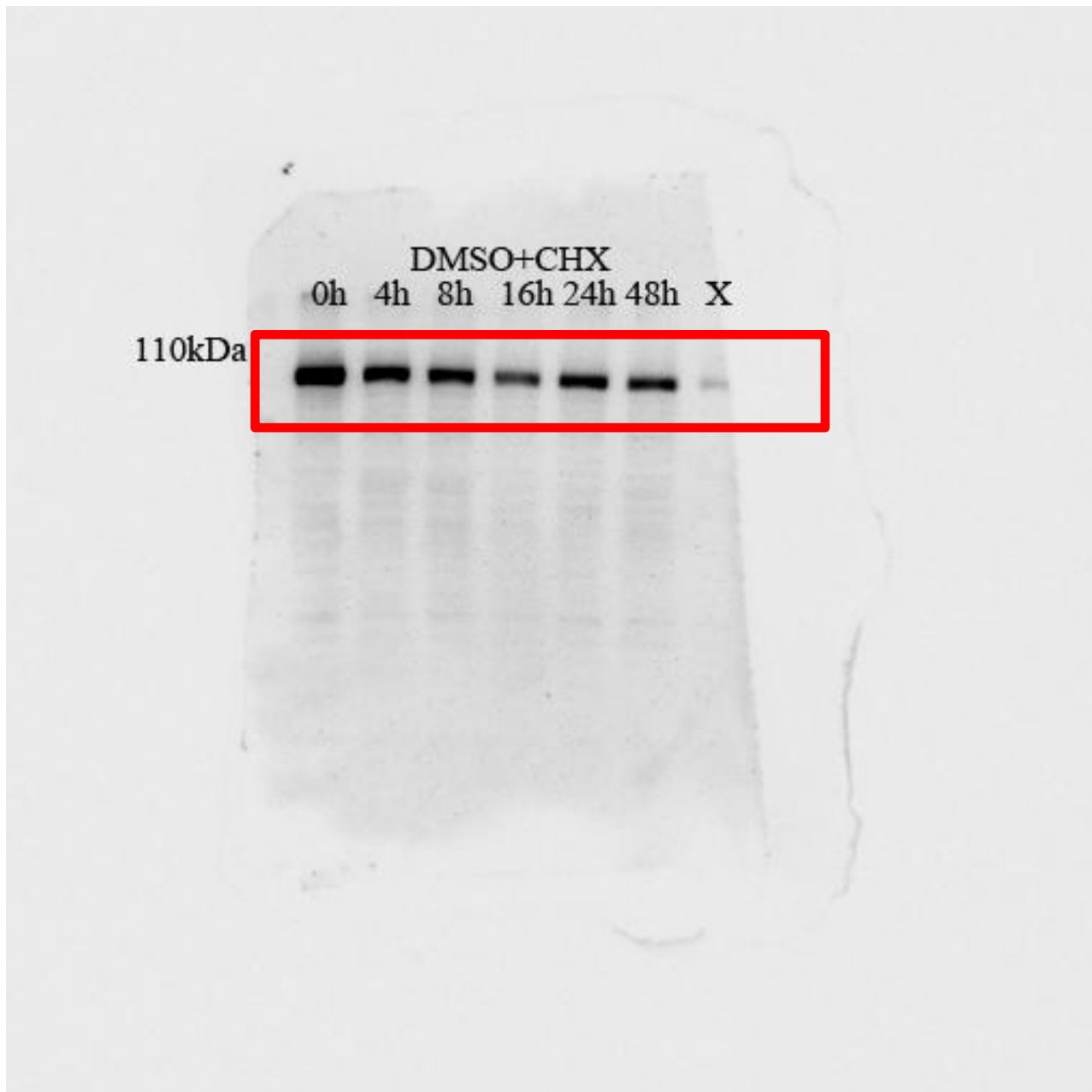

GAPDH

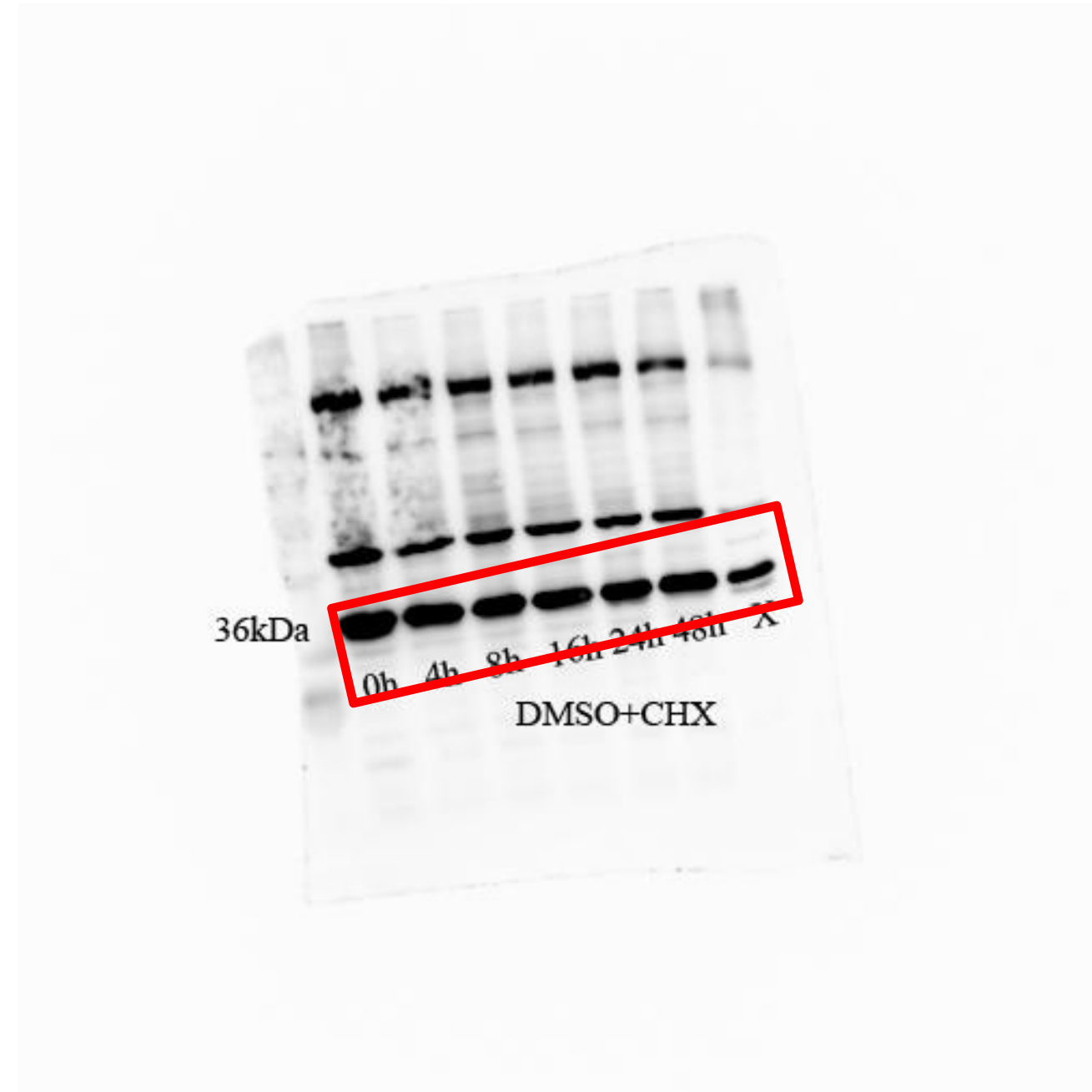

AR

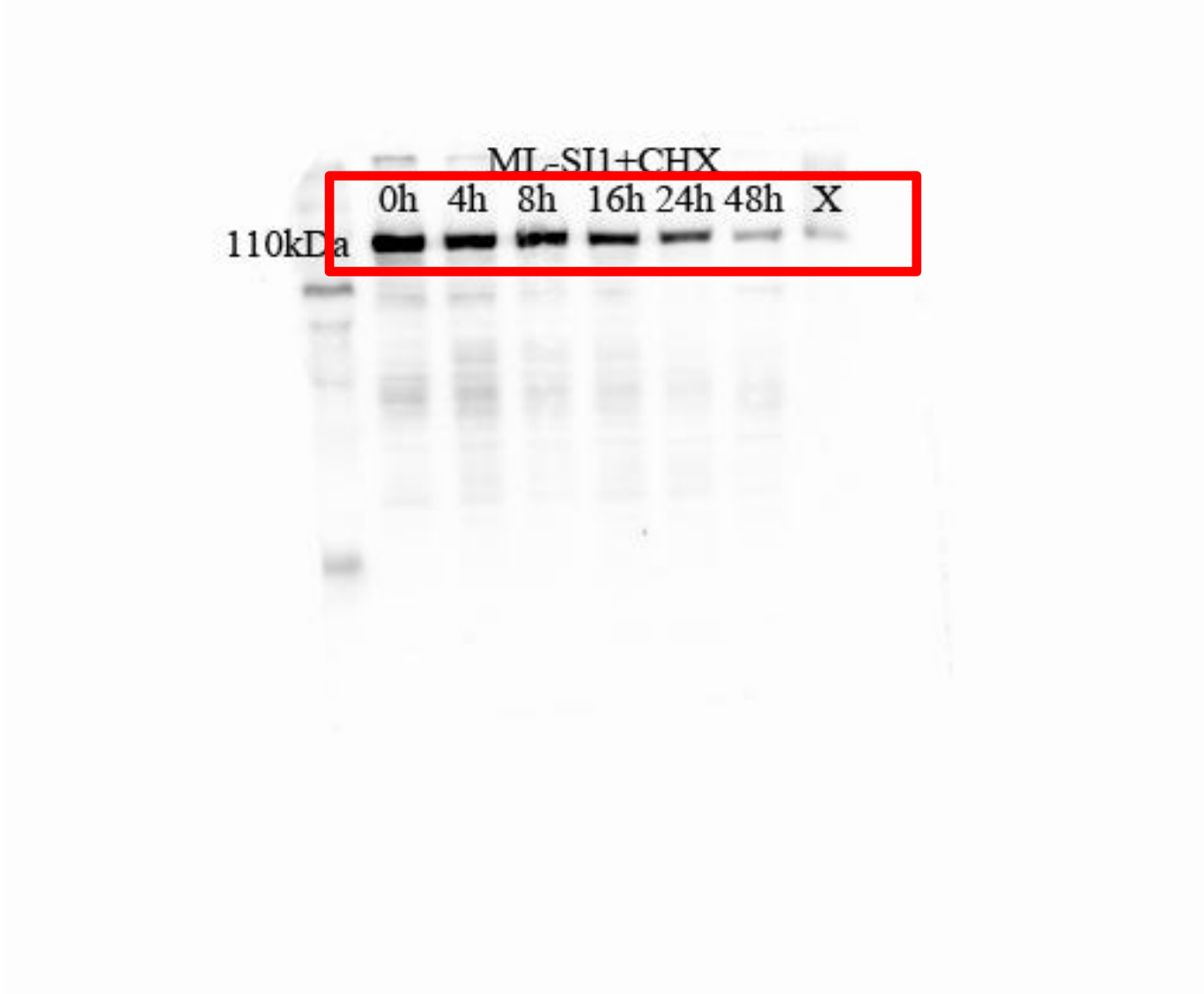

GAPDH

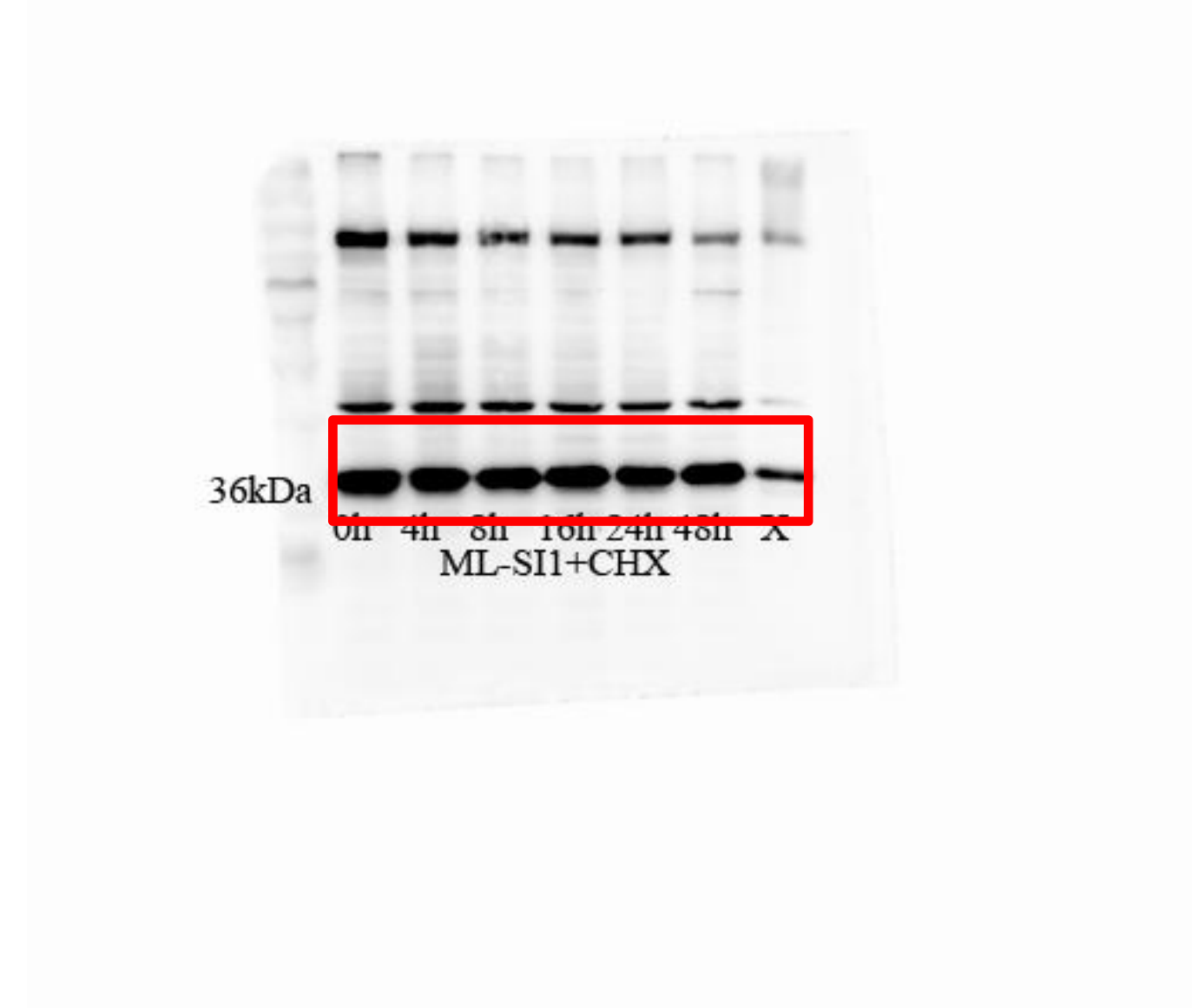

Figure 6D   Group 2

AR

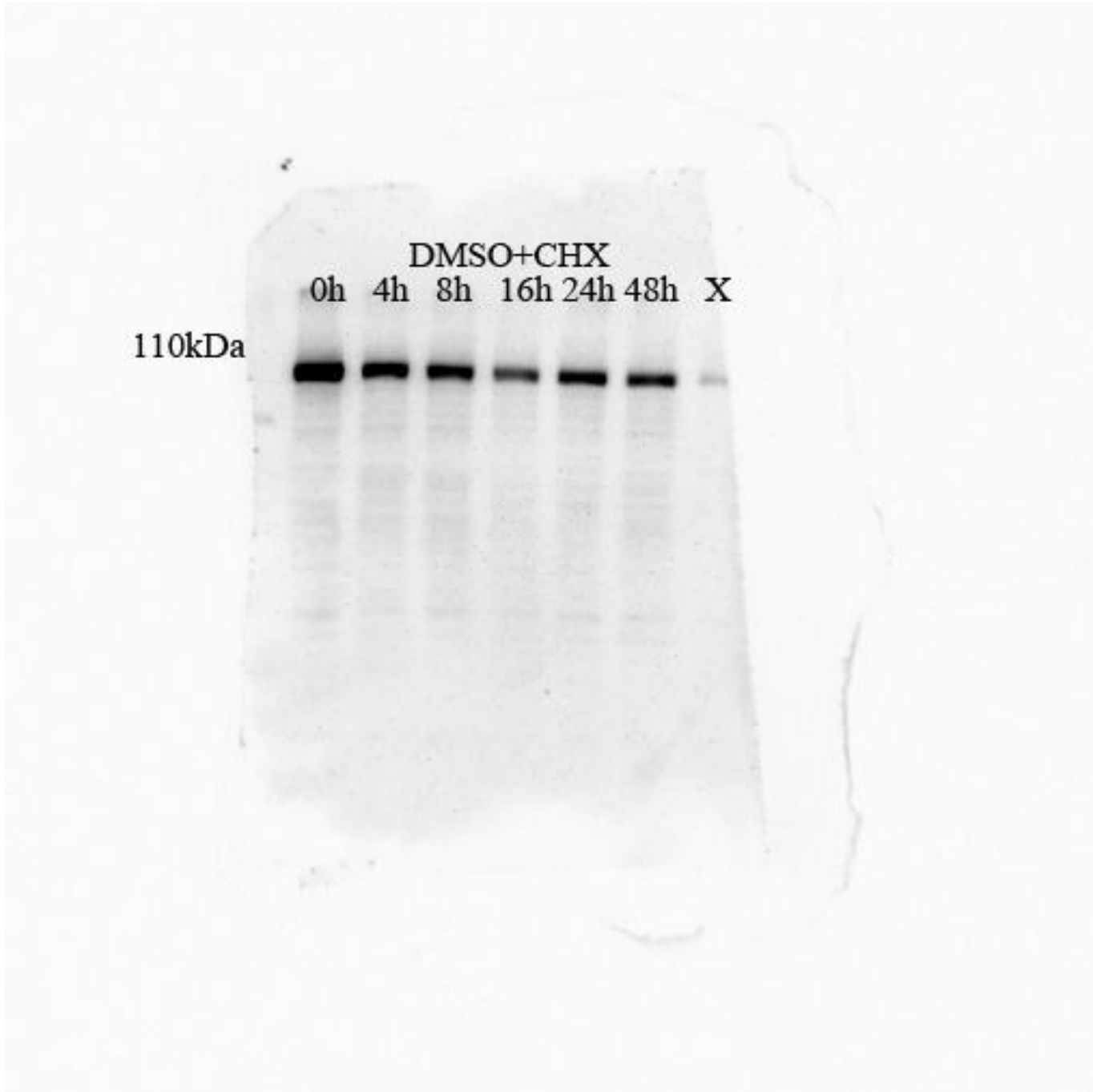

GAPDH

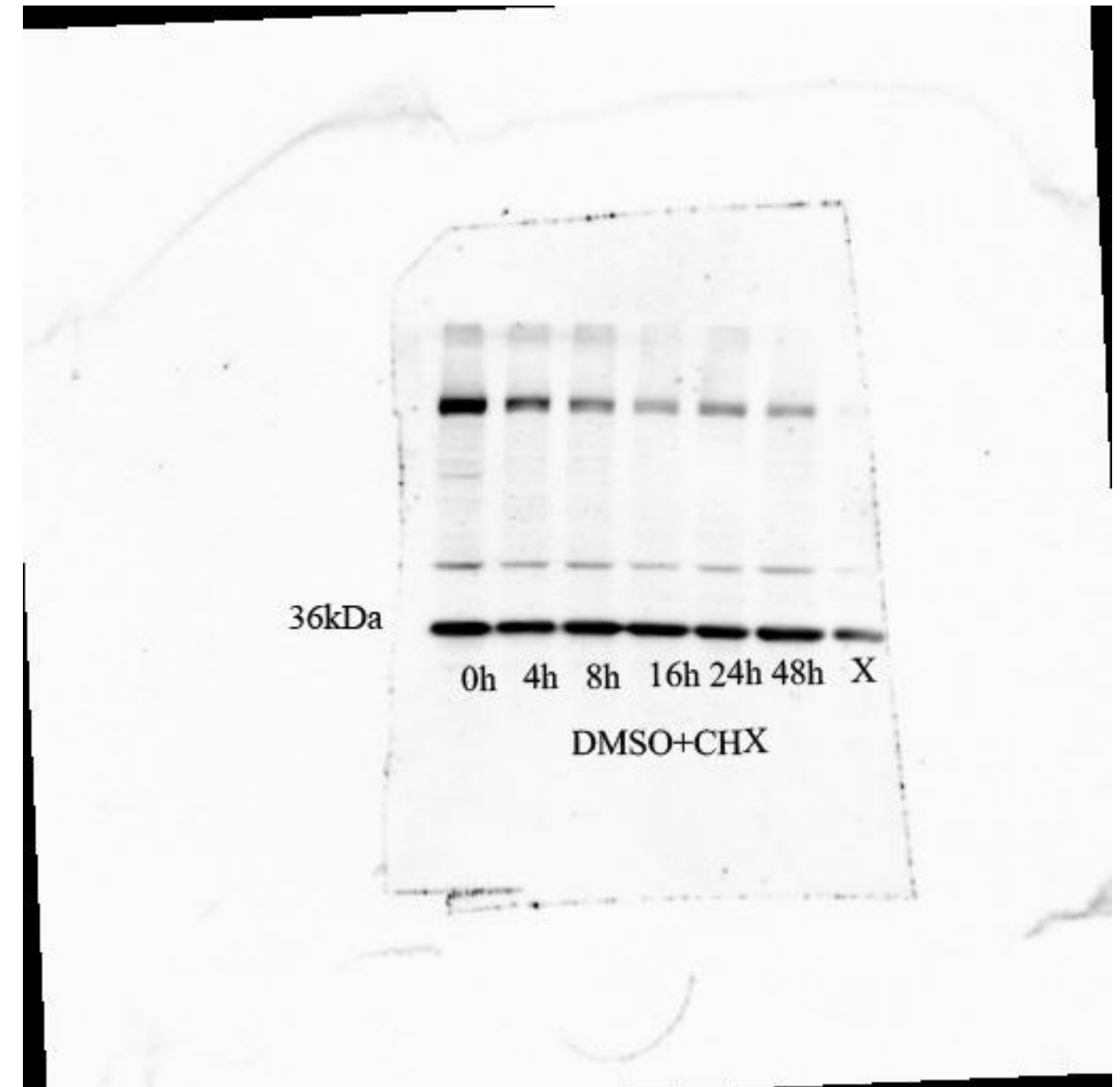

AR

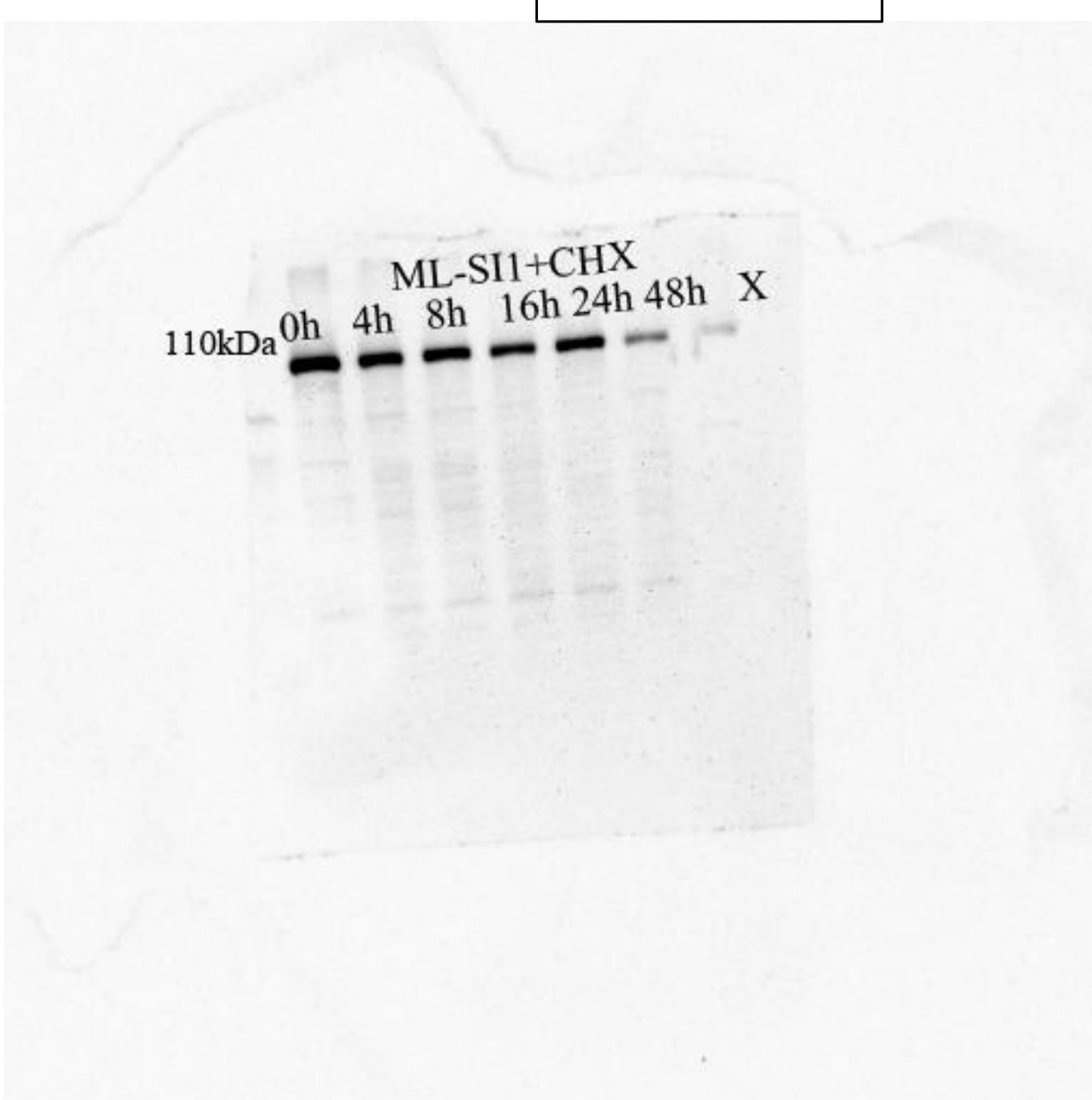

GAPDH

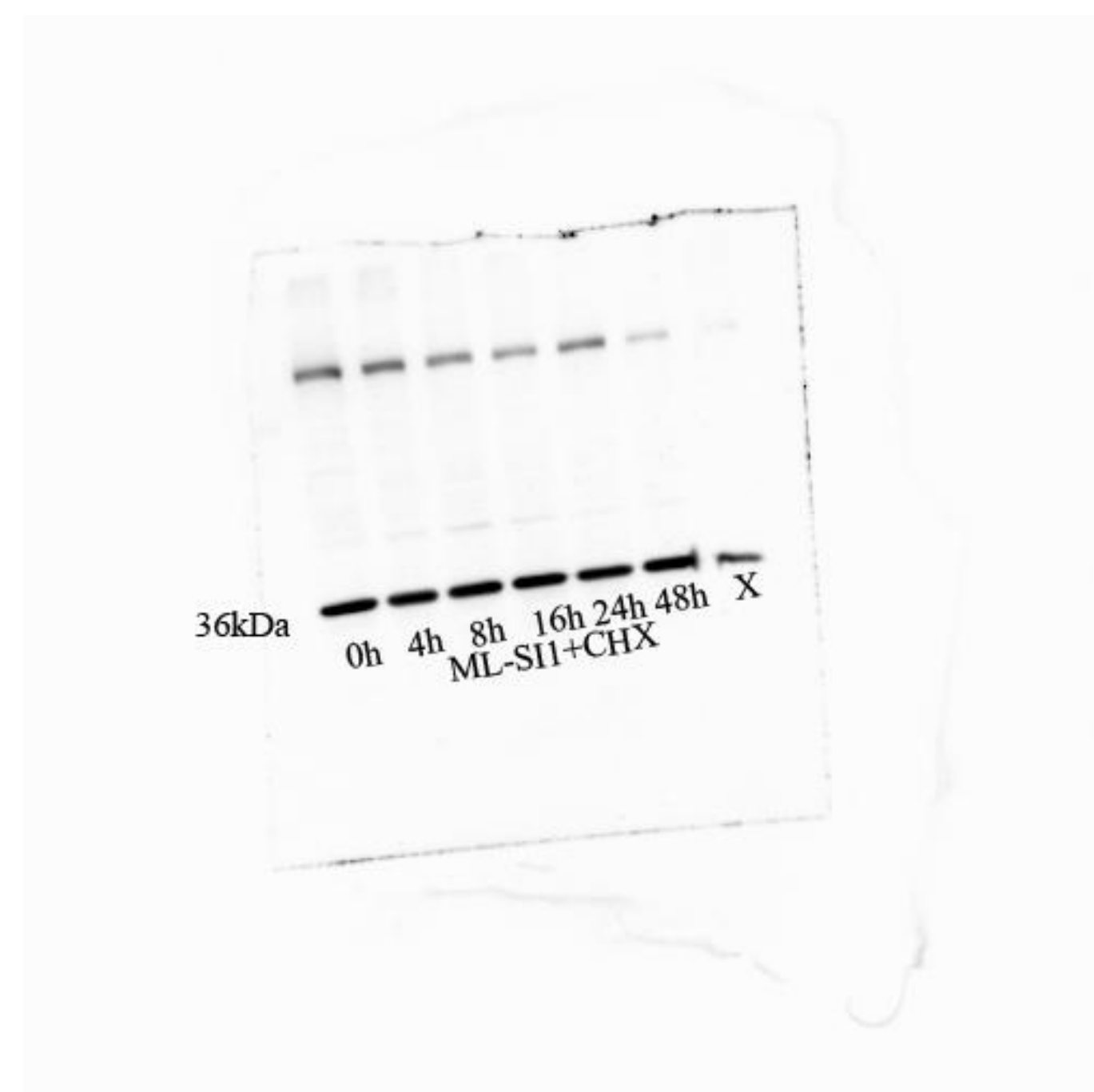

Figure 6D Group 3

AR

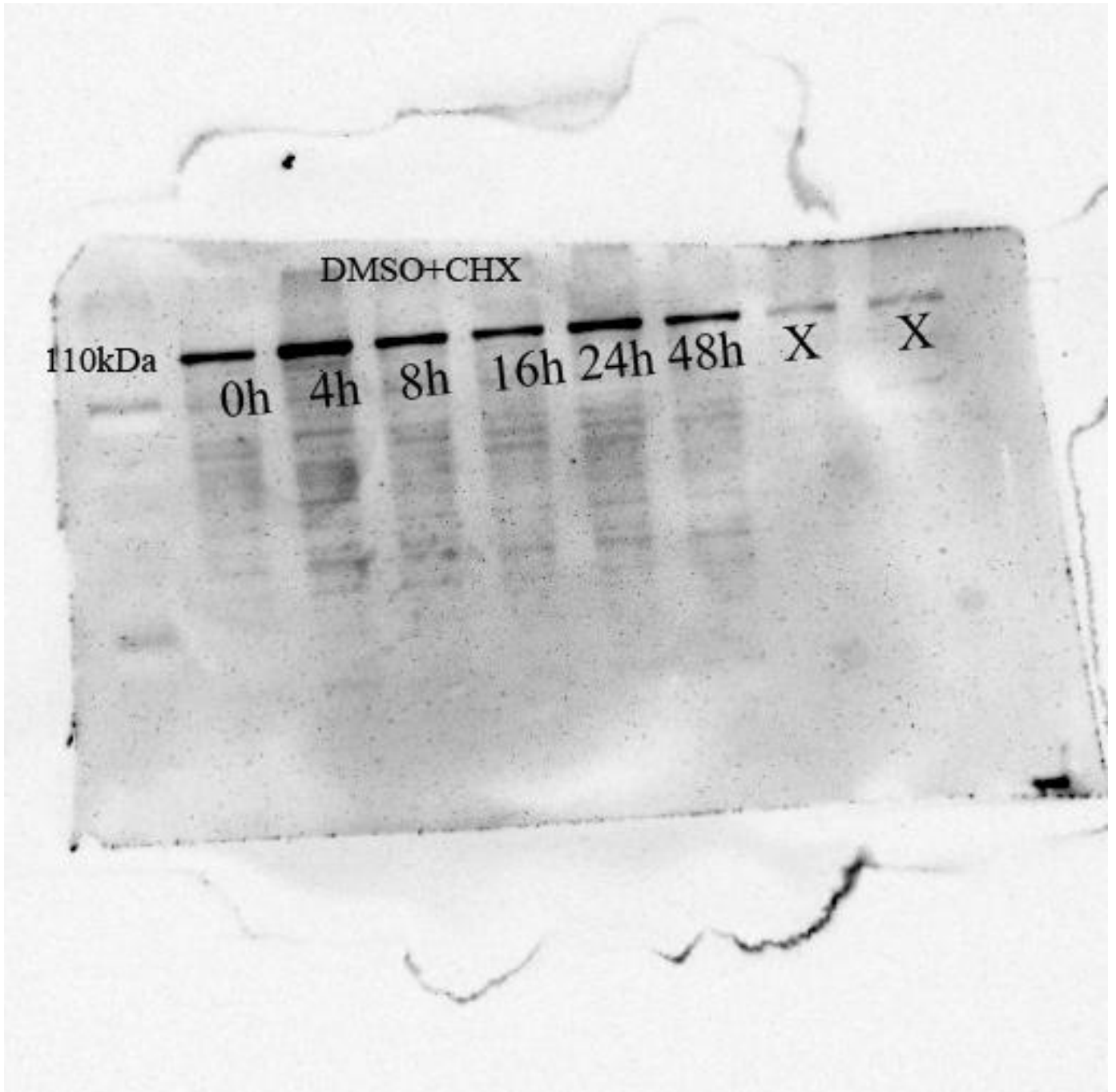

GAPDH

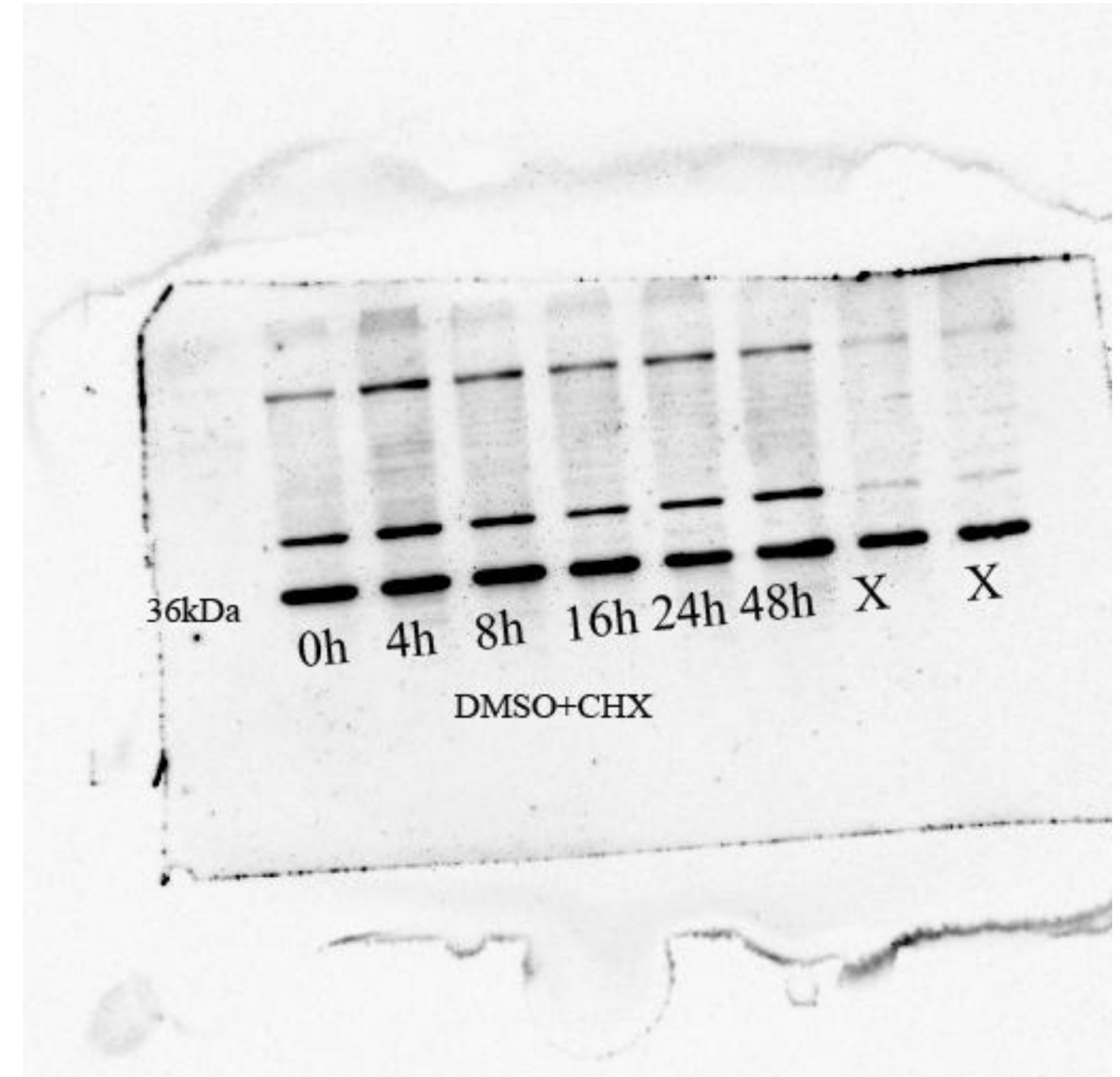

AR

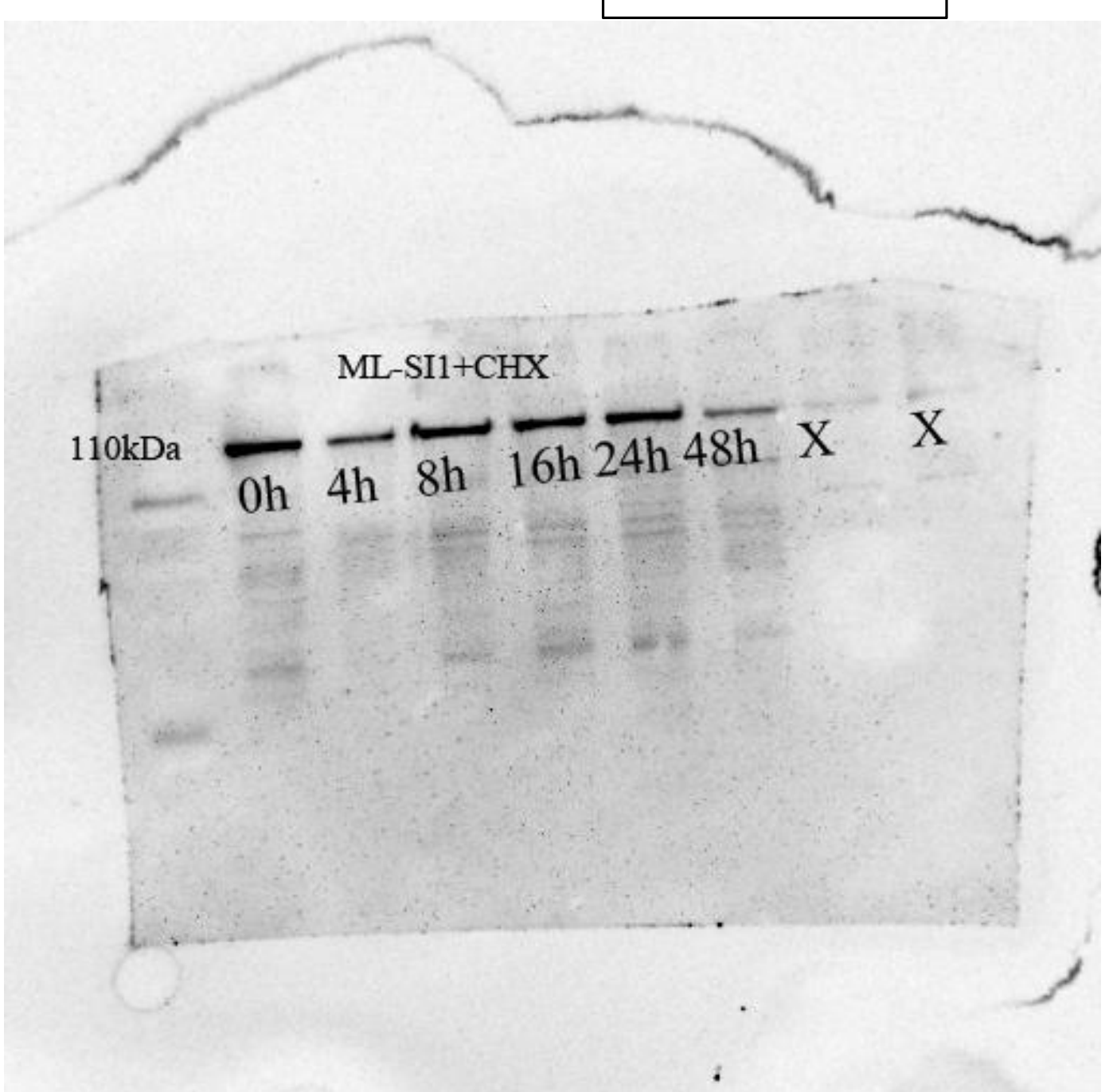

GAPDH

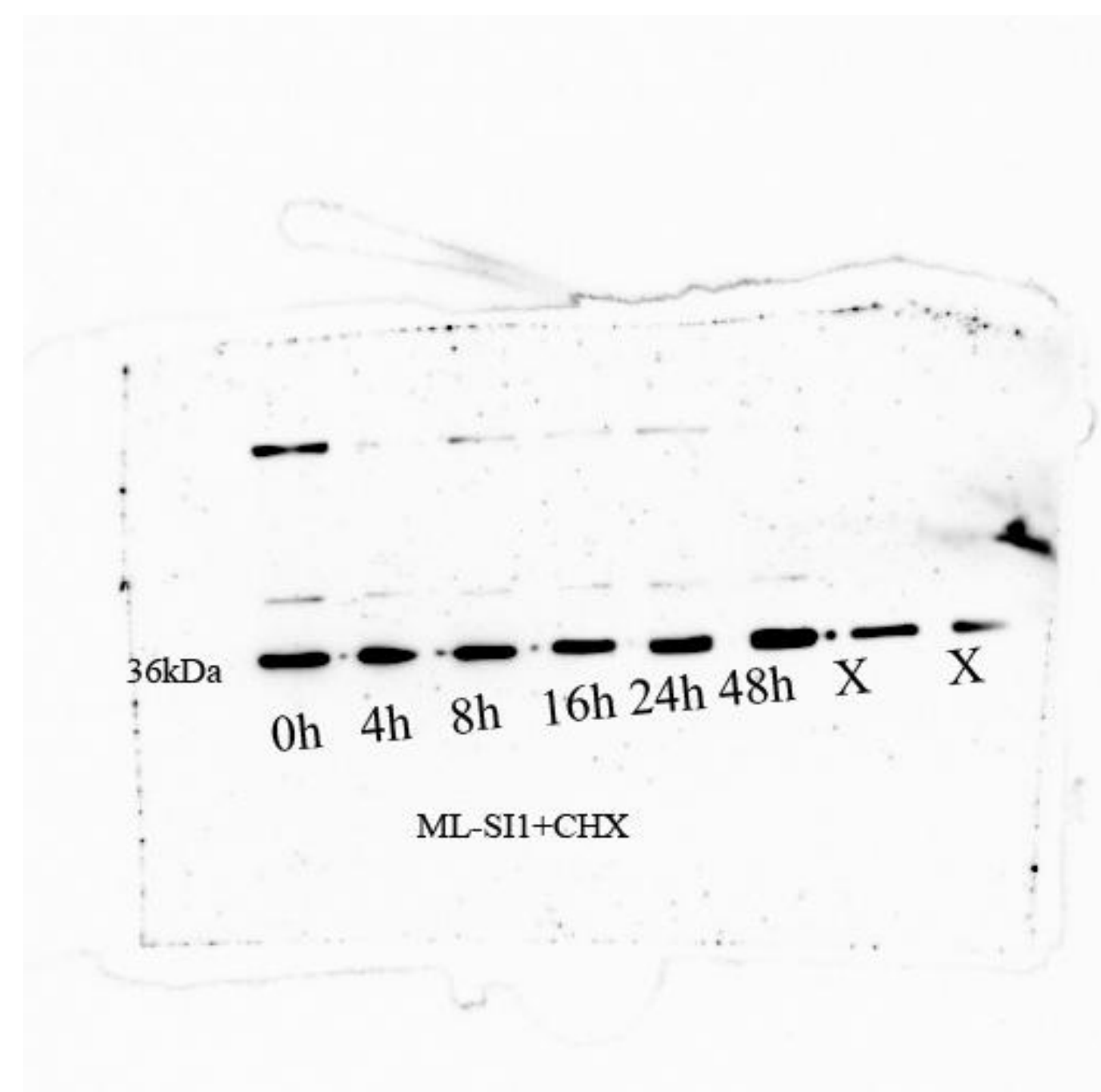

Figure 6F Group 1

AR

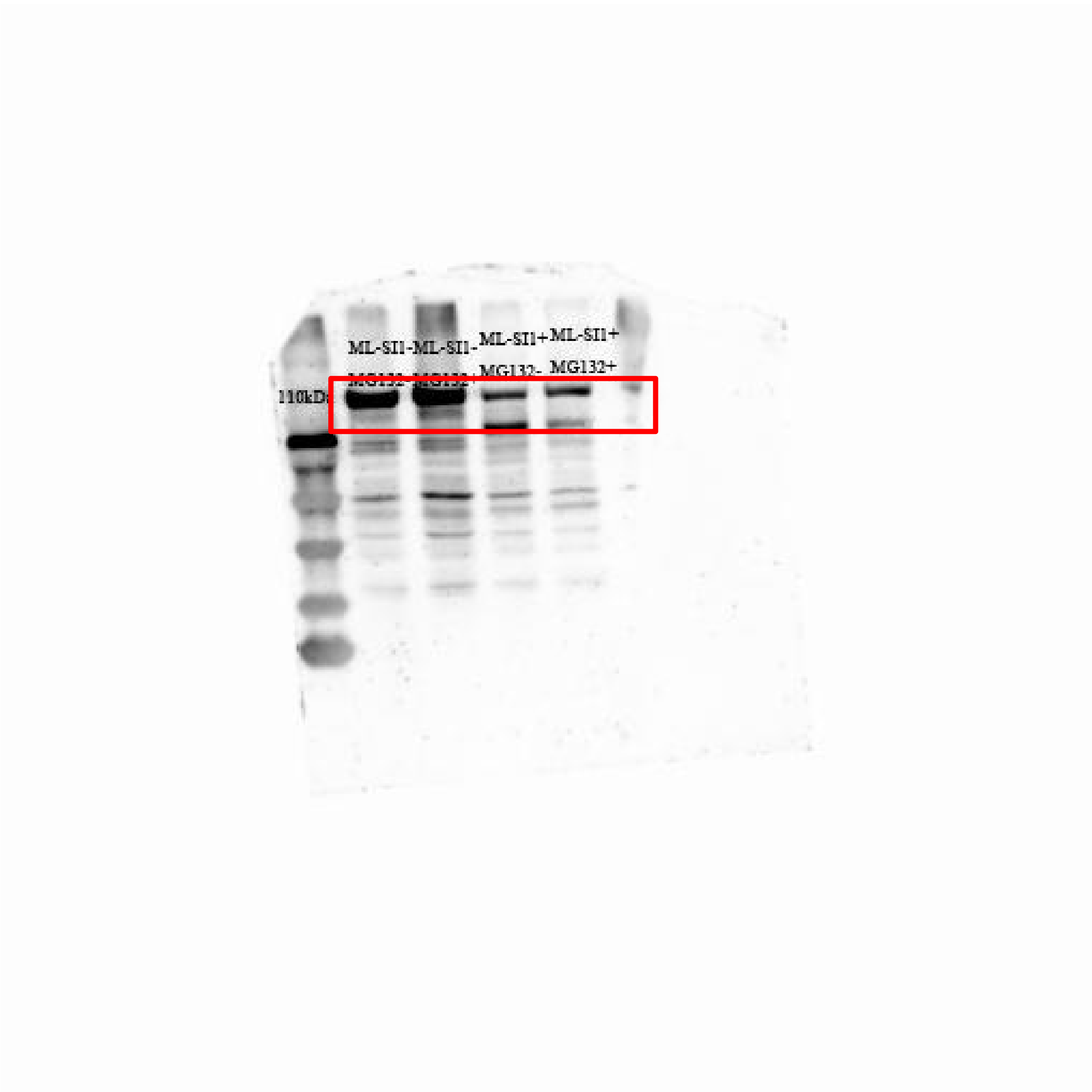

GAPDH

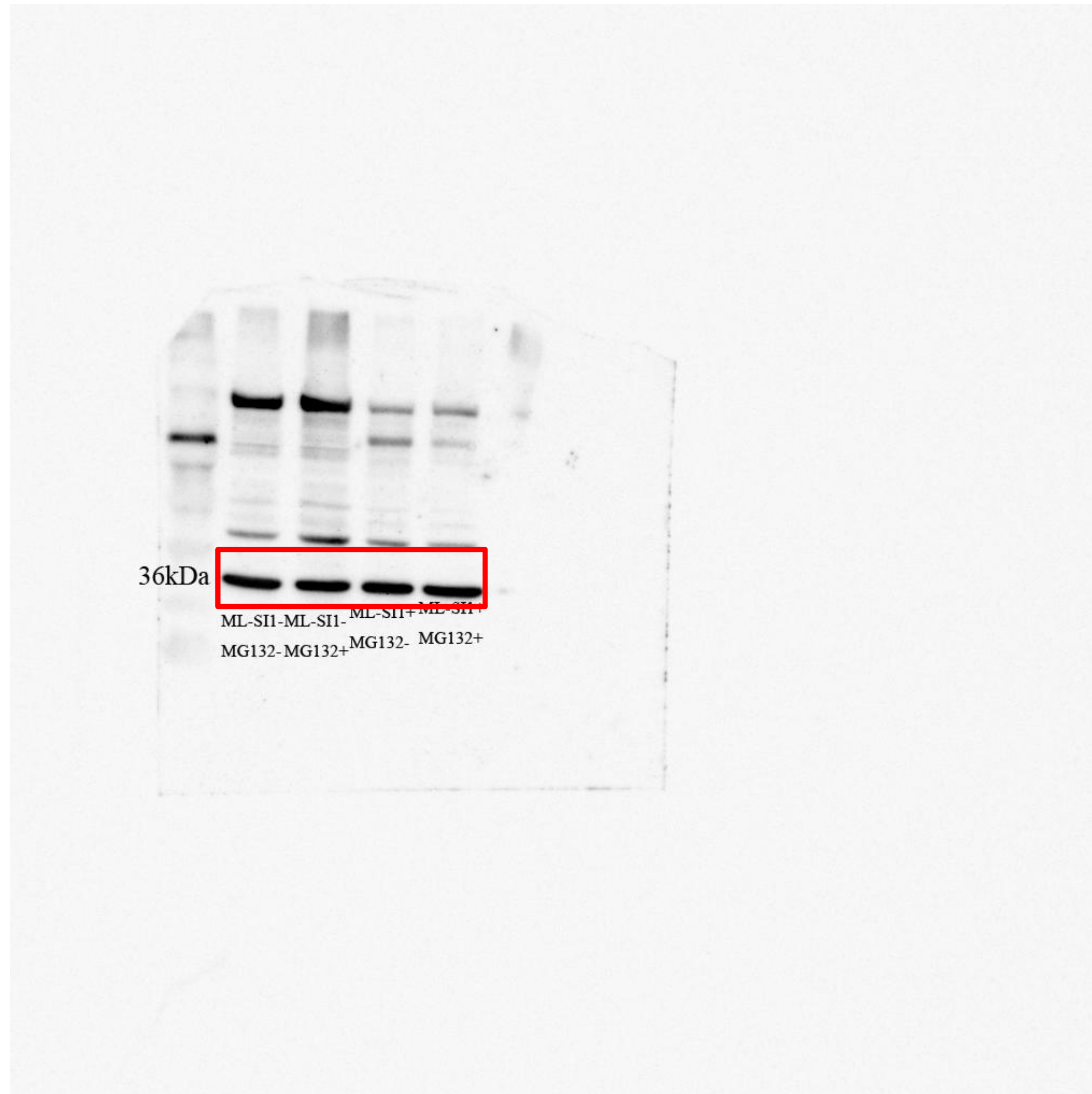

Figure 6F   Group 2

AR

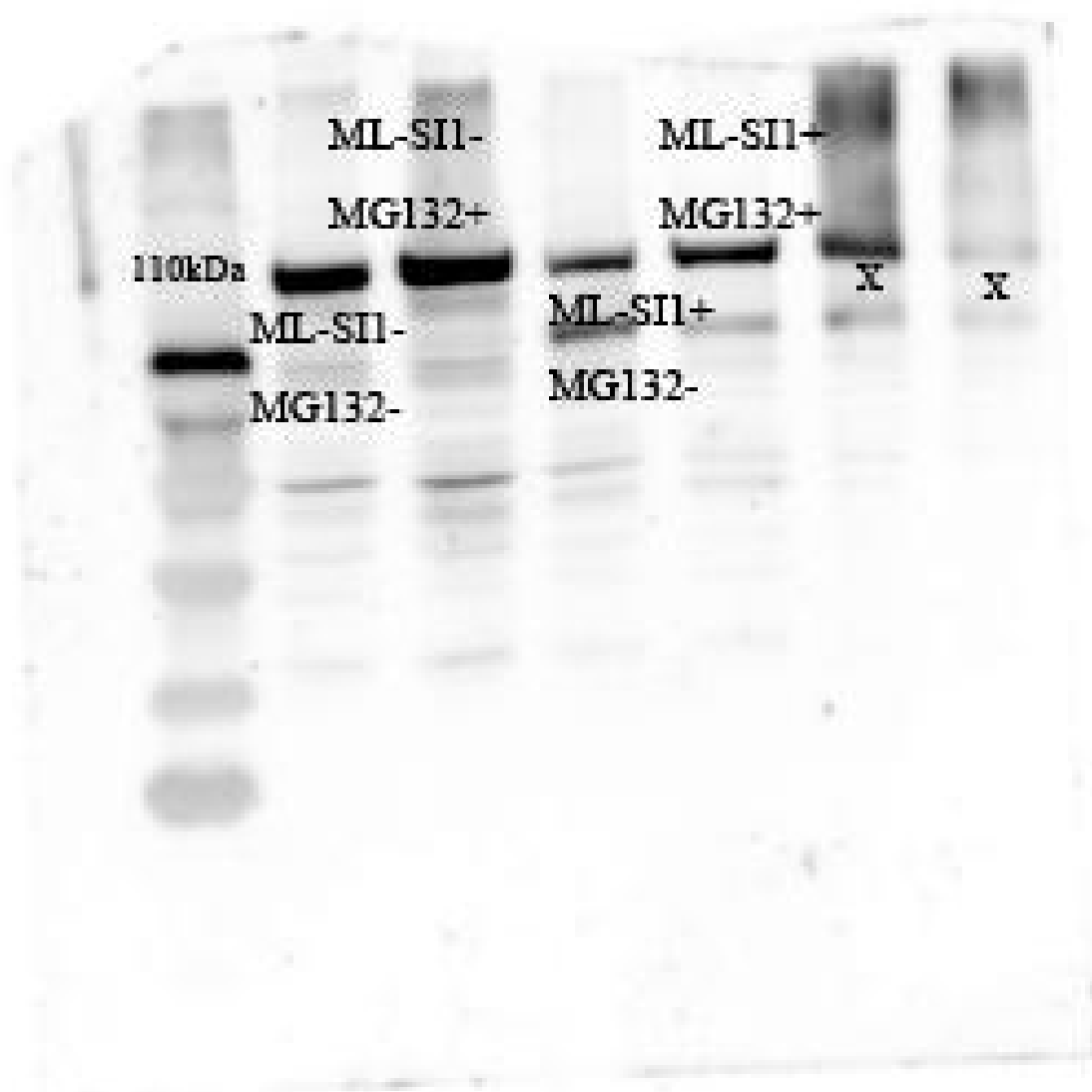

GAPDH

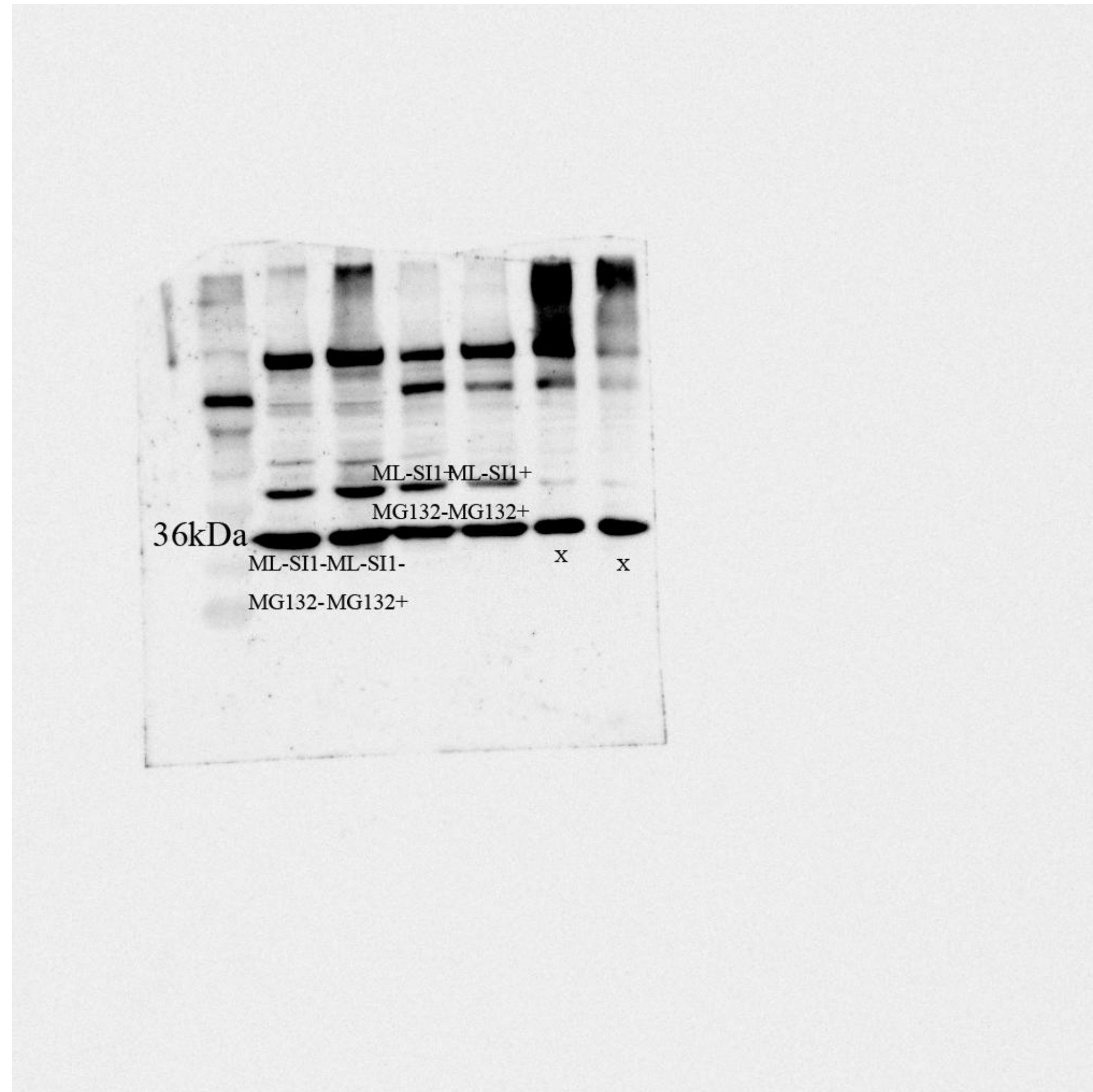

Figure 6F   Group 3

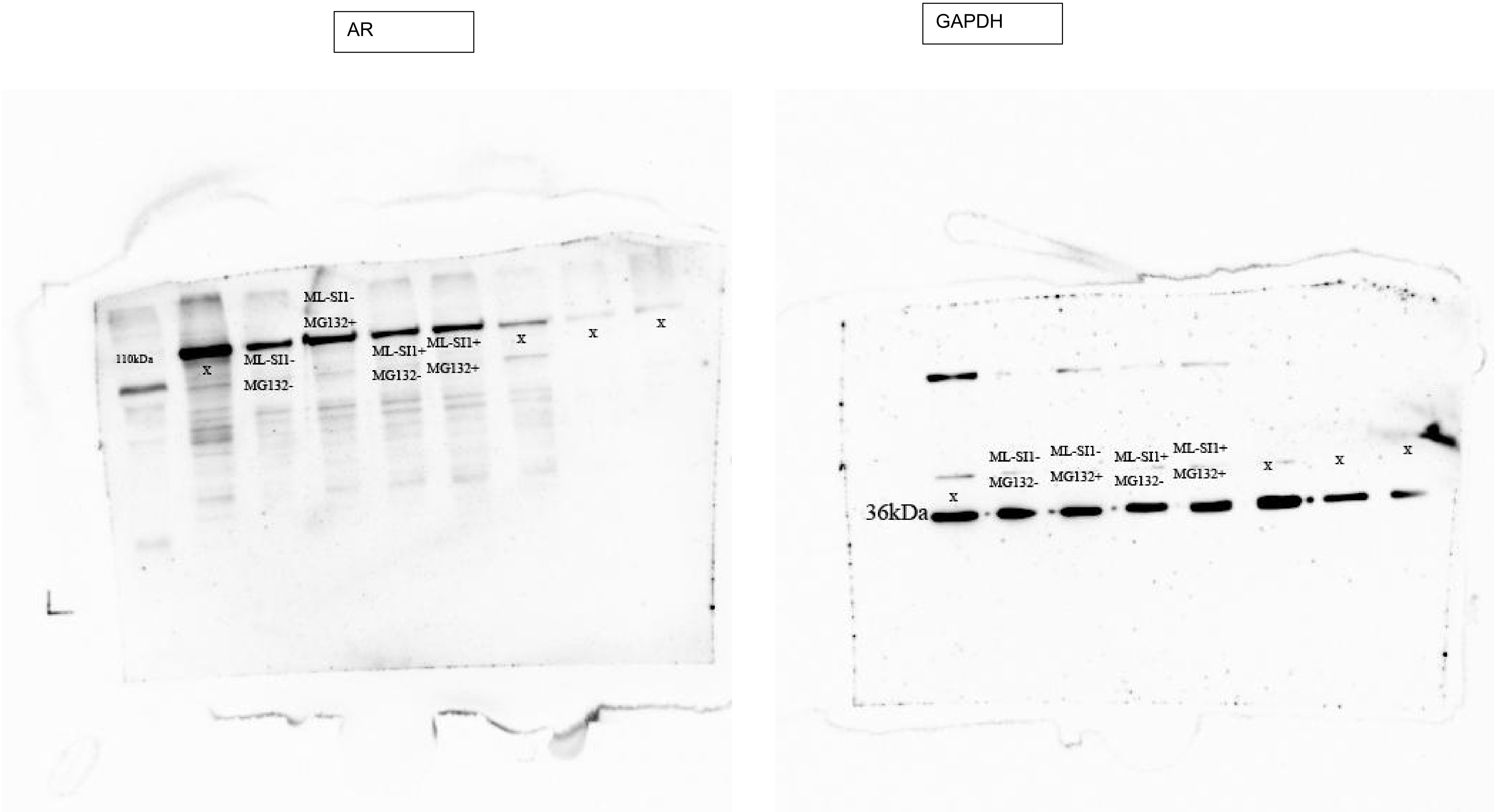

Figure 7E Group 1

ODC1

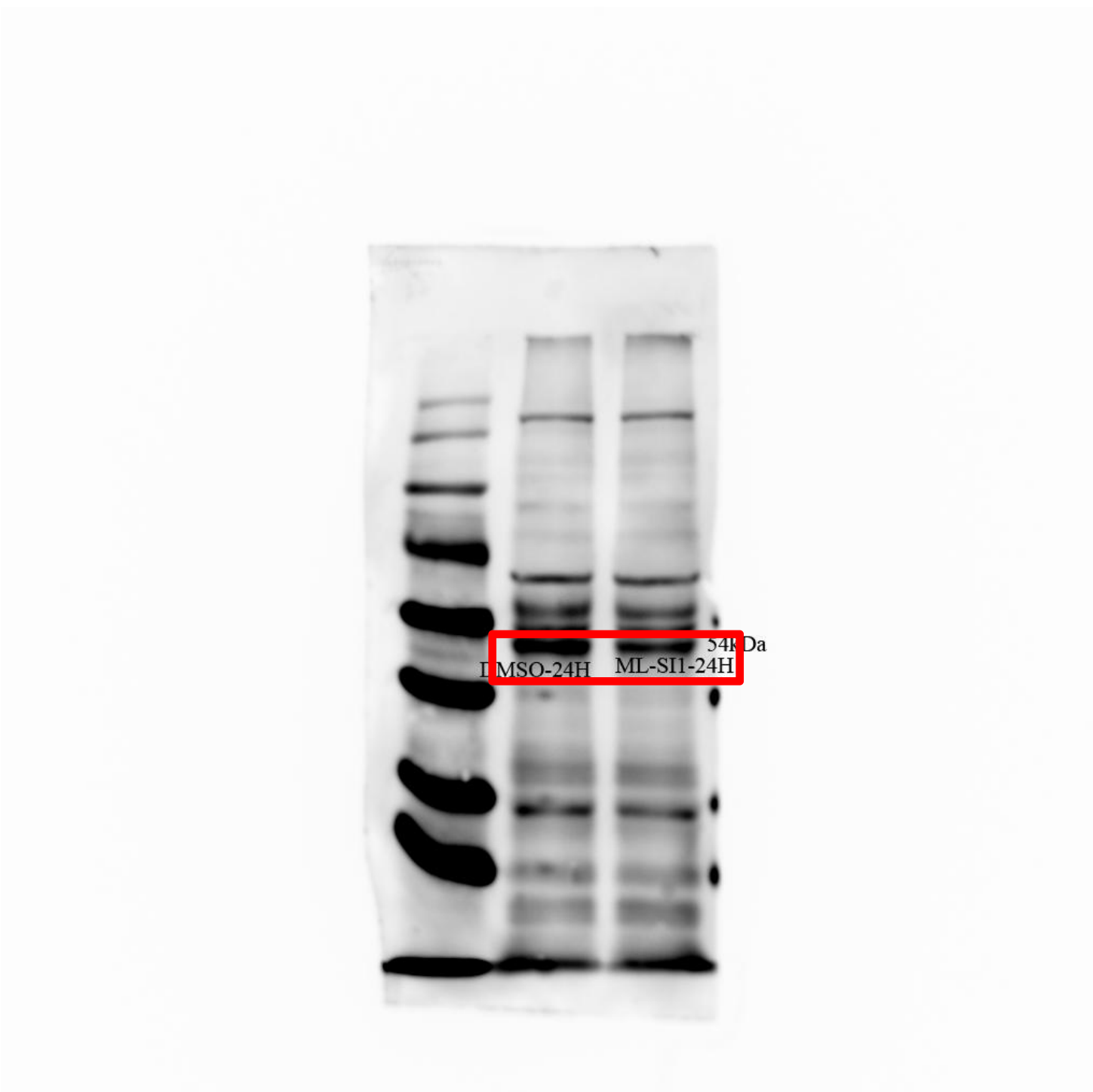

SMS

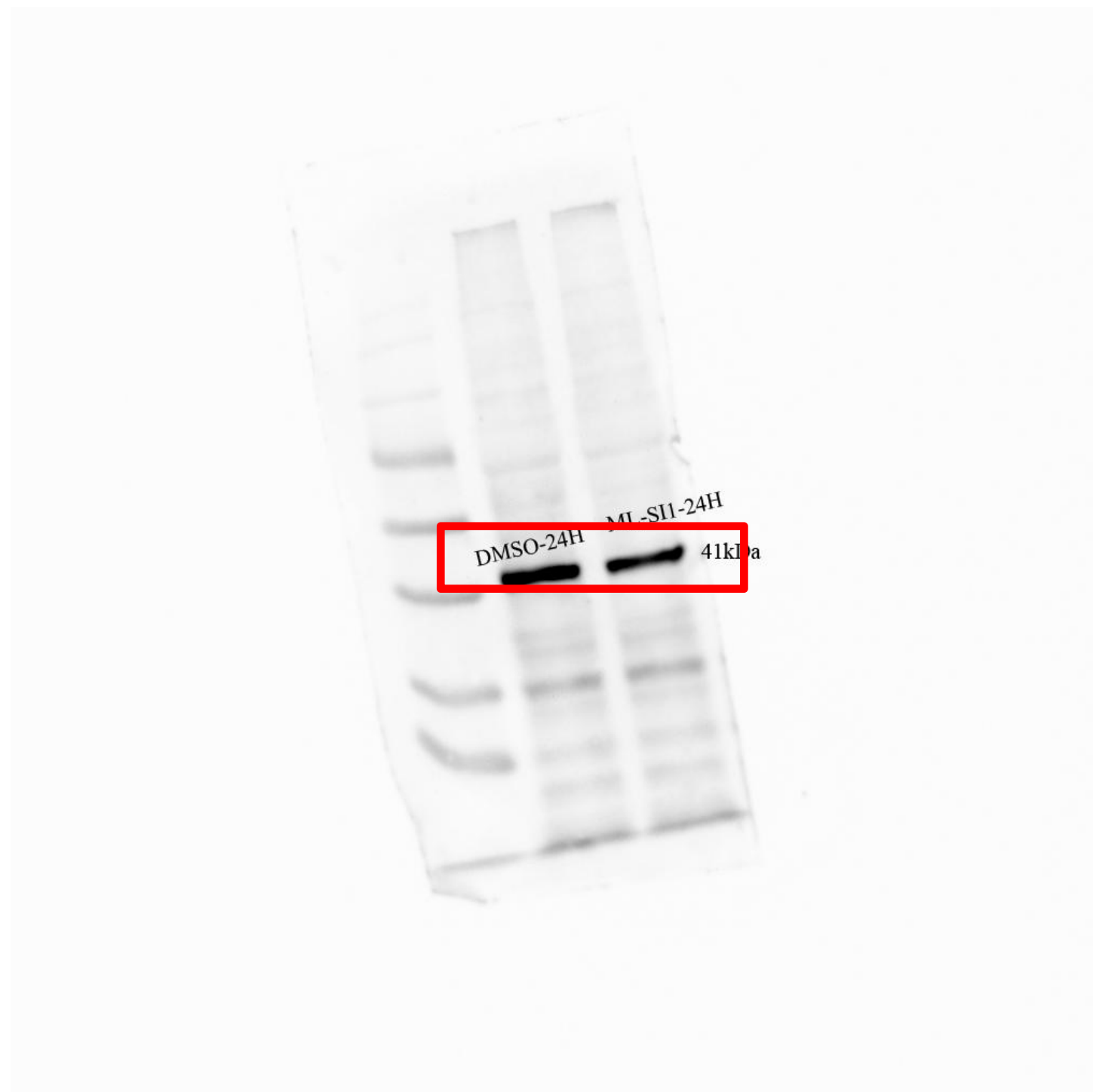

GAPDH

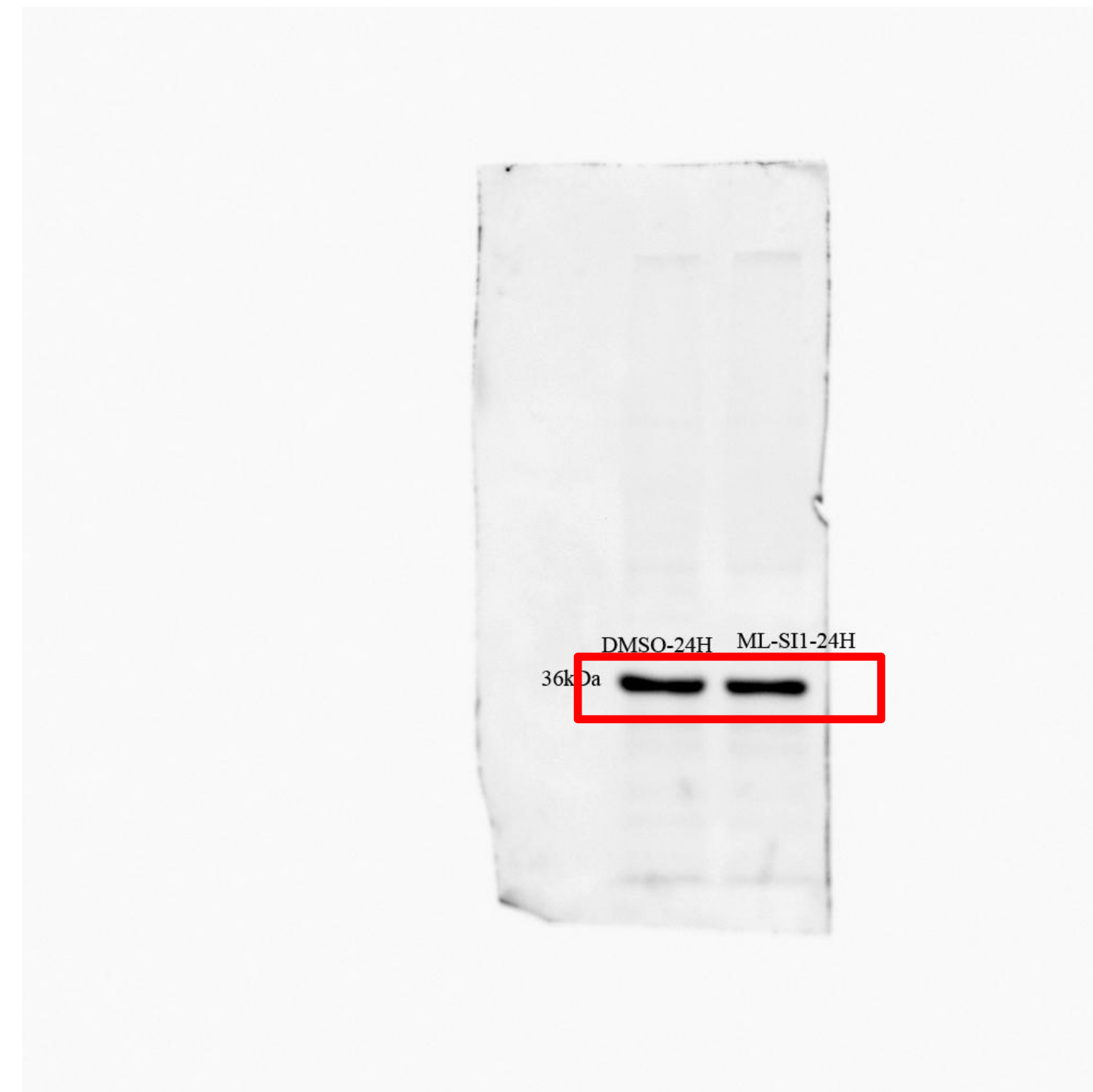

Figure 7E Group 2

ODC1

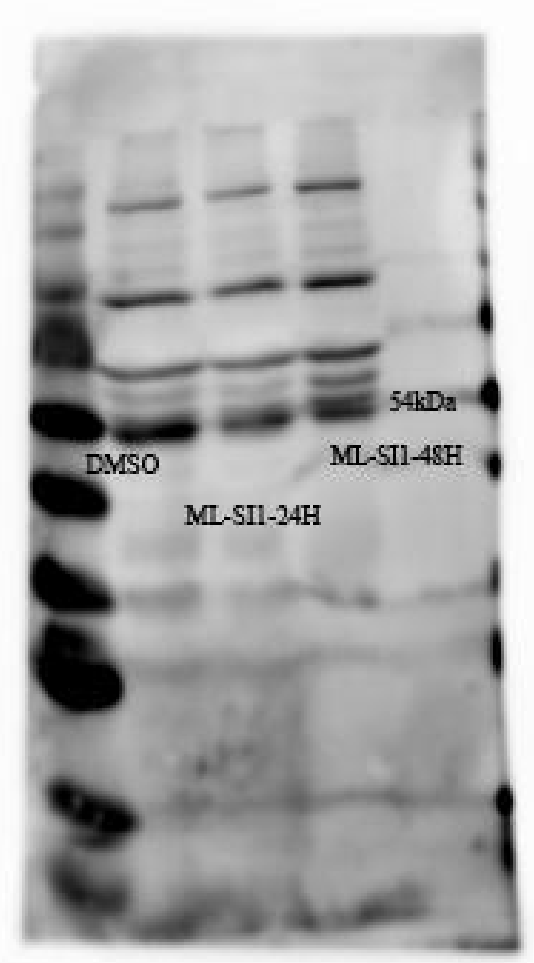

SMS

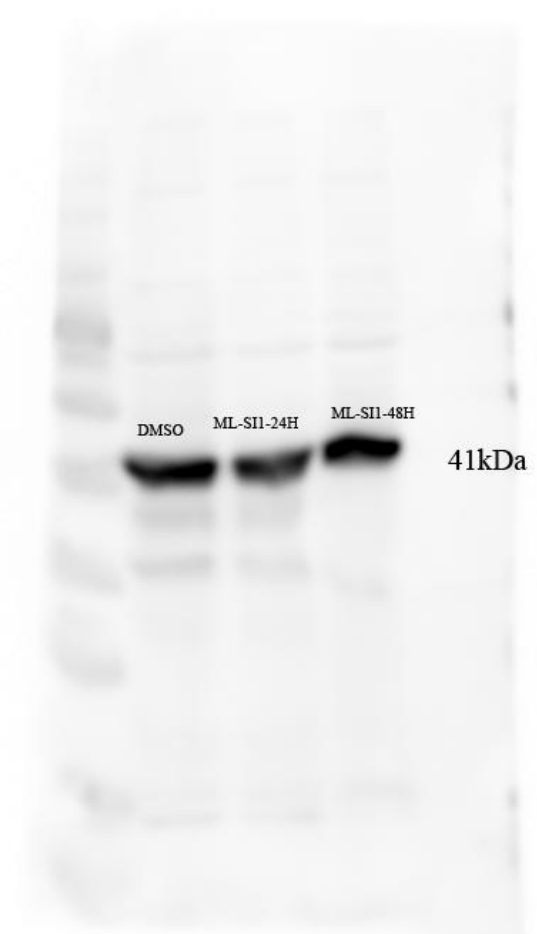

GAPDH

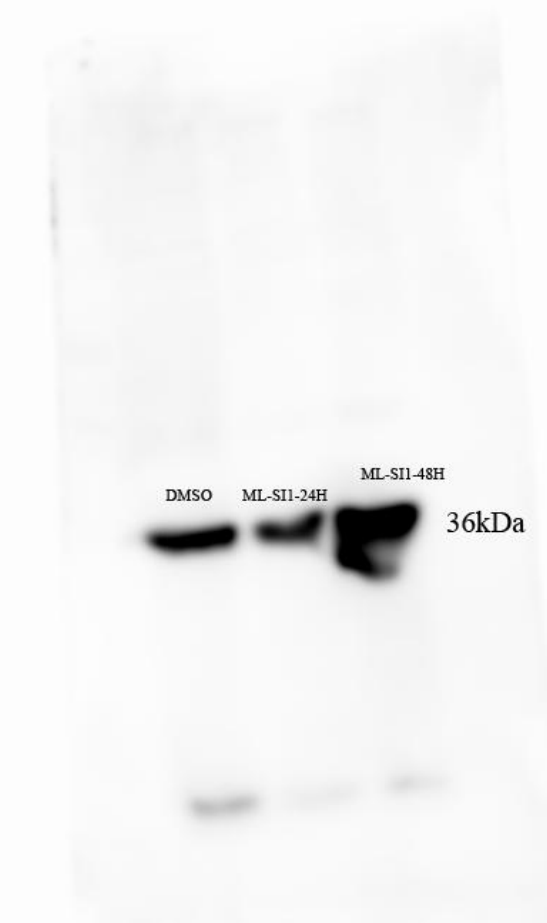

Figure 7E   Group 3

ODC1

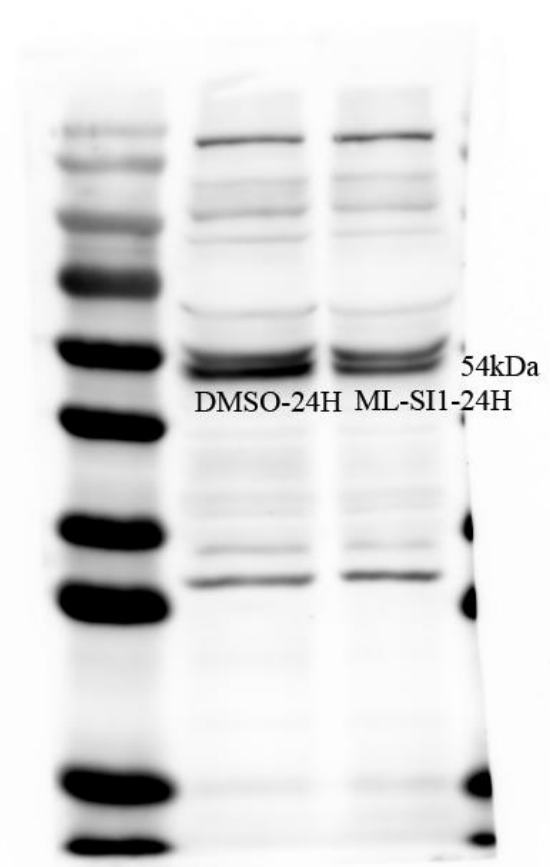

SMS

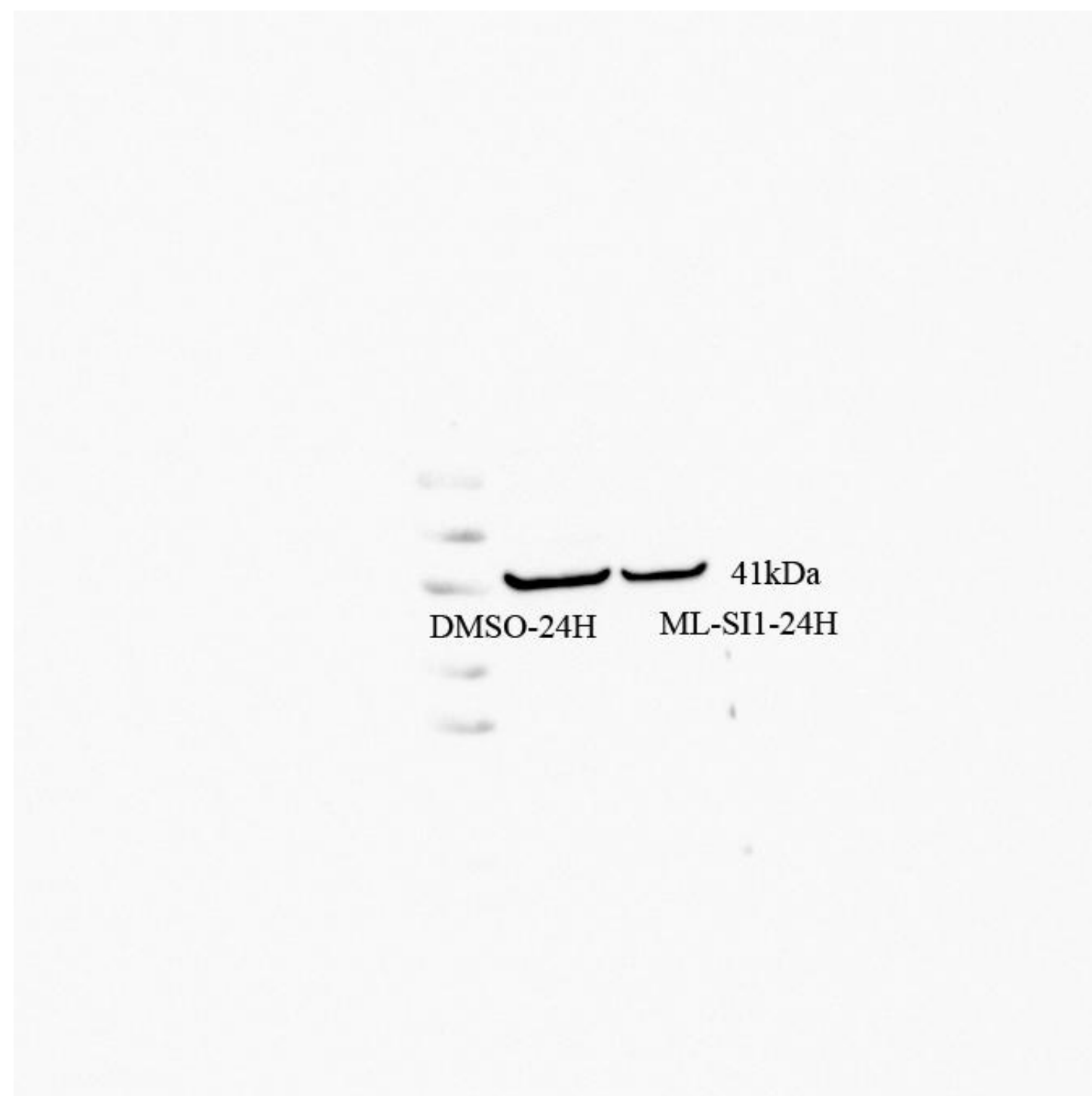

GAPDH

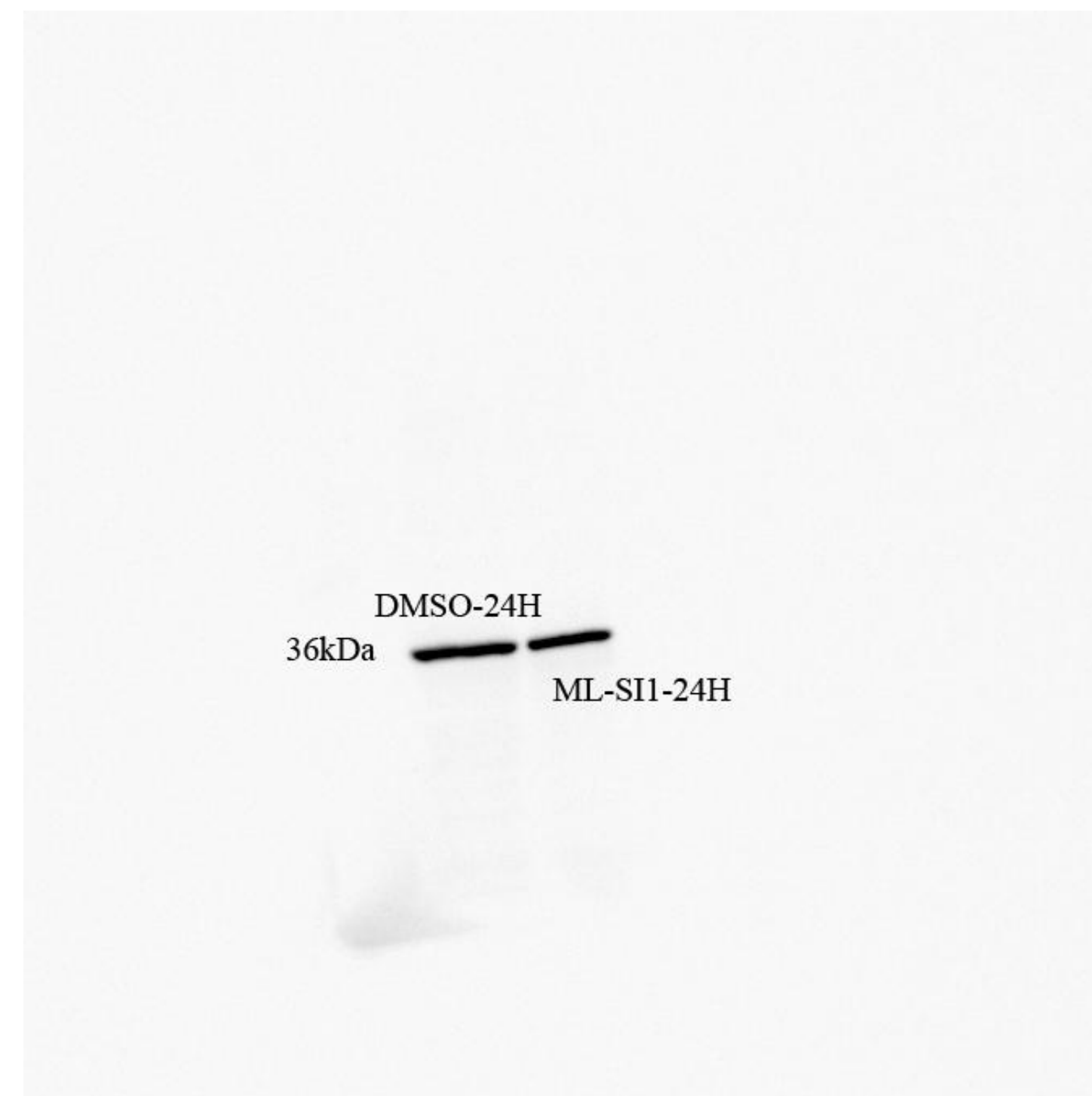

Figure 7F Group 1

ODC1

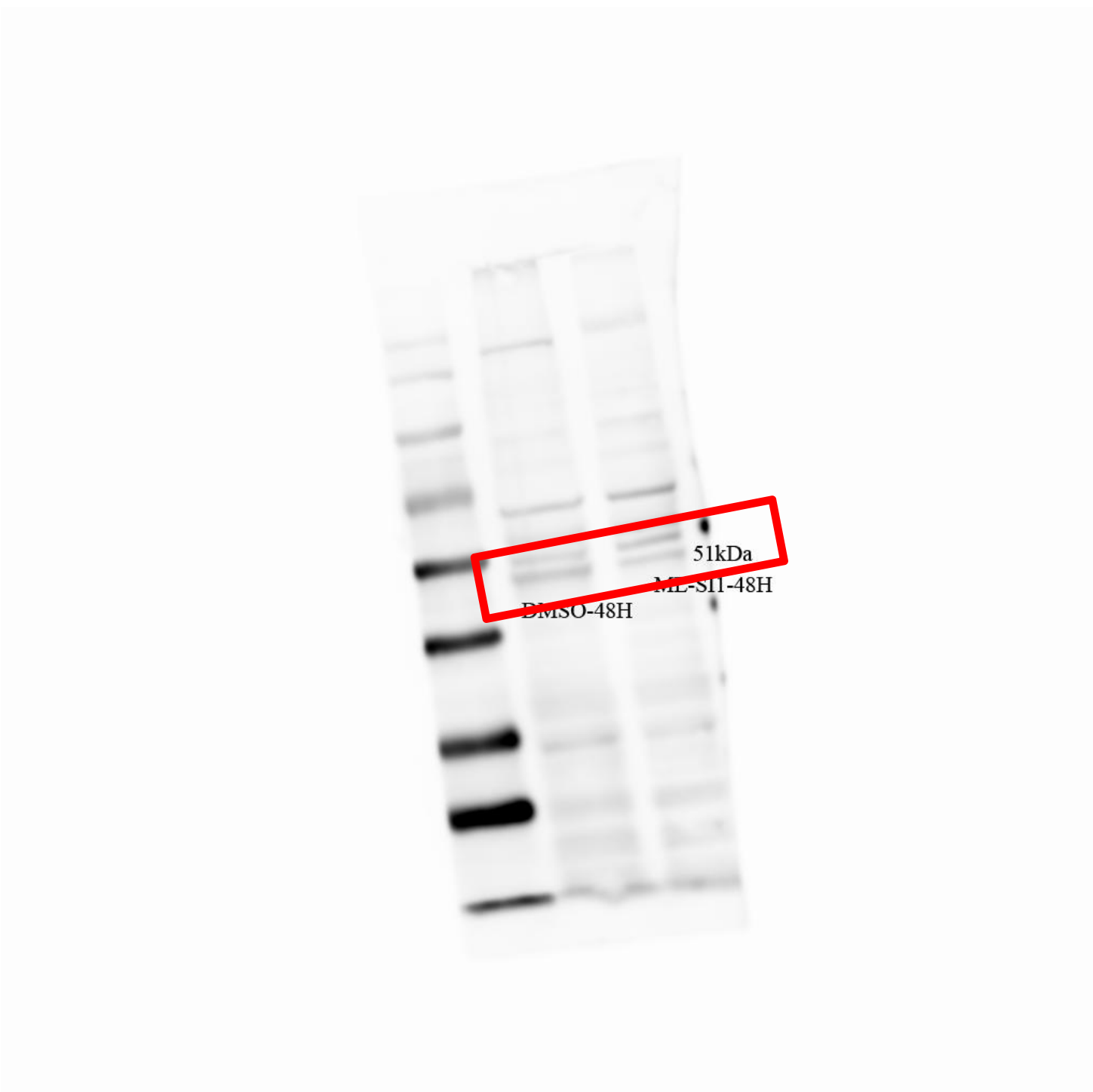

SMS

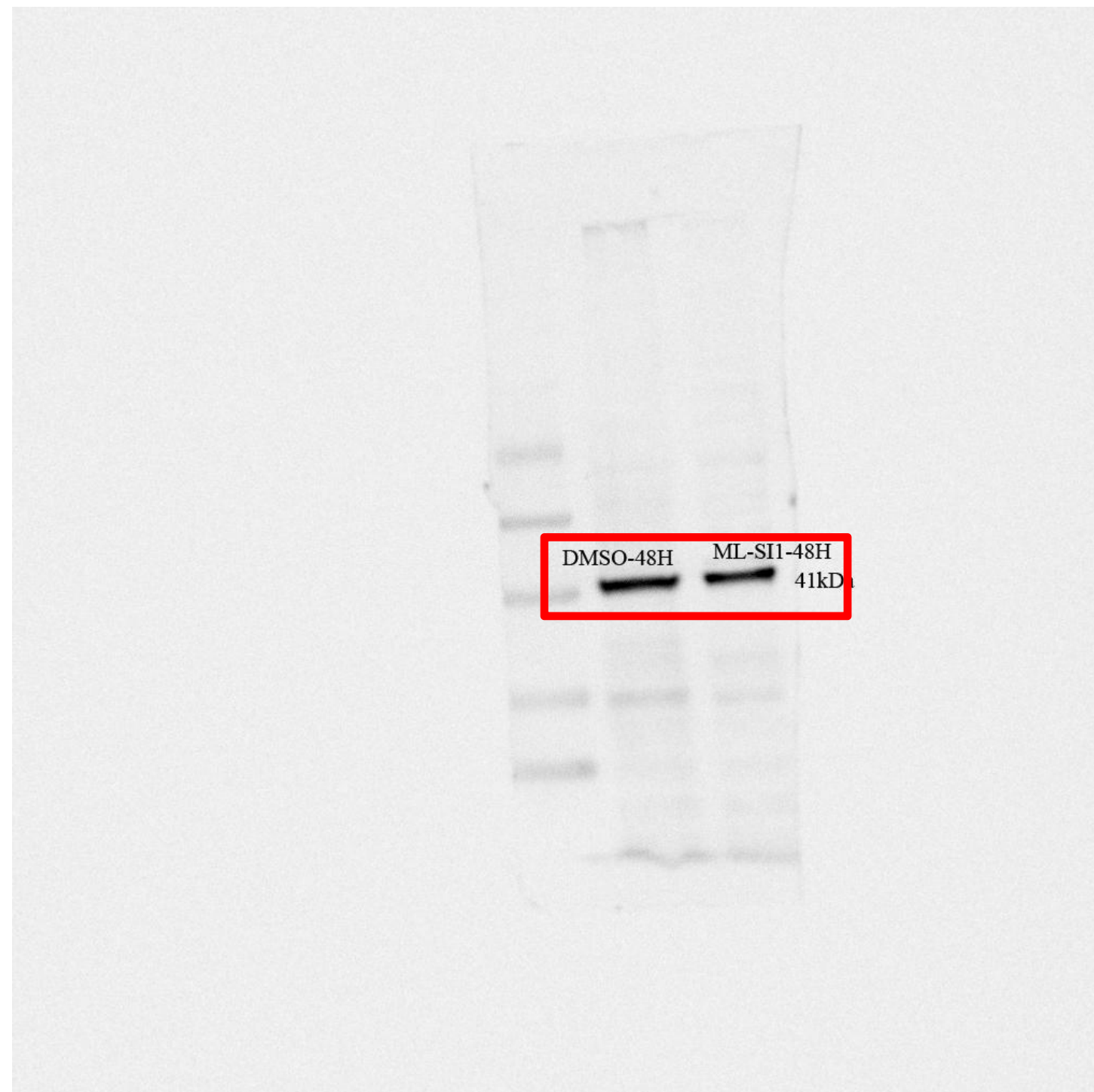

GAPDH

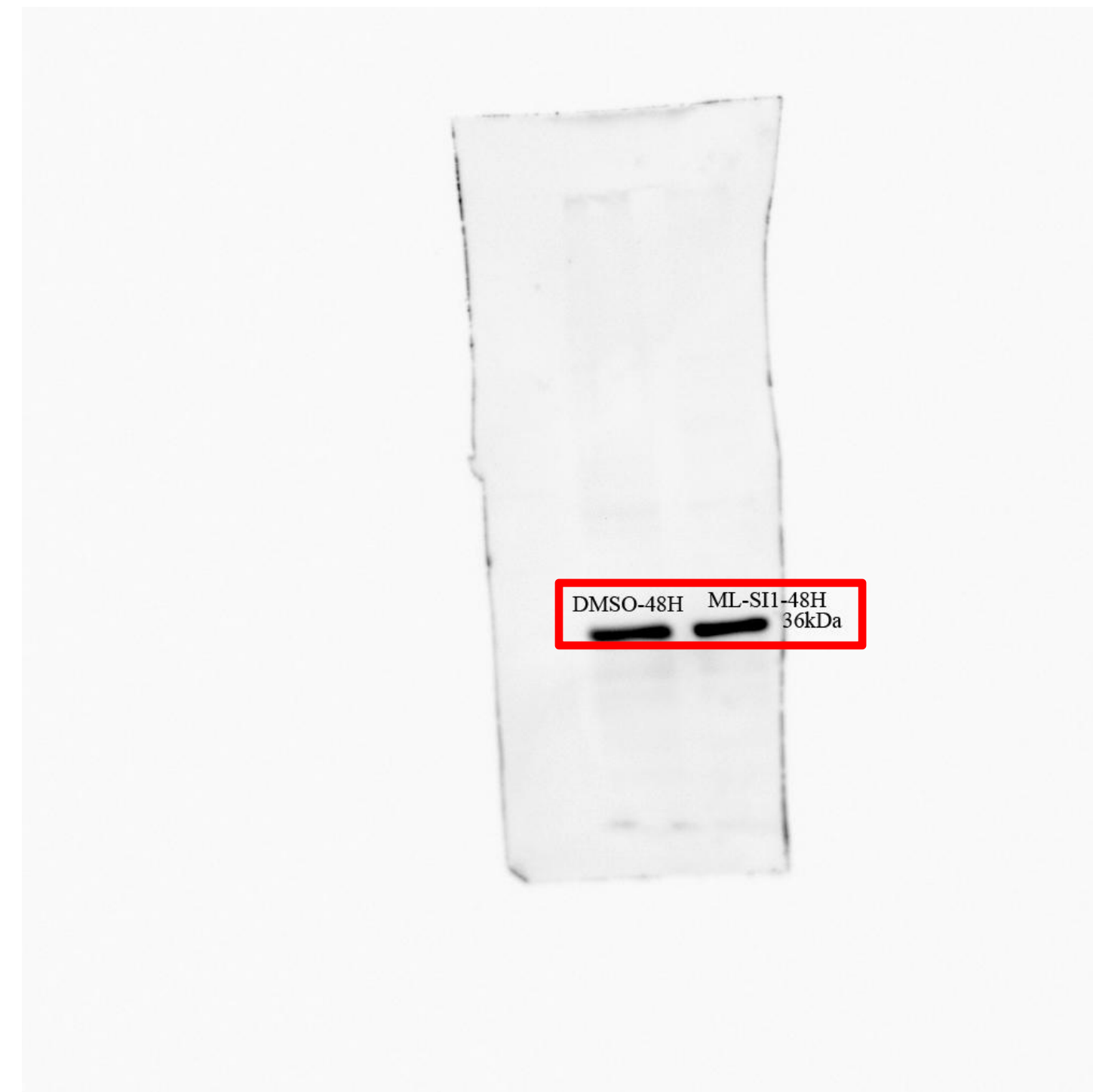

Figure 7F Group 2

ODC1

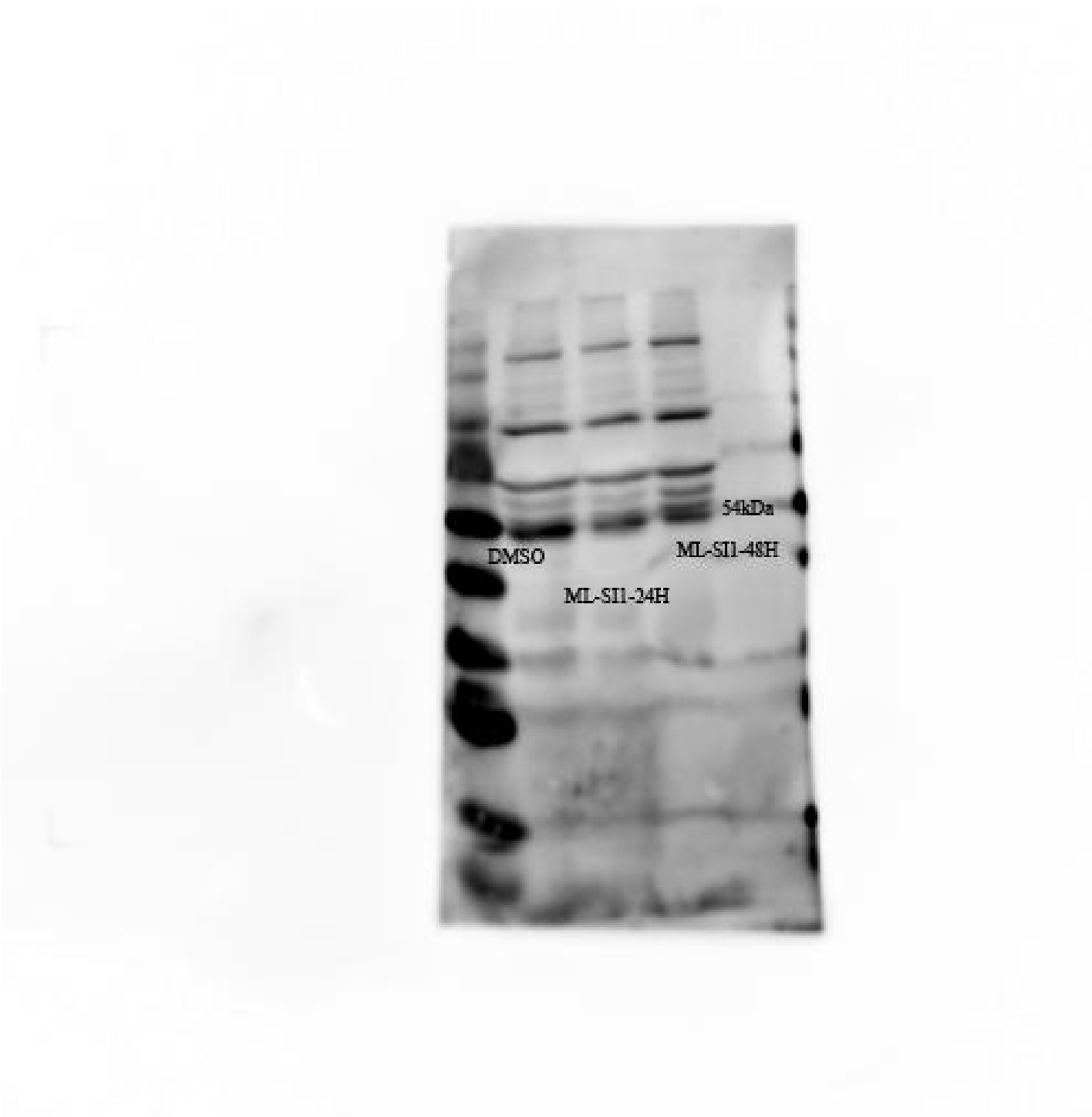

SMS

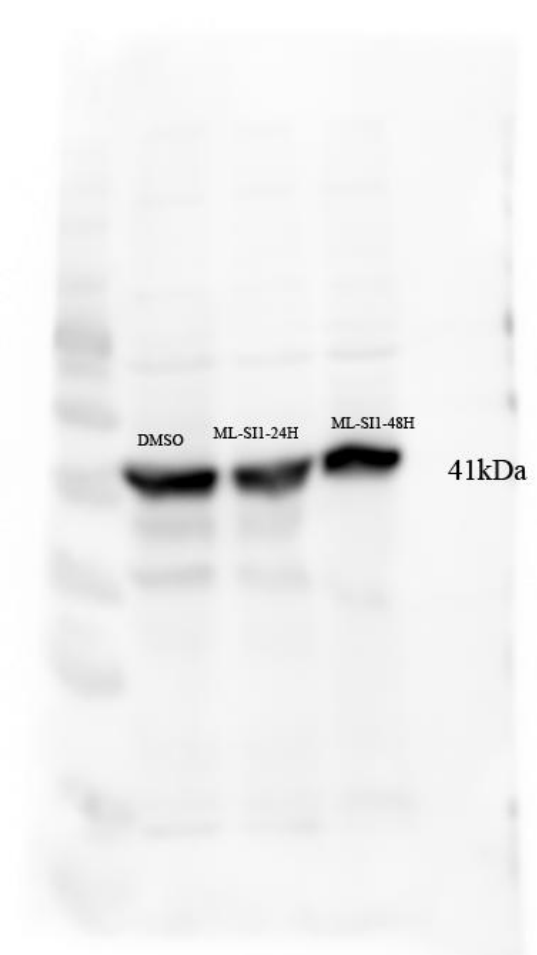

GAPDH

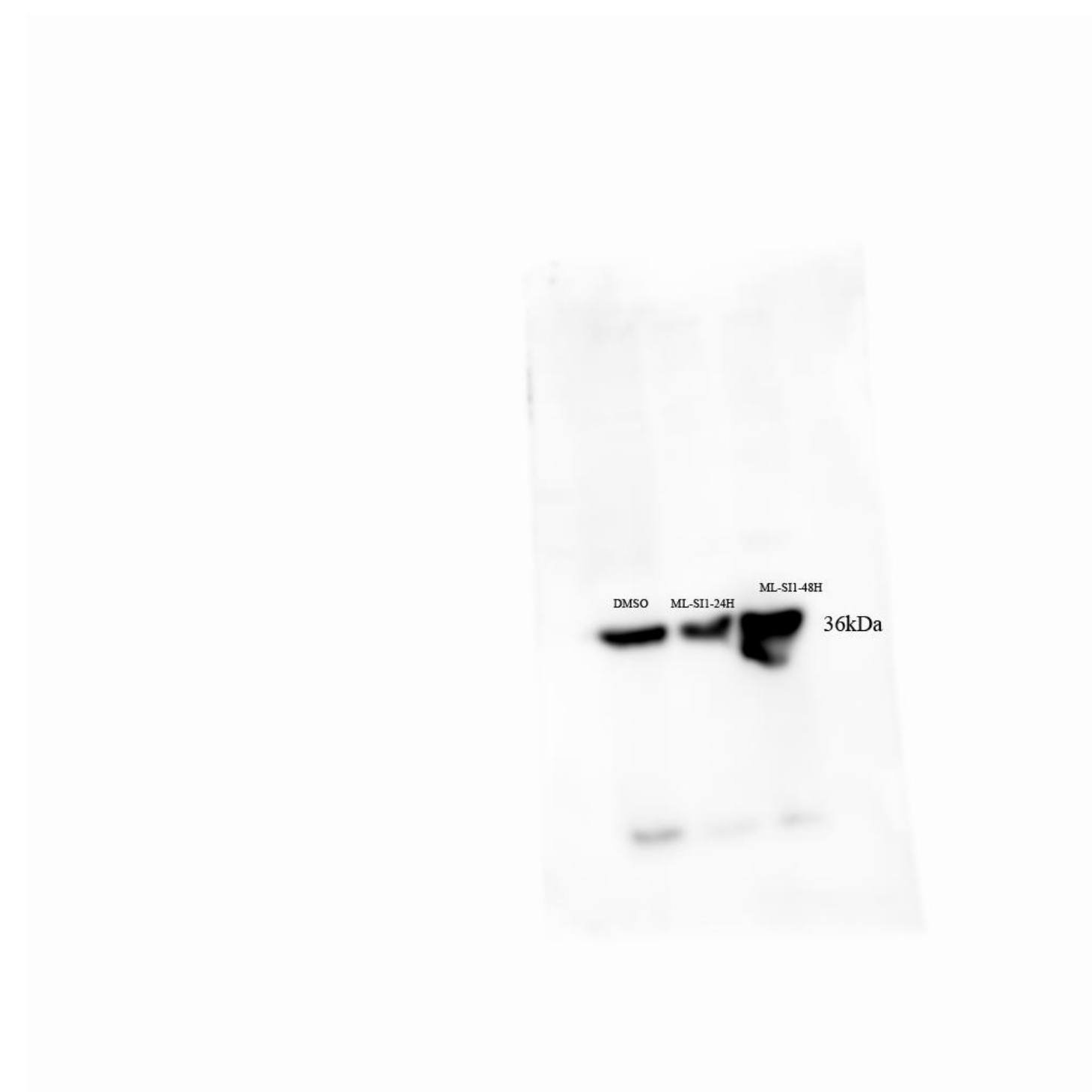

Figure 7F Group 3

ODC1

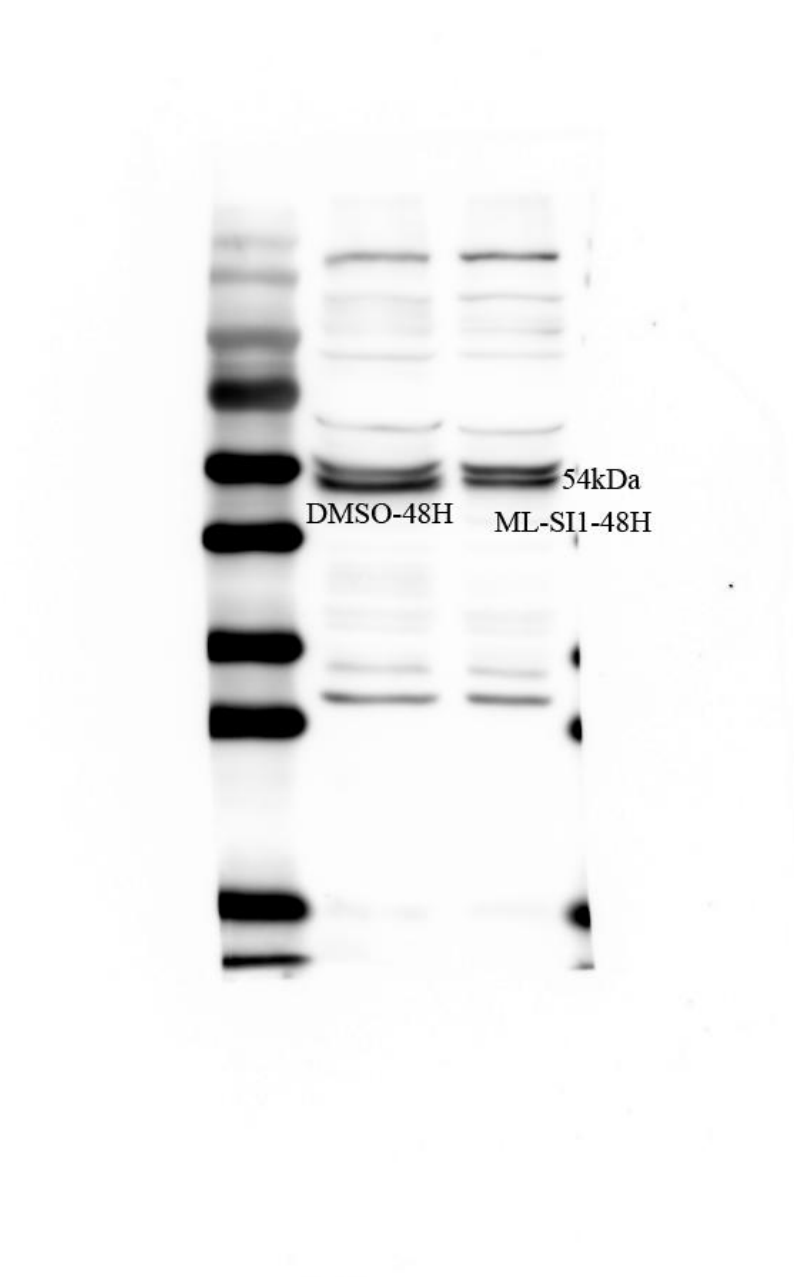

SMS

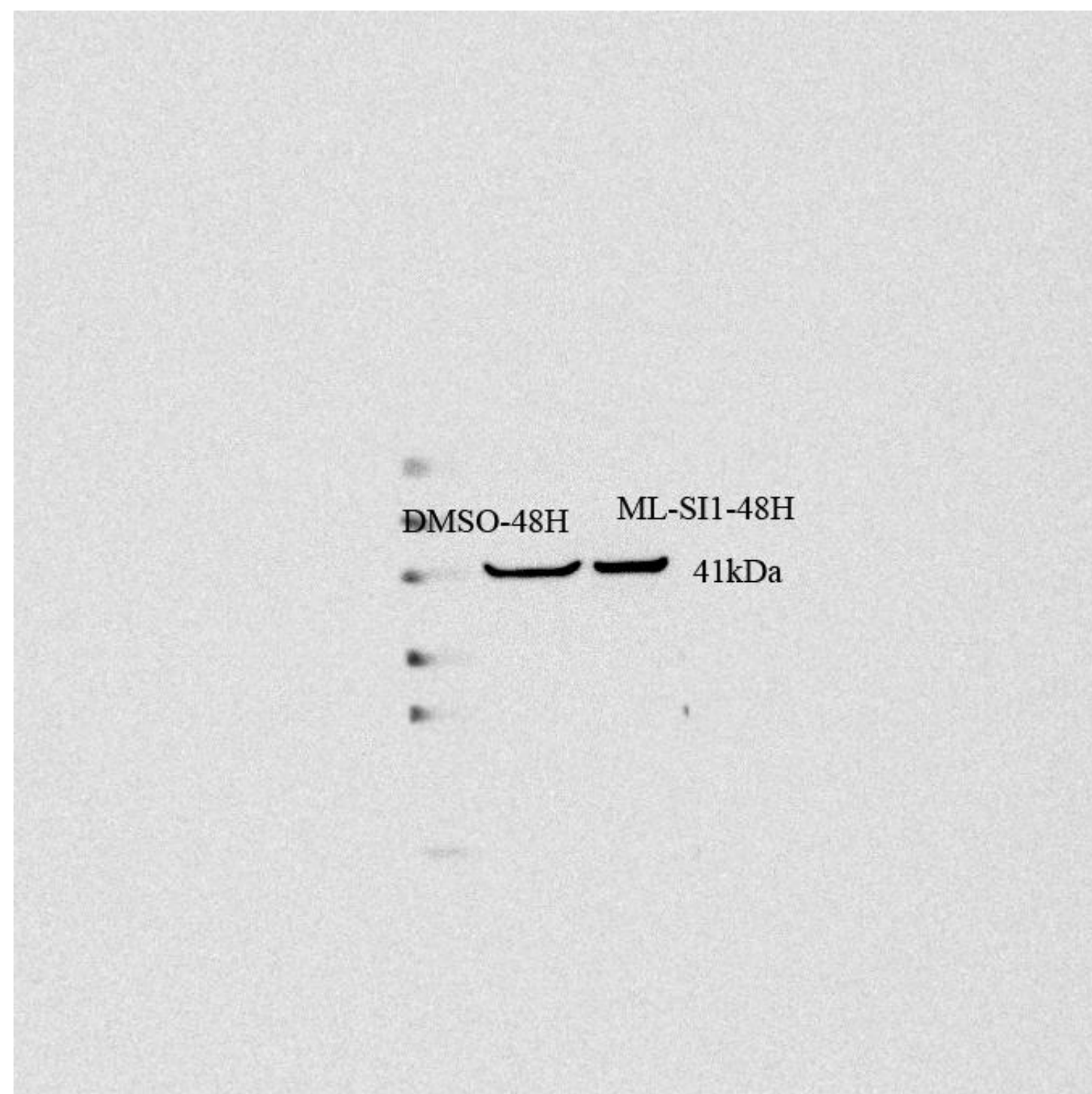

GAPDH

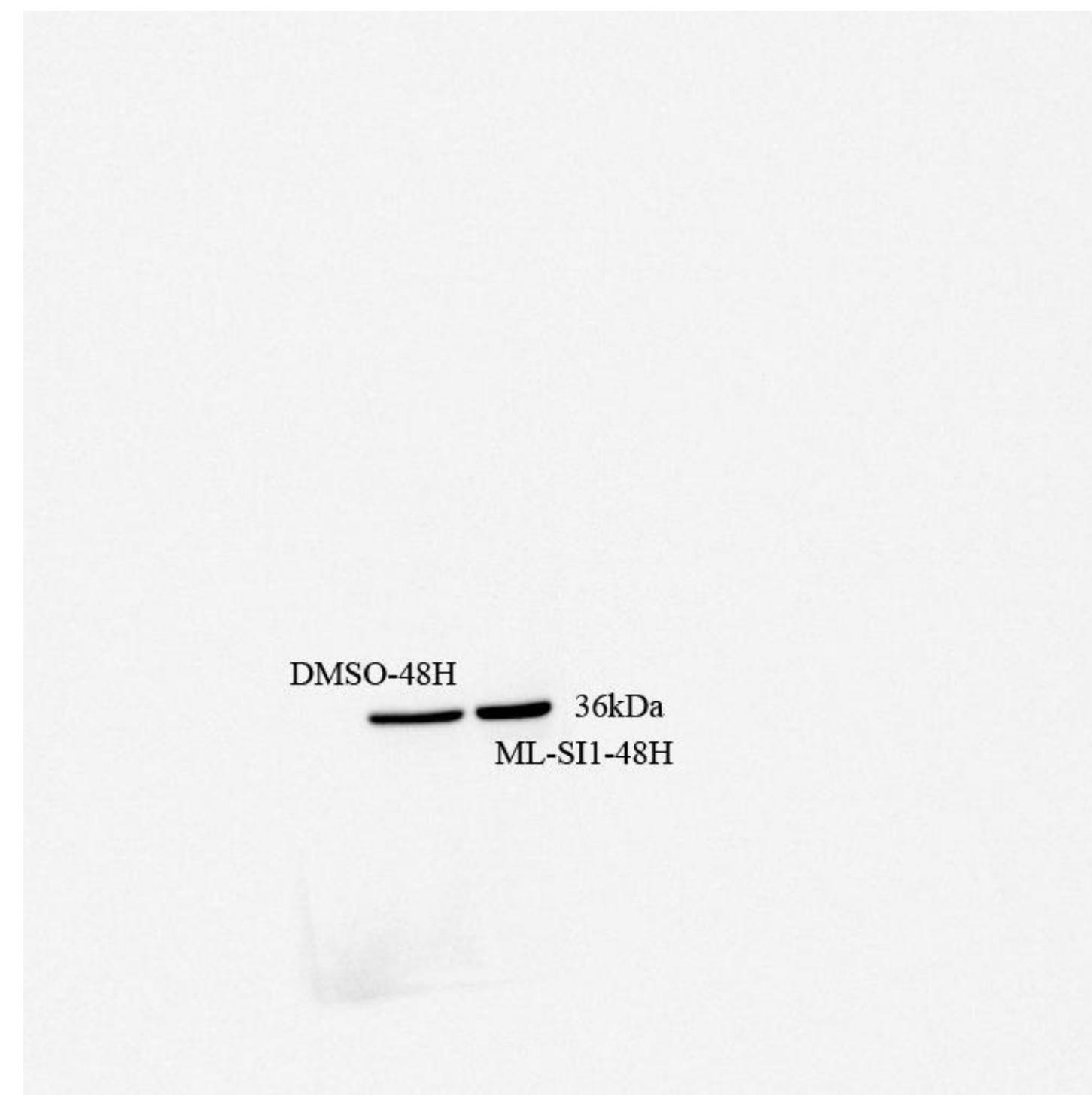

Figure 8E Group 1

ODC1

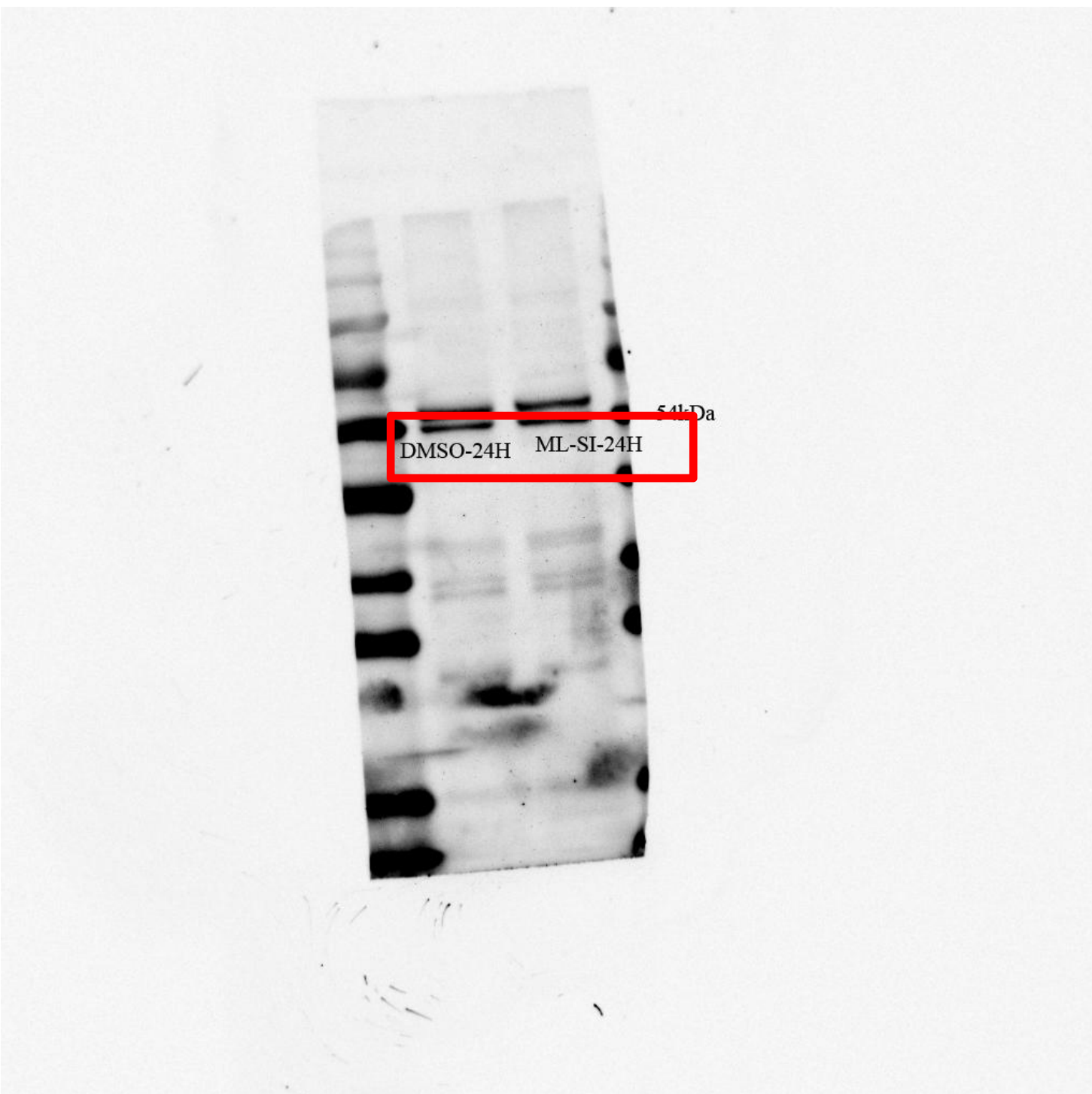

SMS

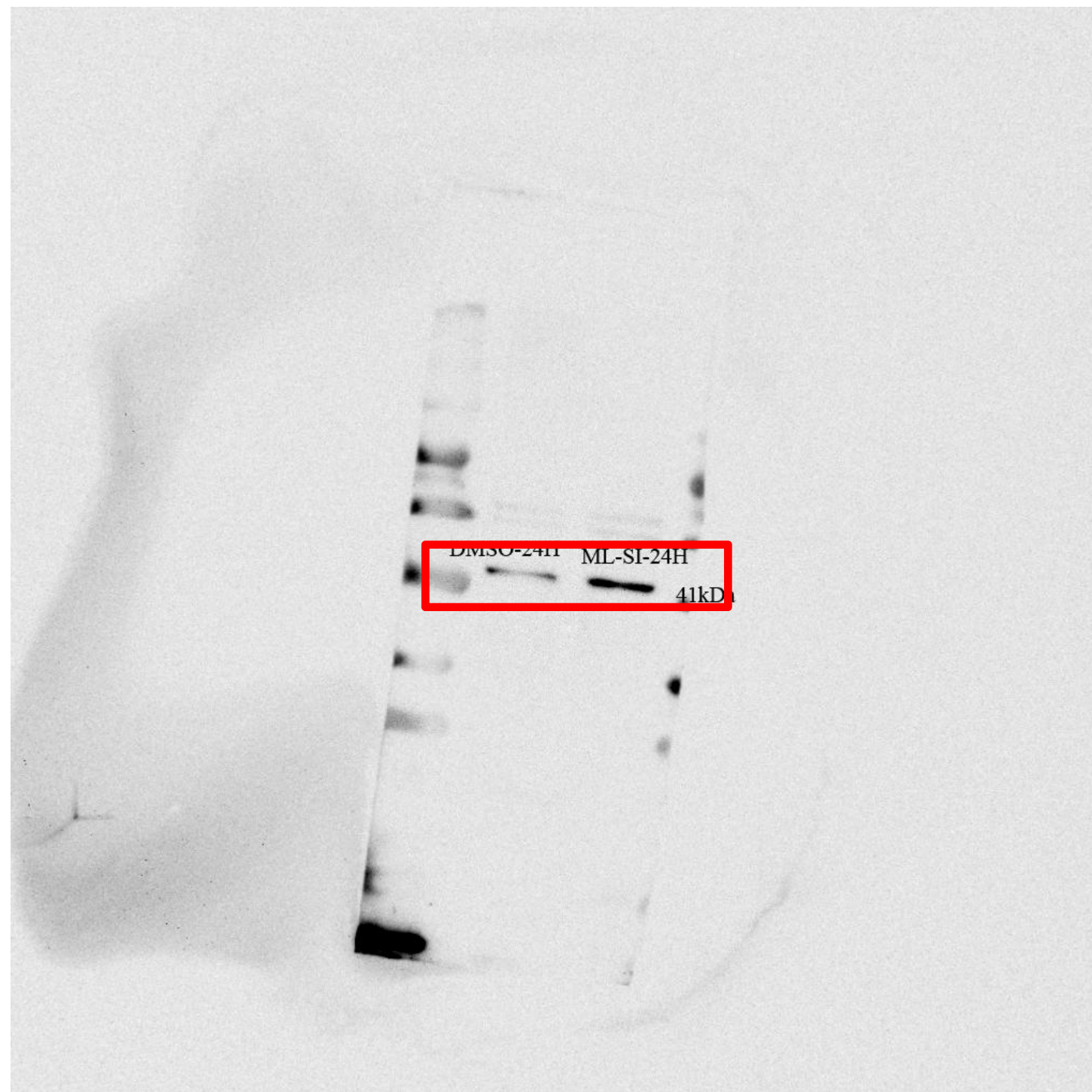

GAPDH

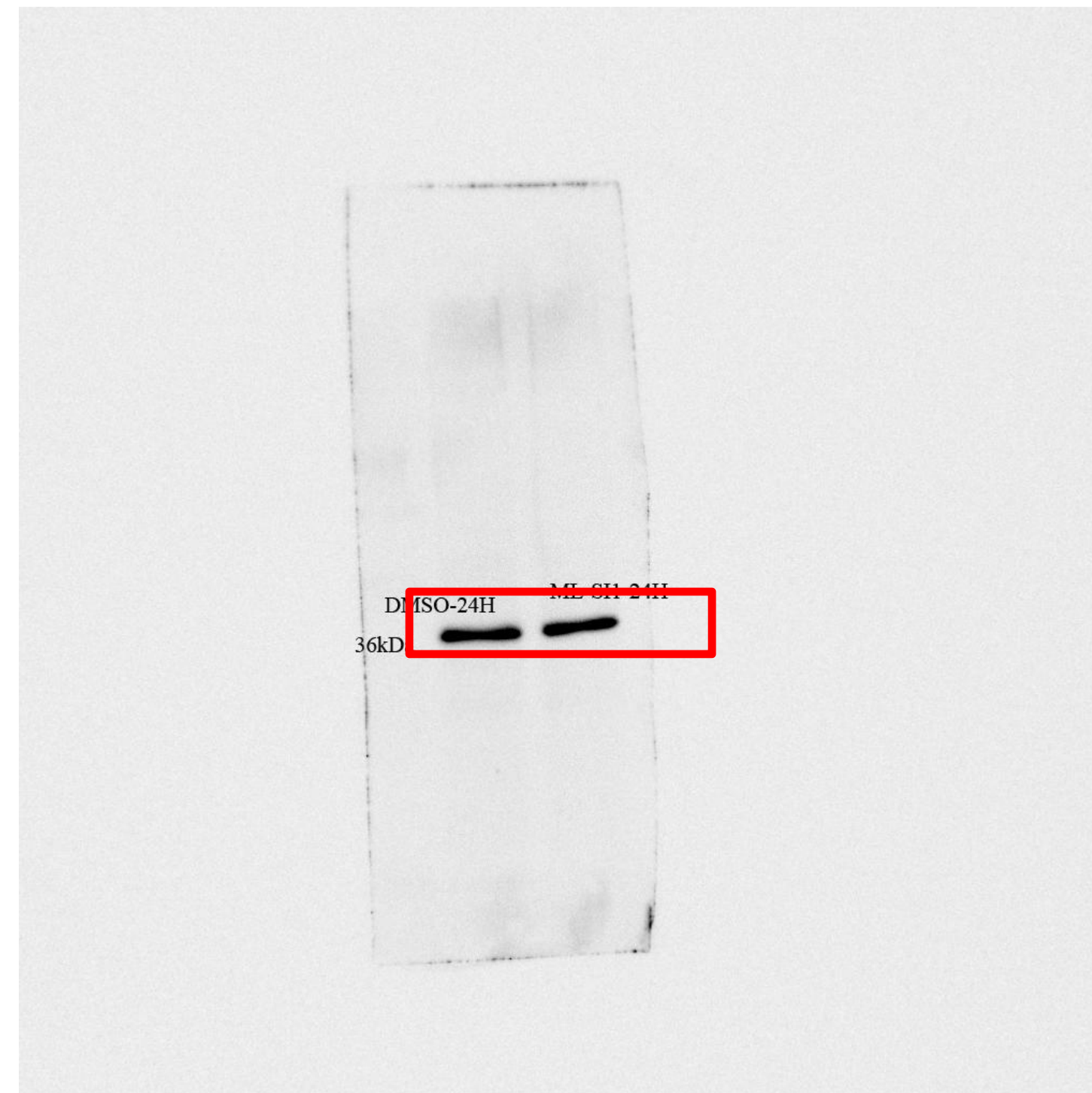

Figure 8E Group 2

ODC1

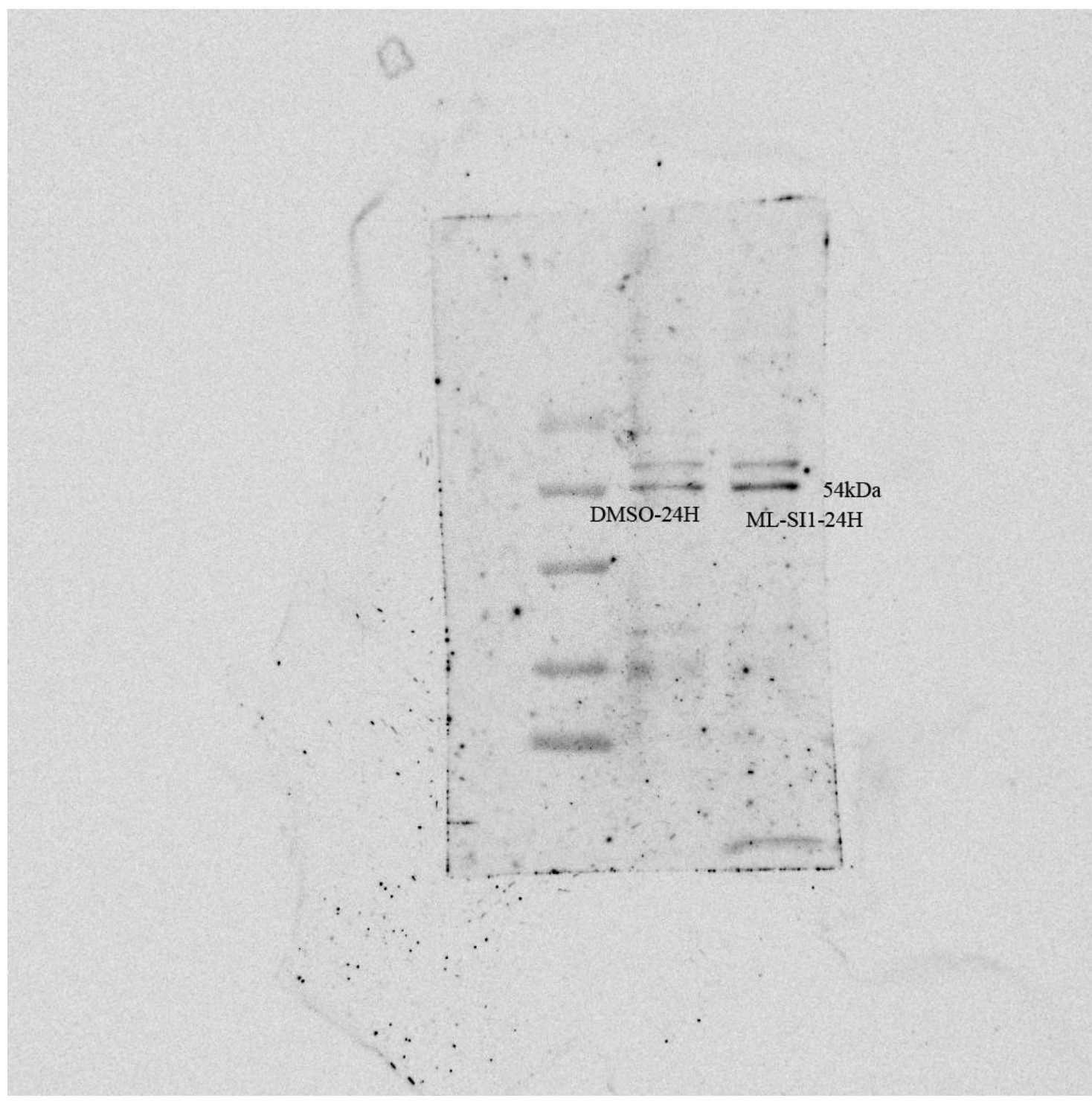

SMS

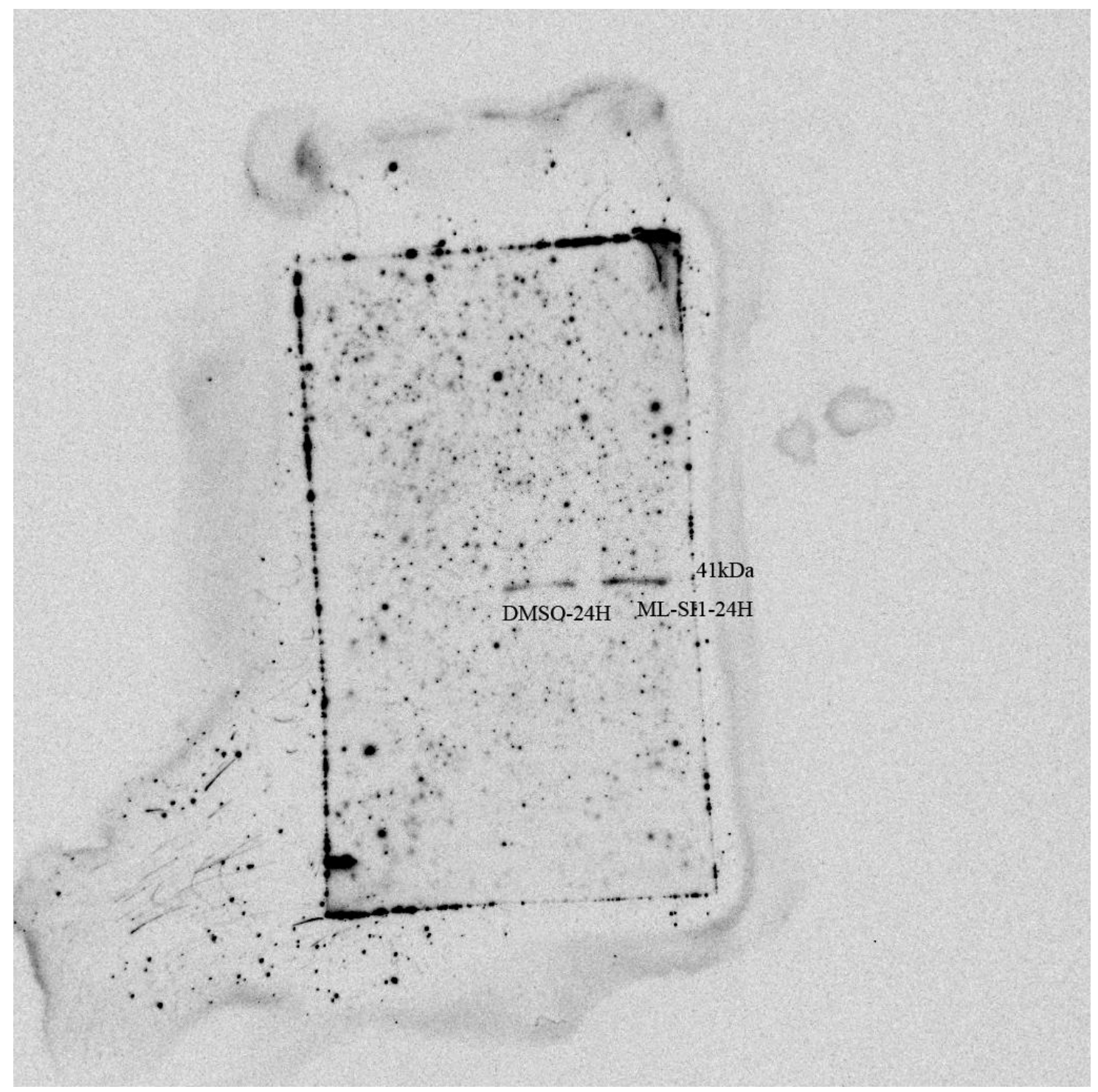

GAPDH

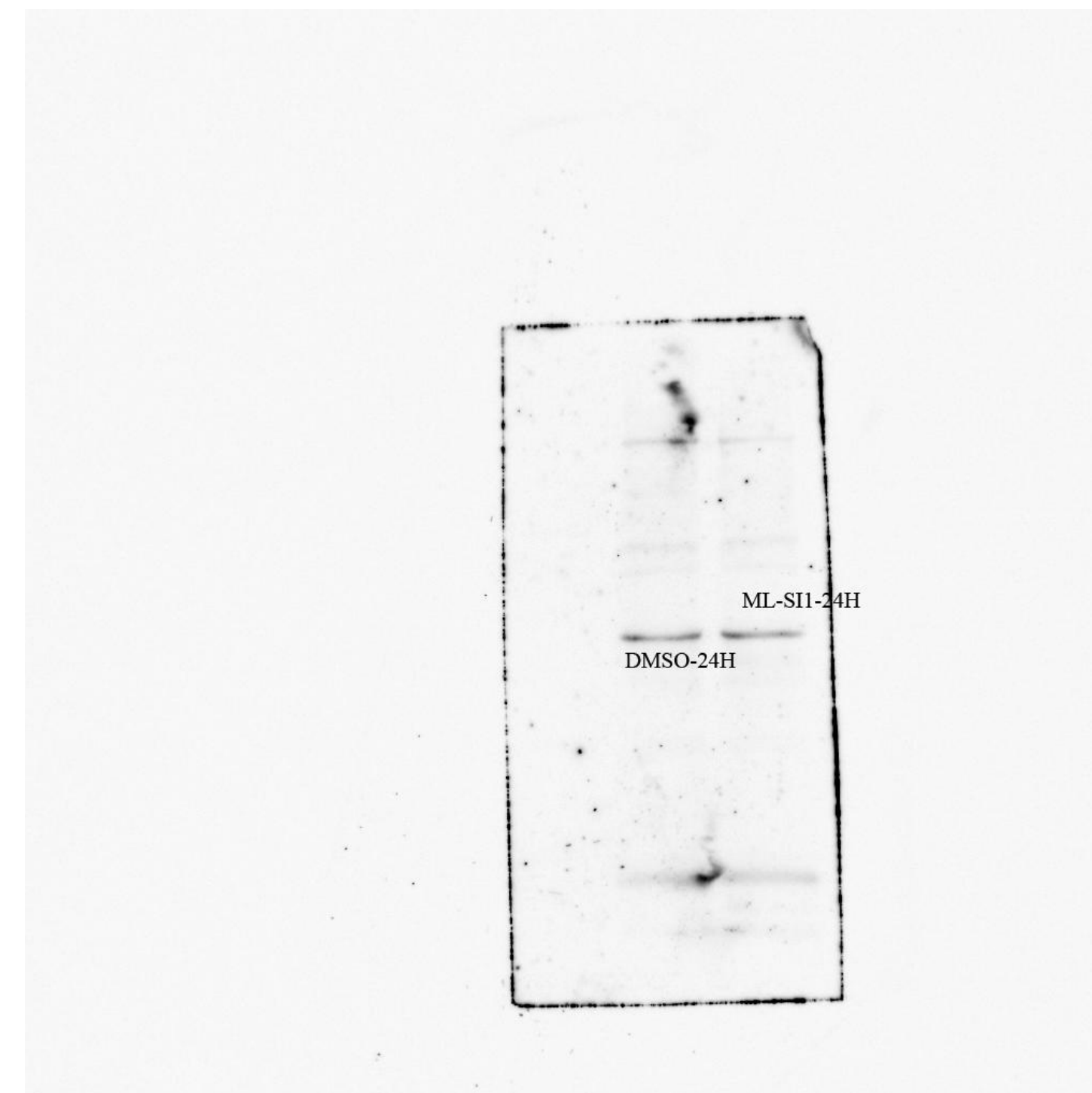

Figure 8E Group 3

ODC1

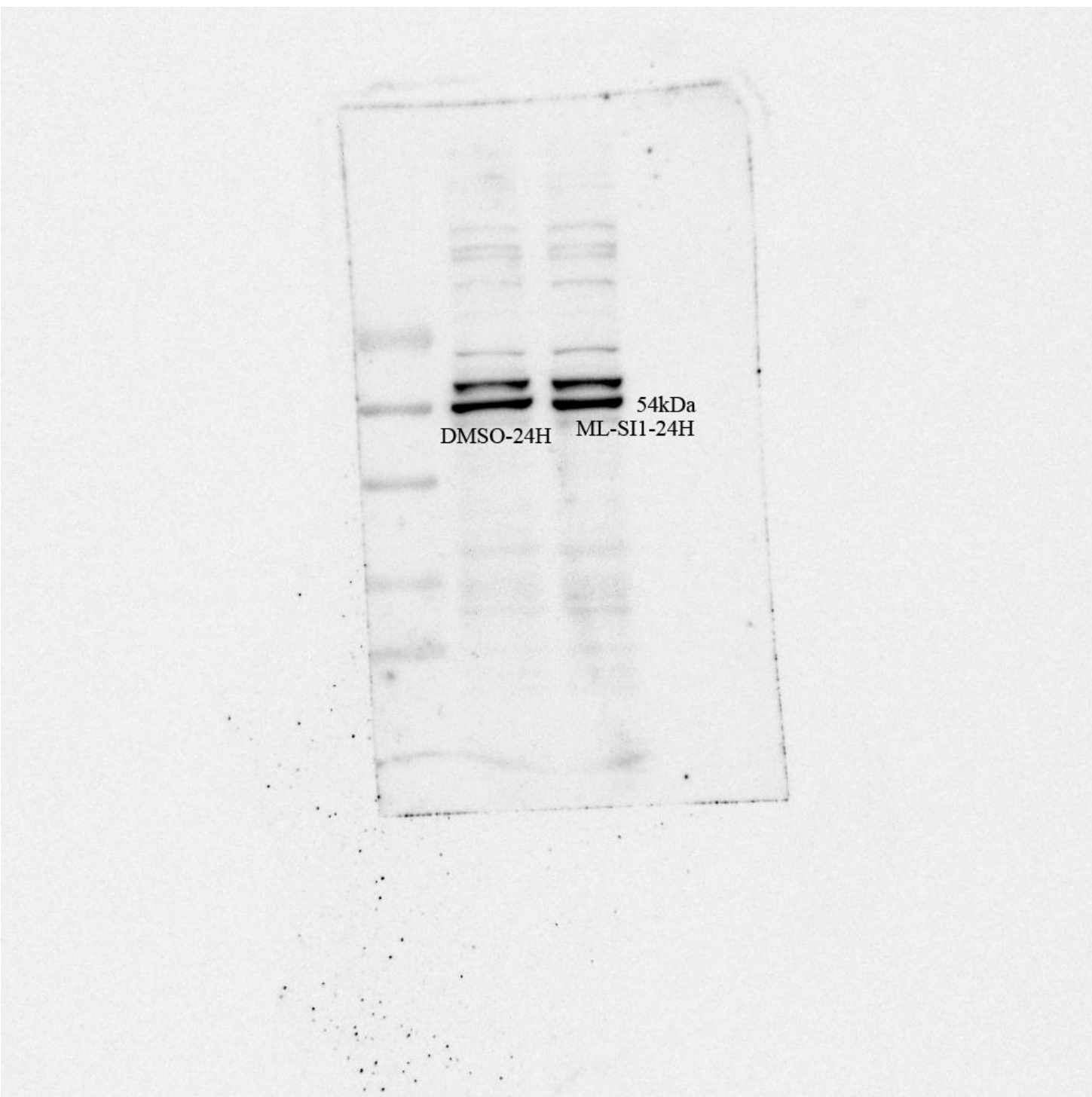

SMS

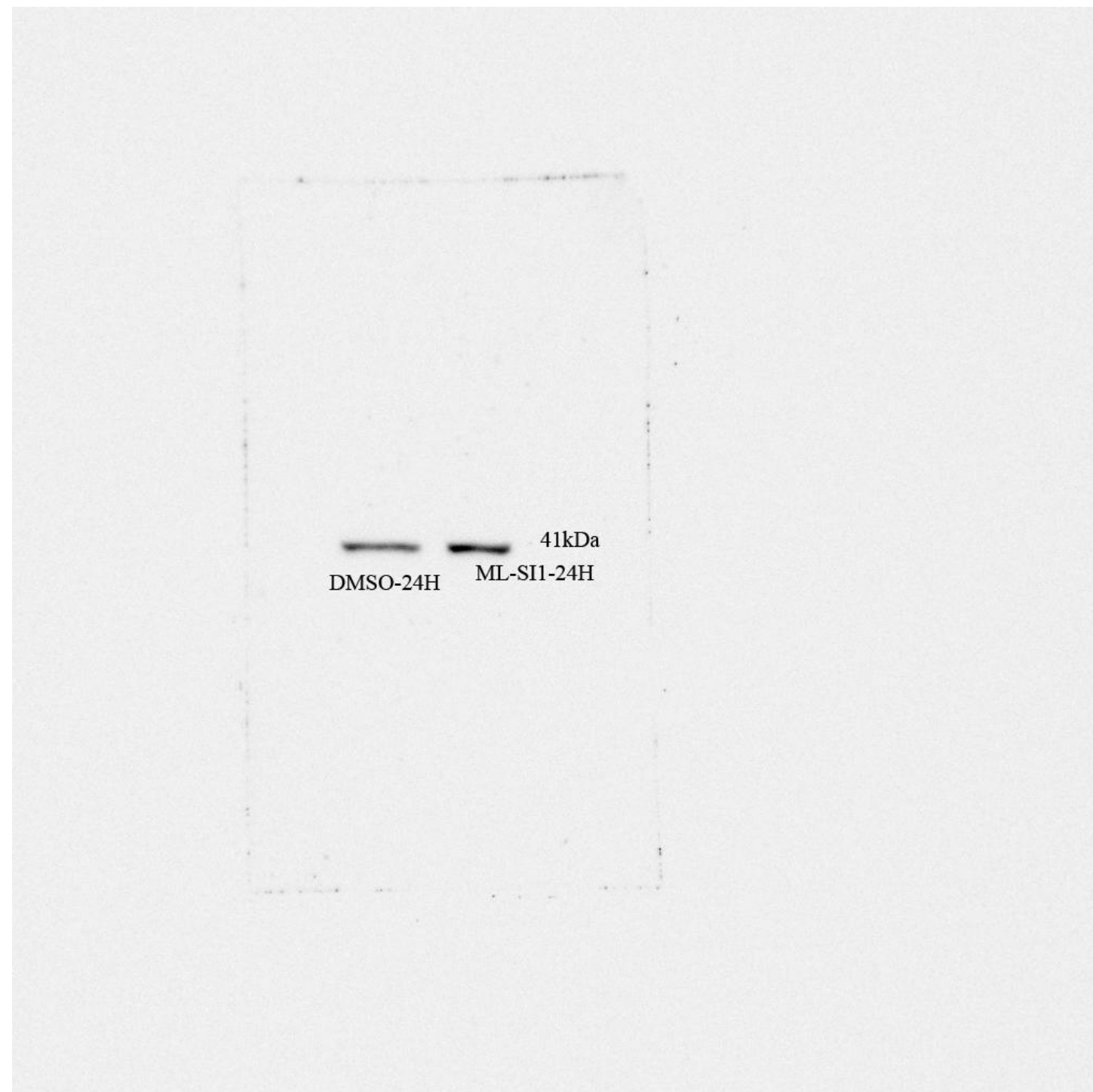

GAPDH

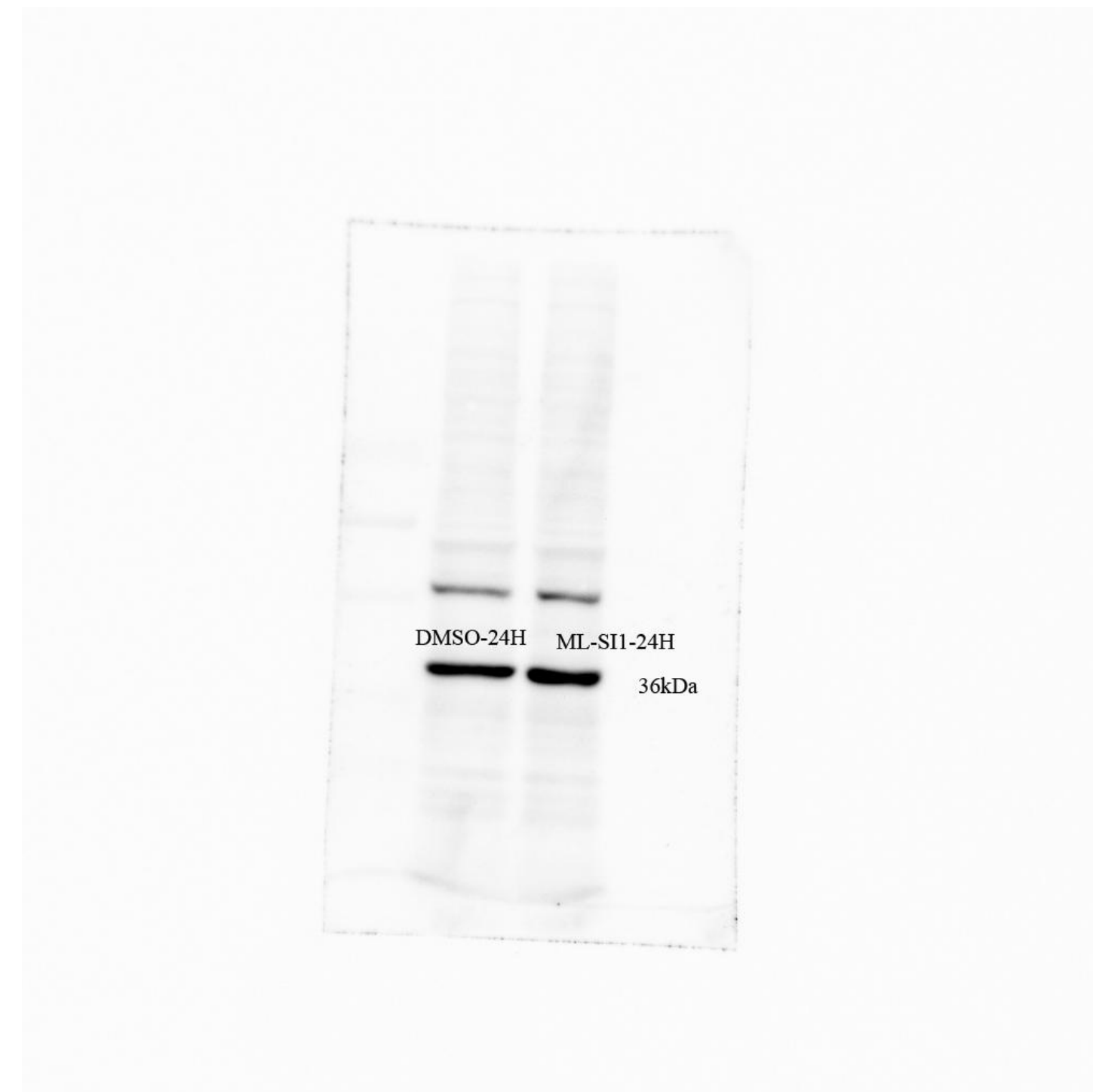

Figure 8F Group 1

ODC1

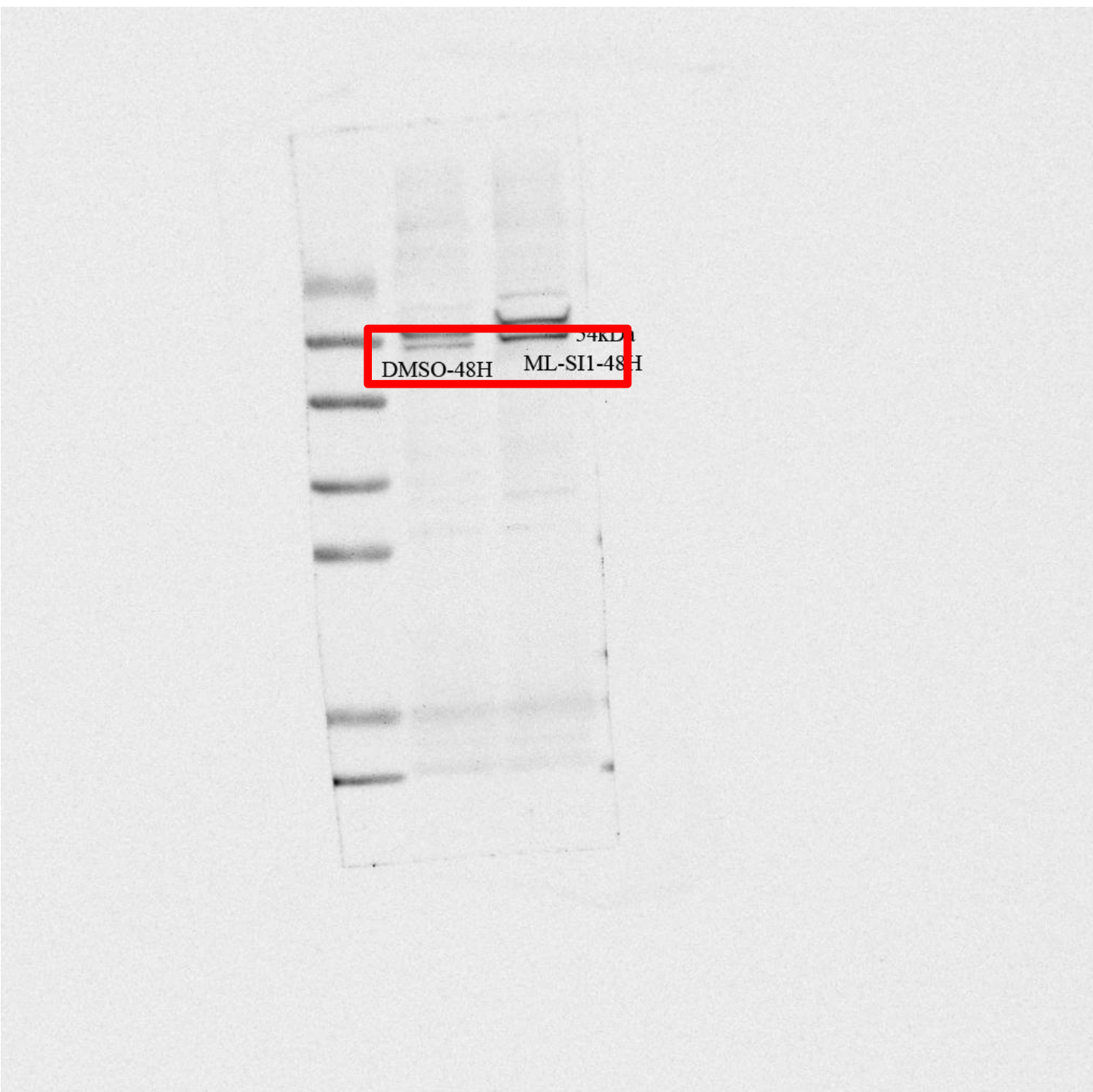

SMS

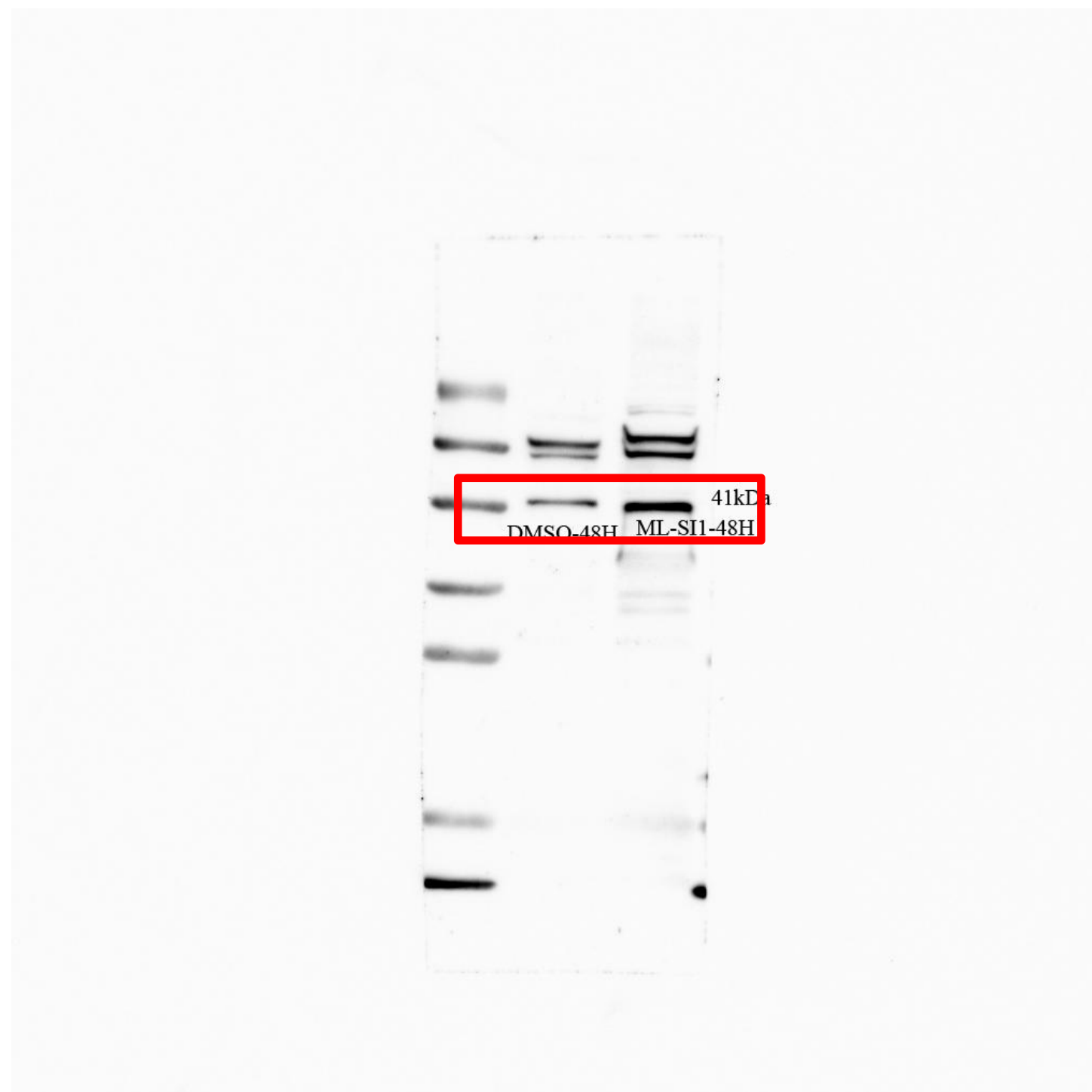

GAPDH

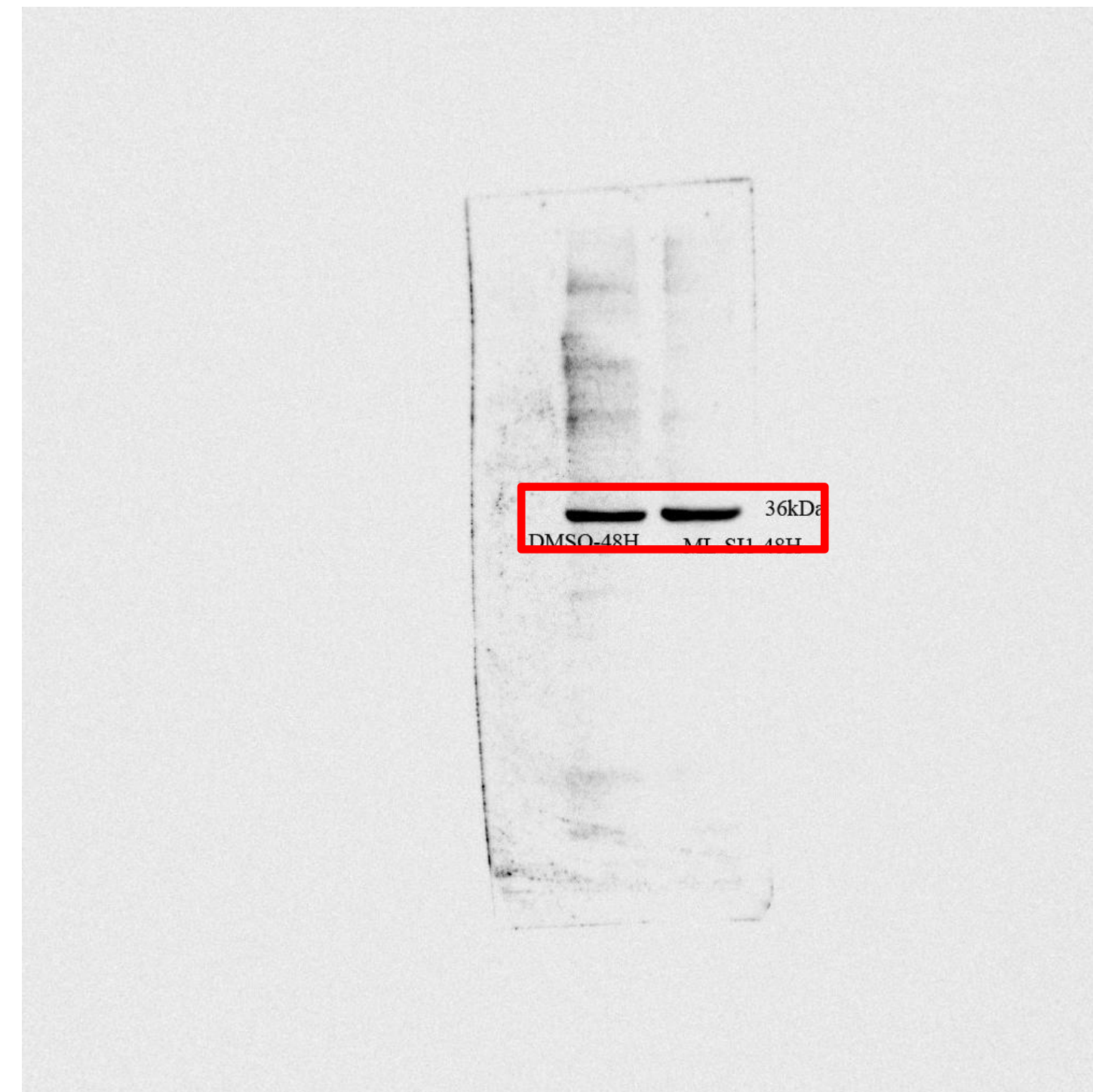

Figure 8F Group 2

ODC1

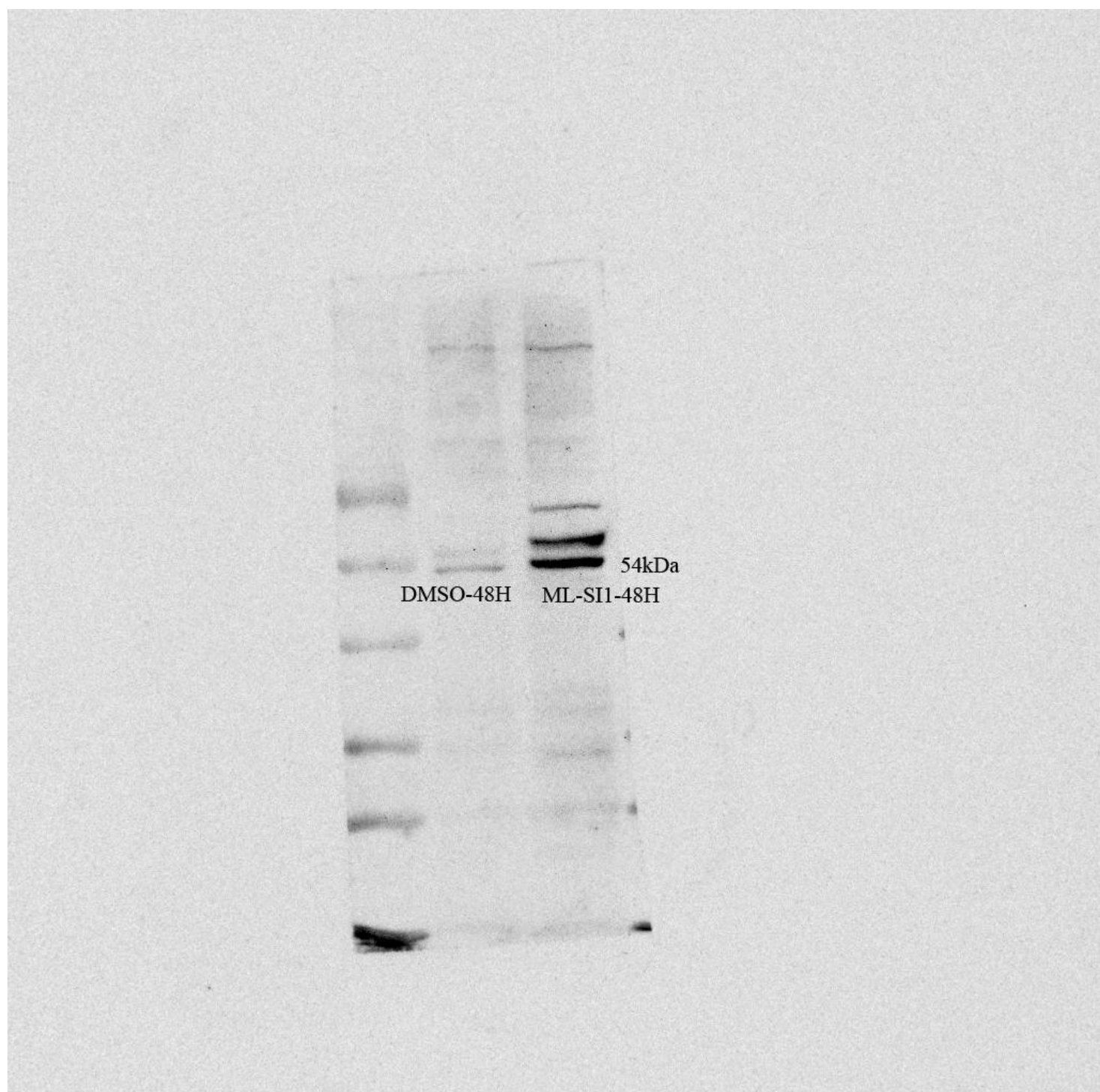

SMS

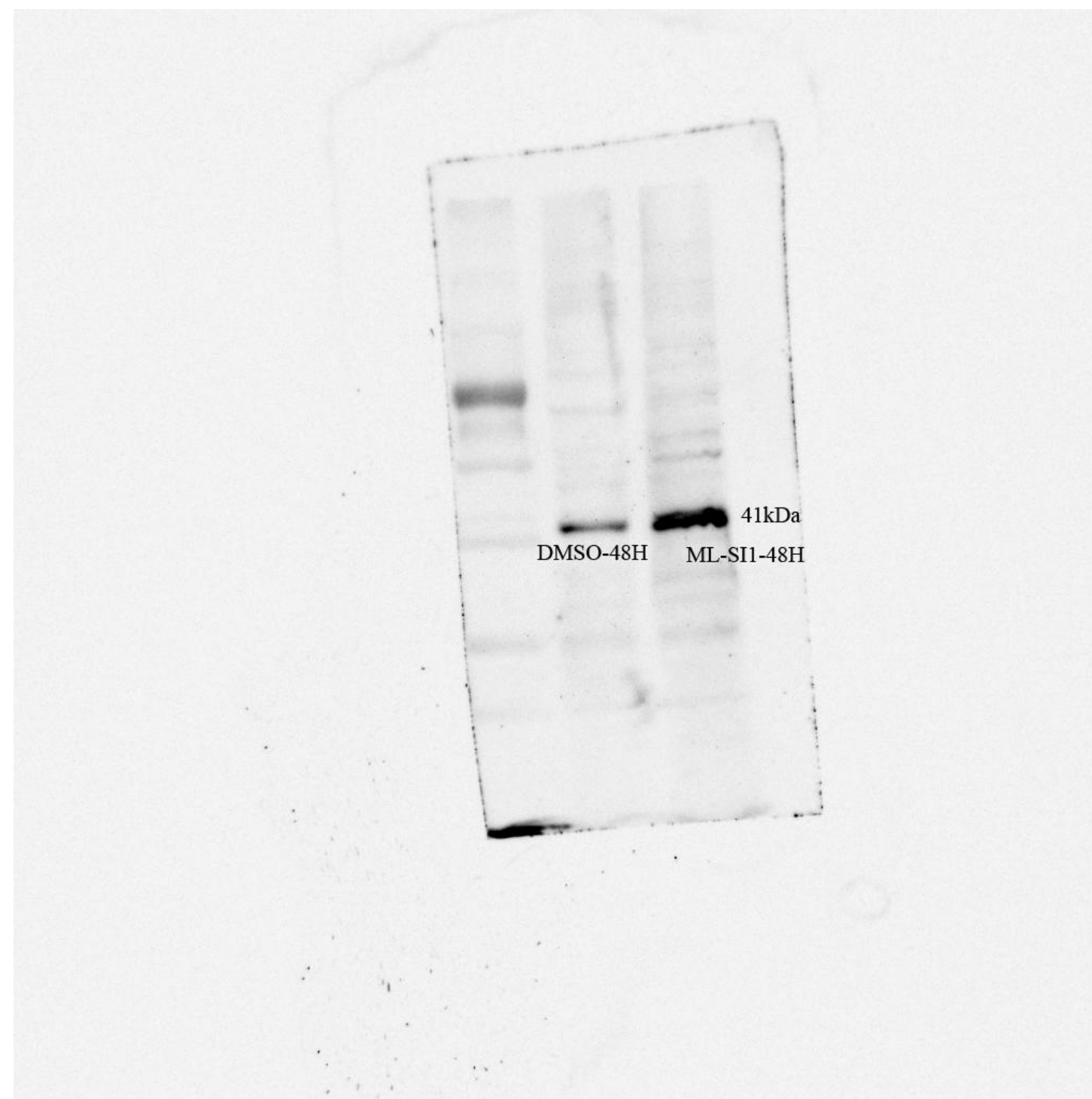

GAPDH

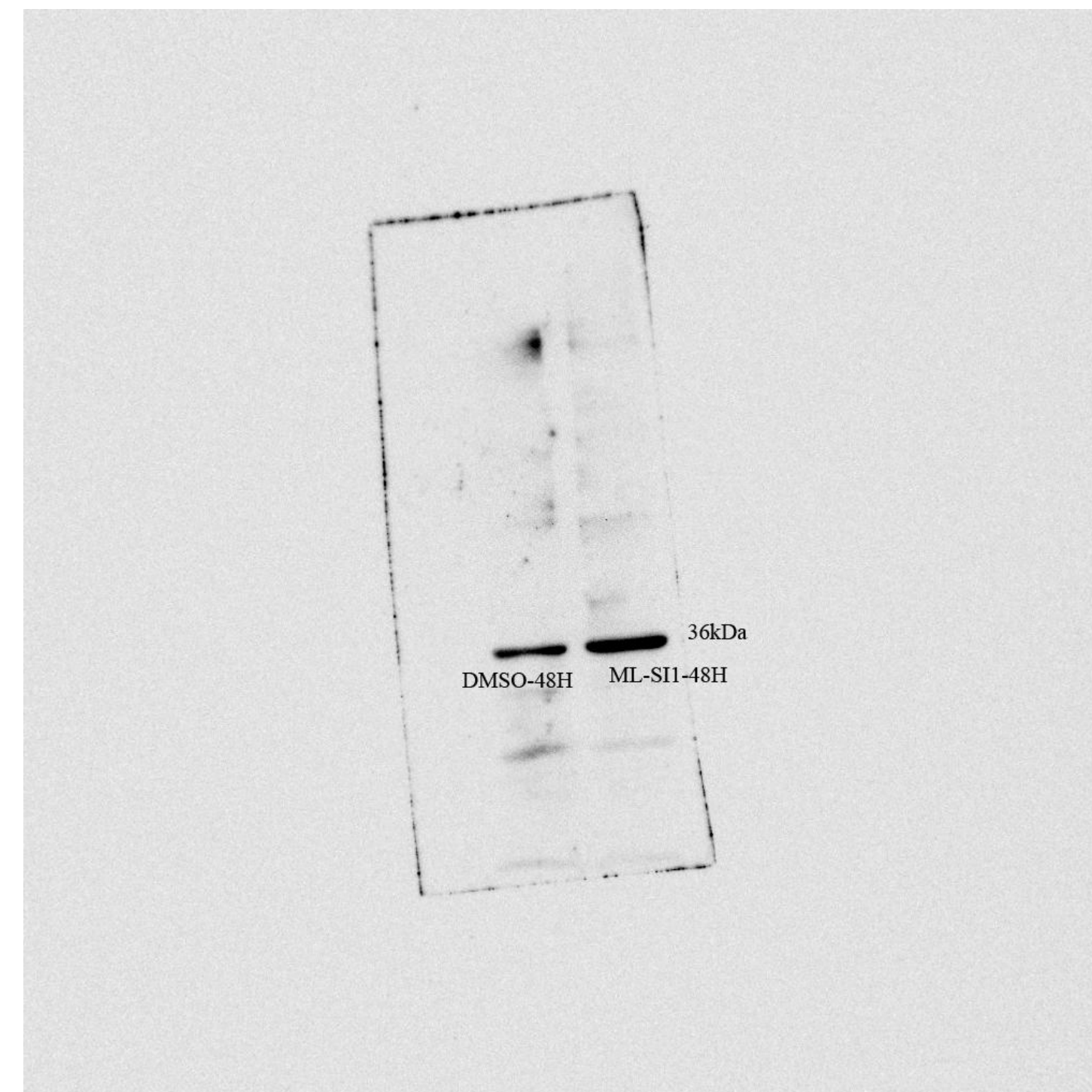

Figure 8F Group 3

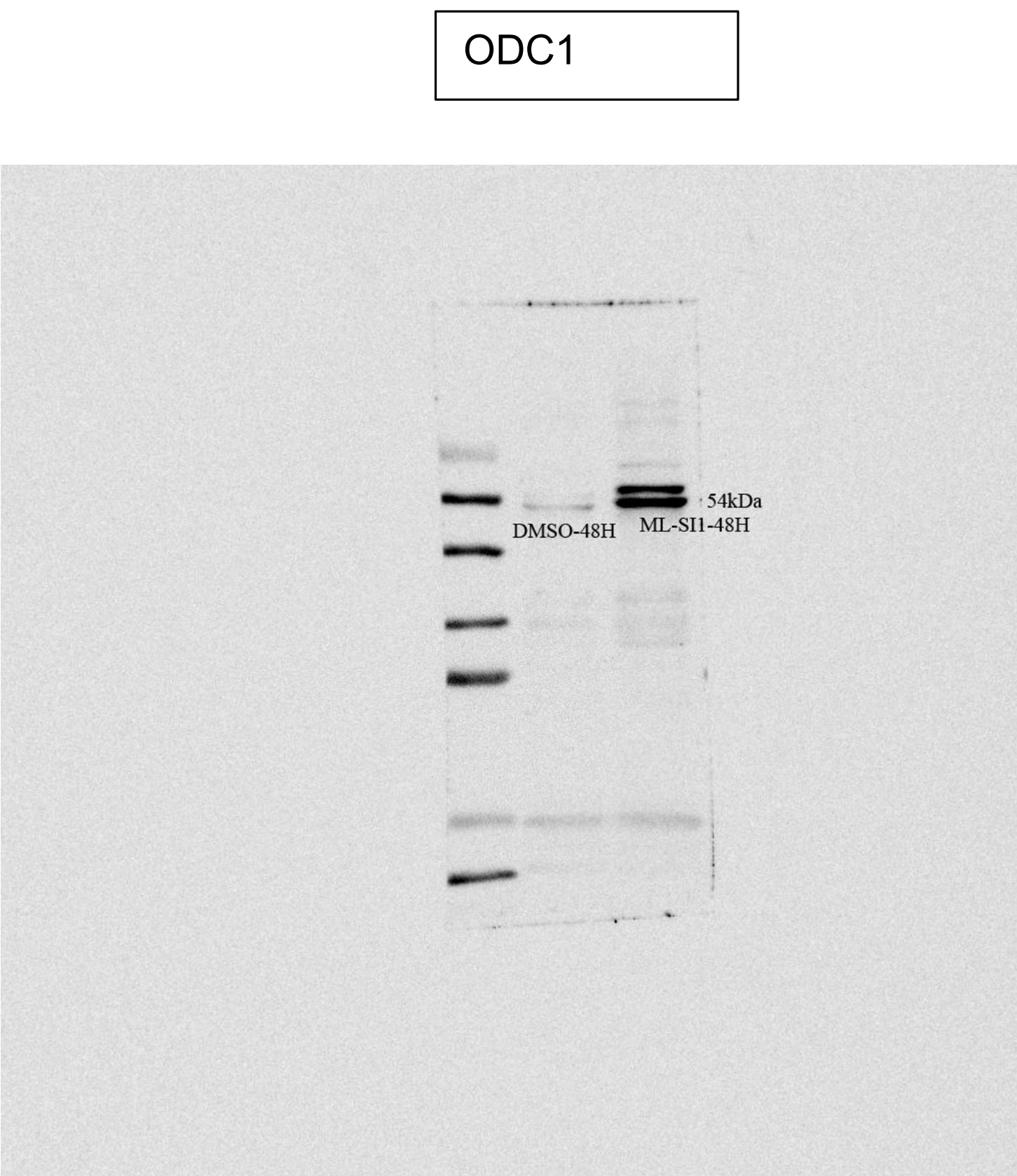

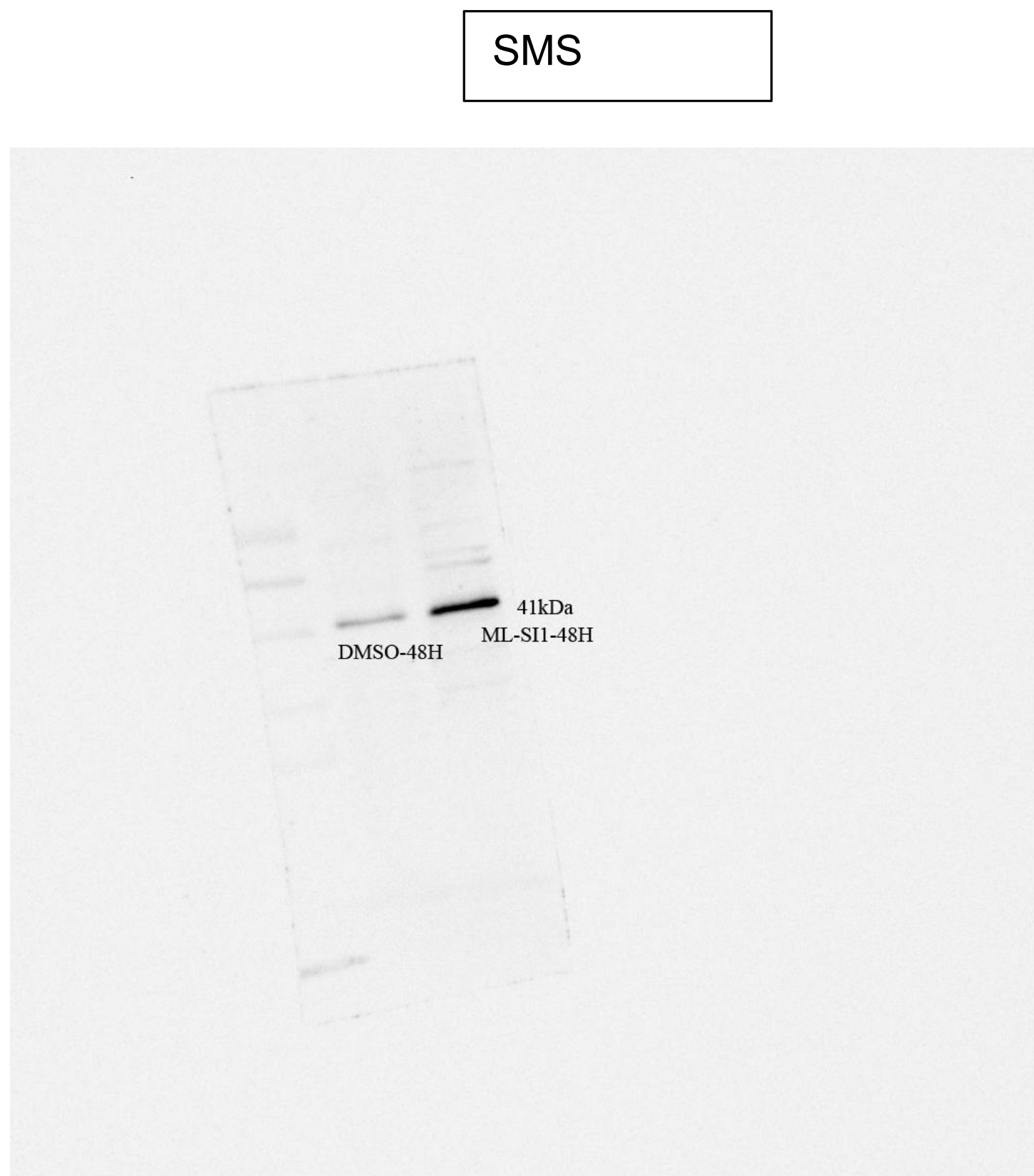

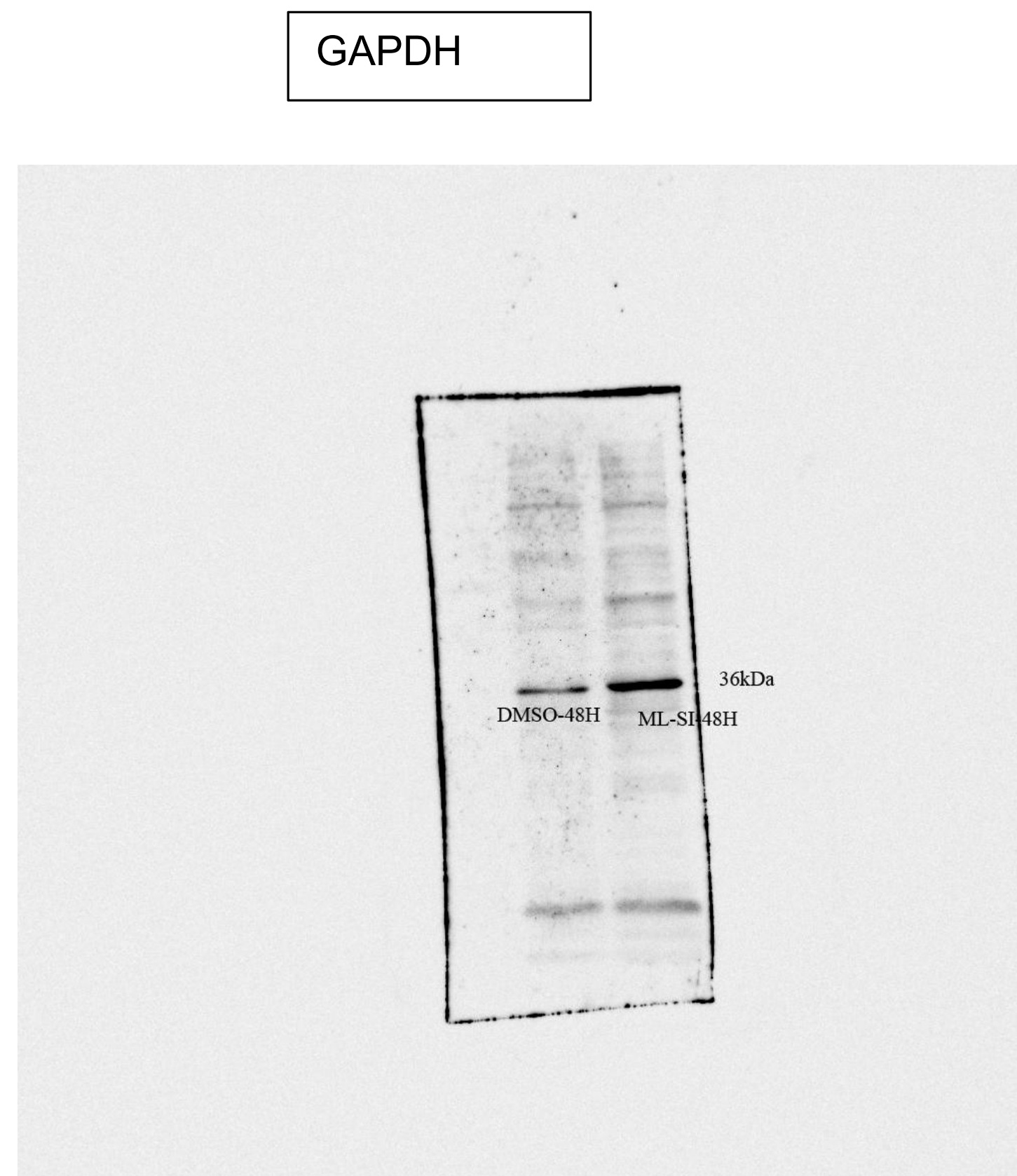

Supplementary Figure S3A Group 1

AR

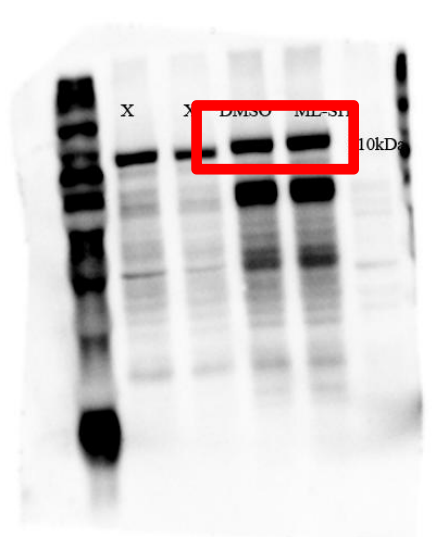

AR-V7

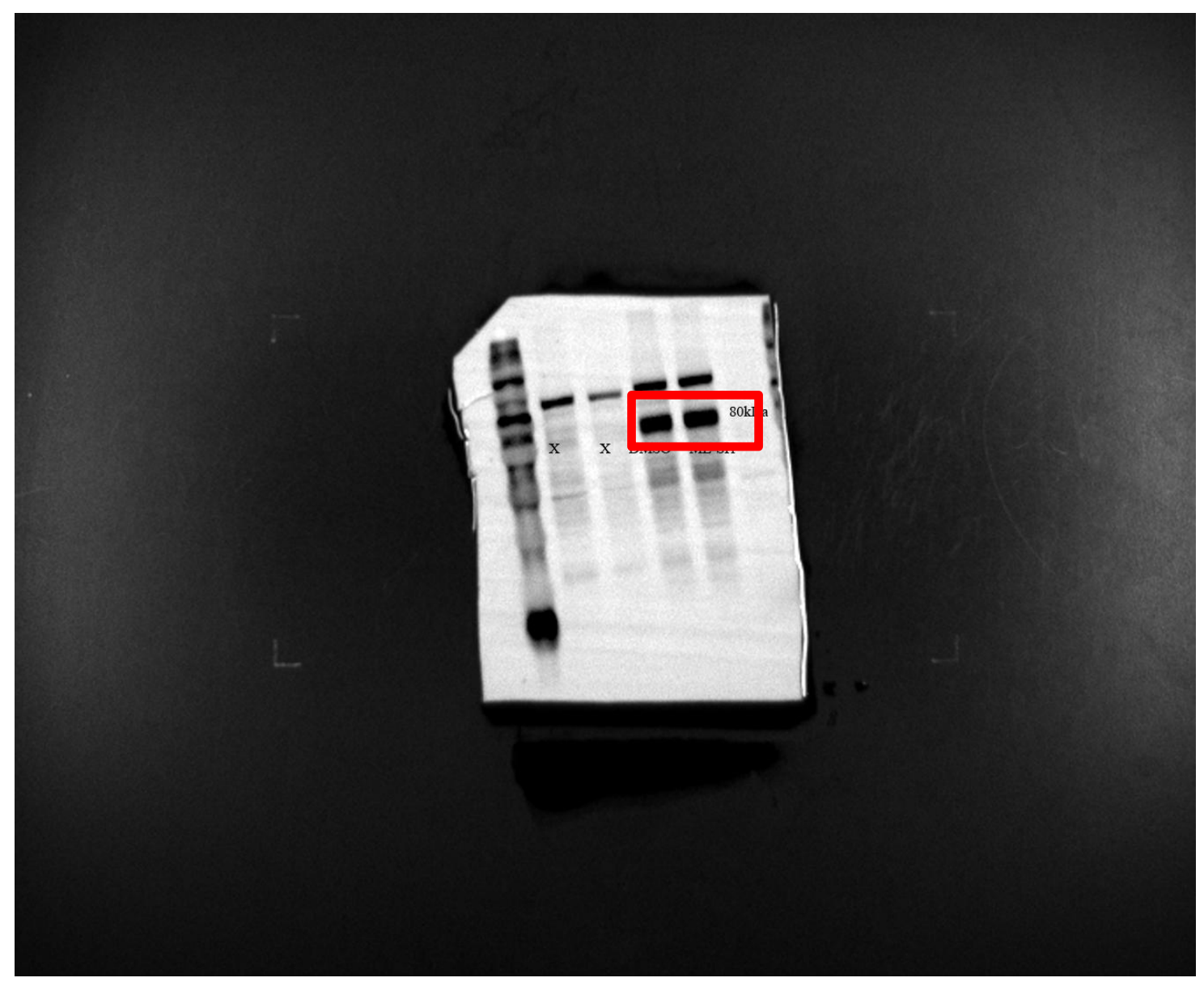

β -actin

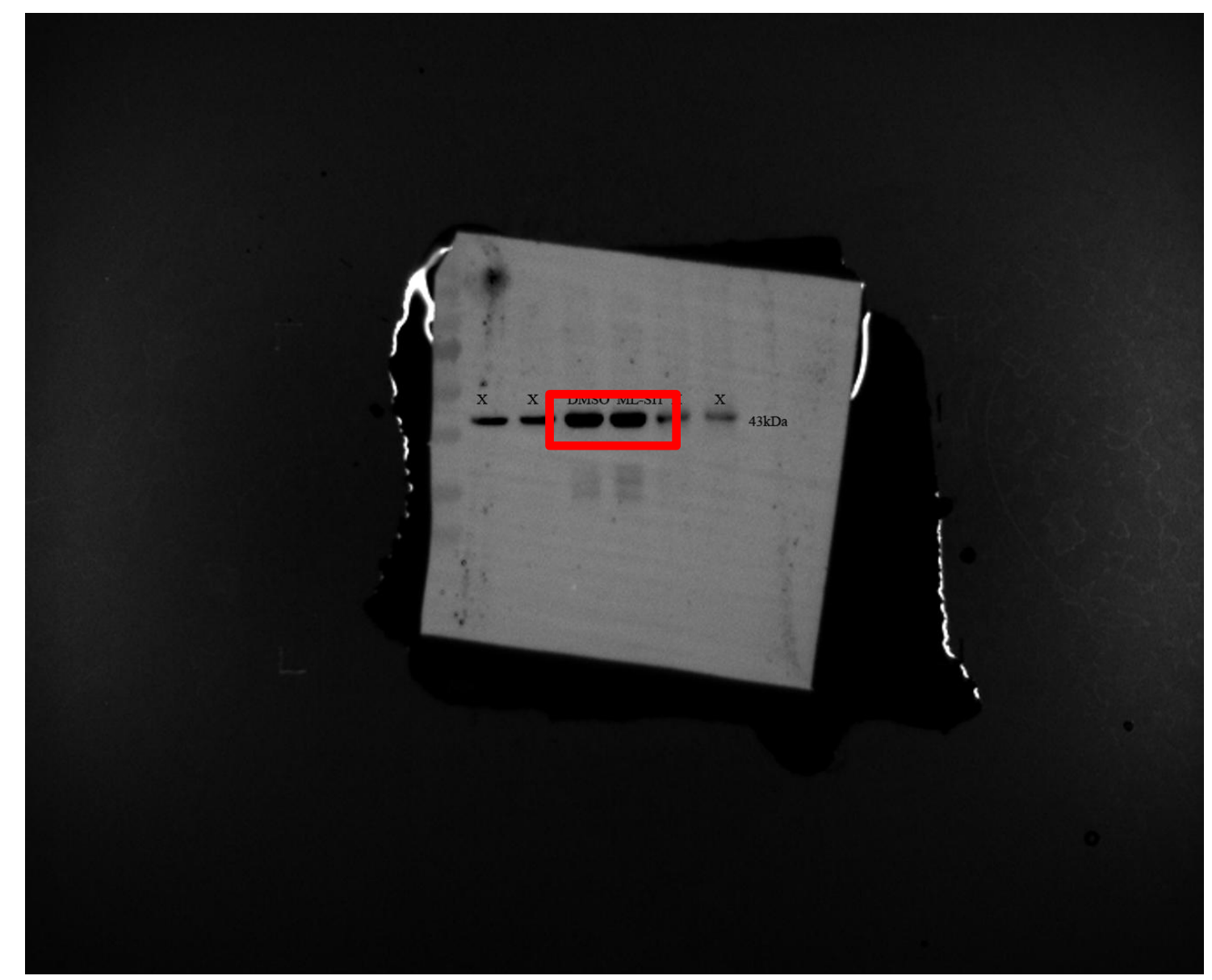

Supplementary Figure S3A   Group 2

AR

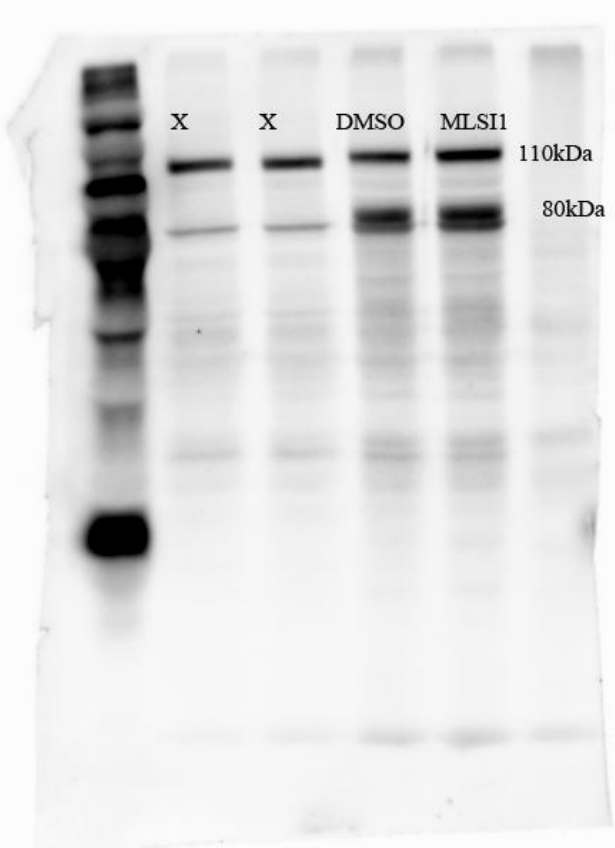

AR-V7

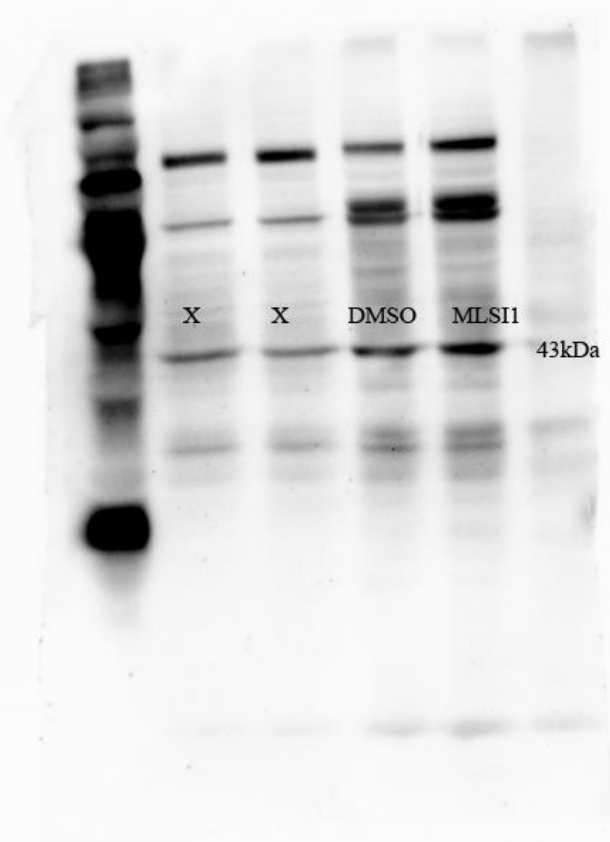

β -actin

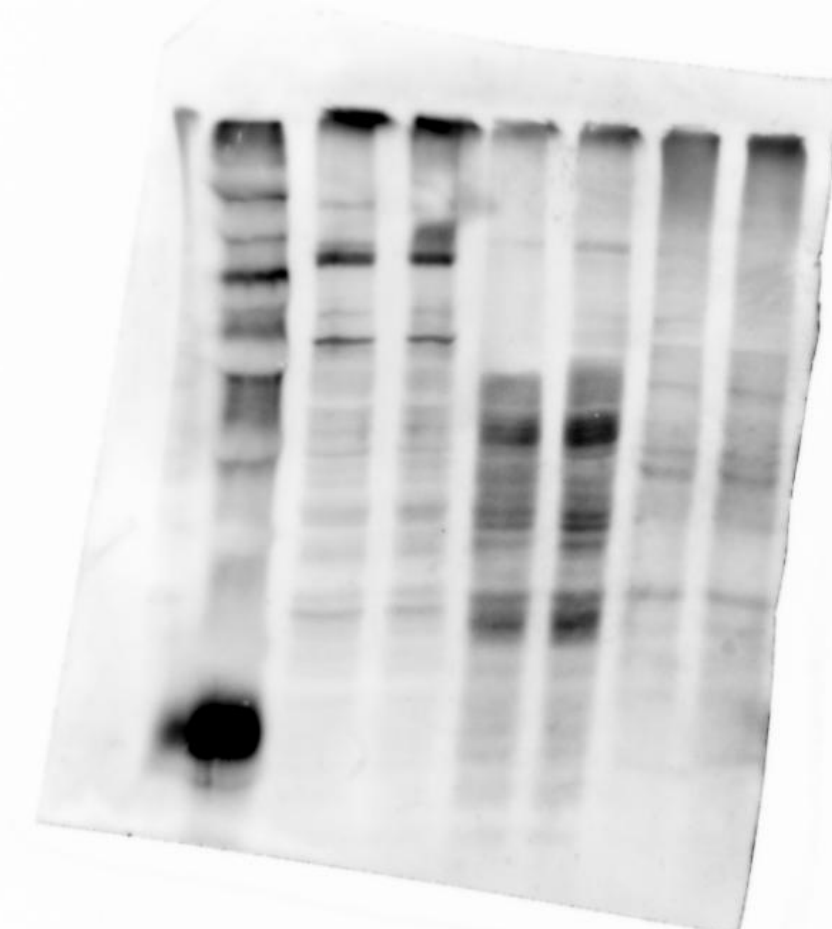

Supplementary Figure S3A Group 3

AR

AR-V7

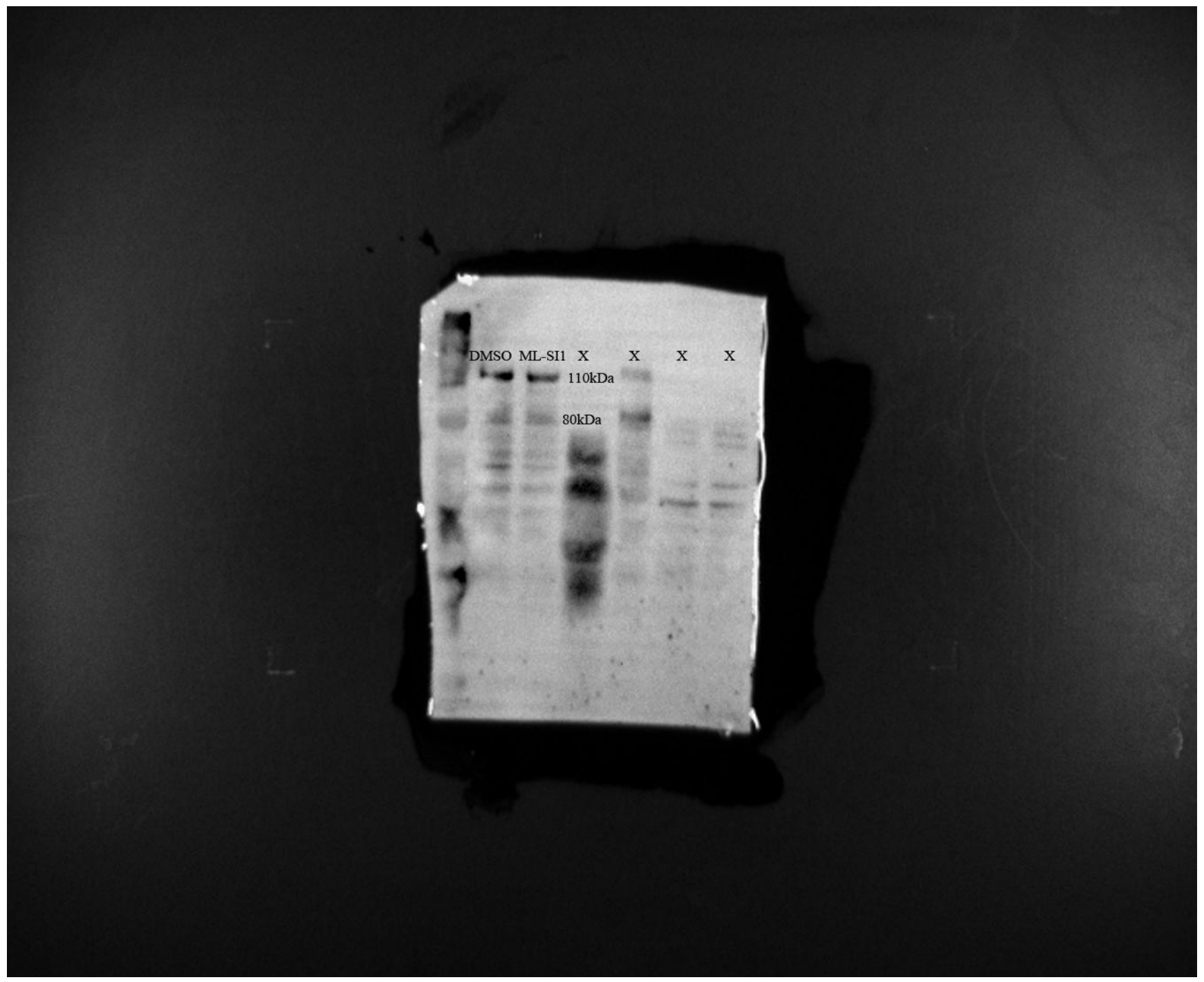

β -actin

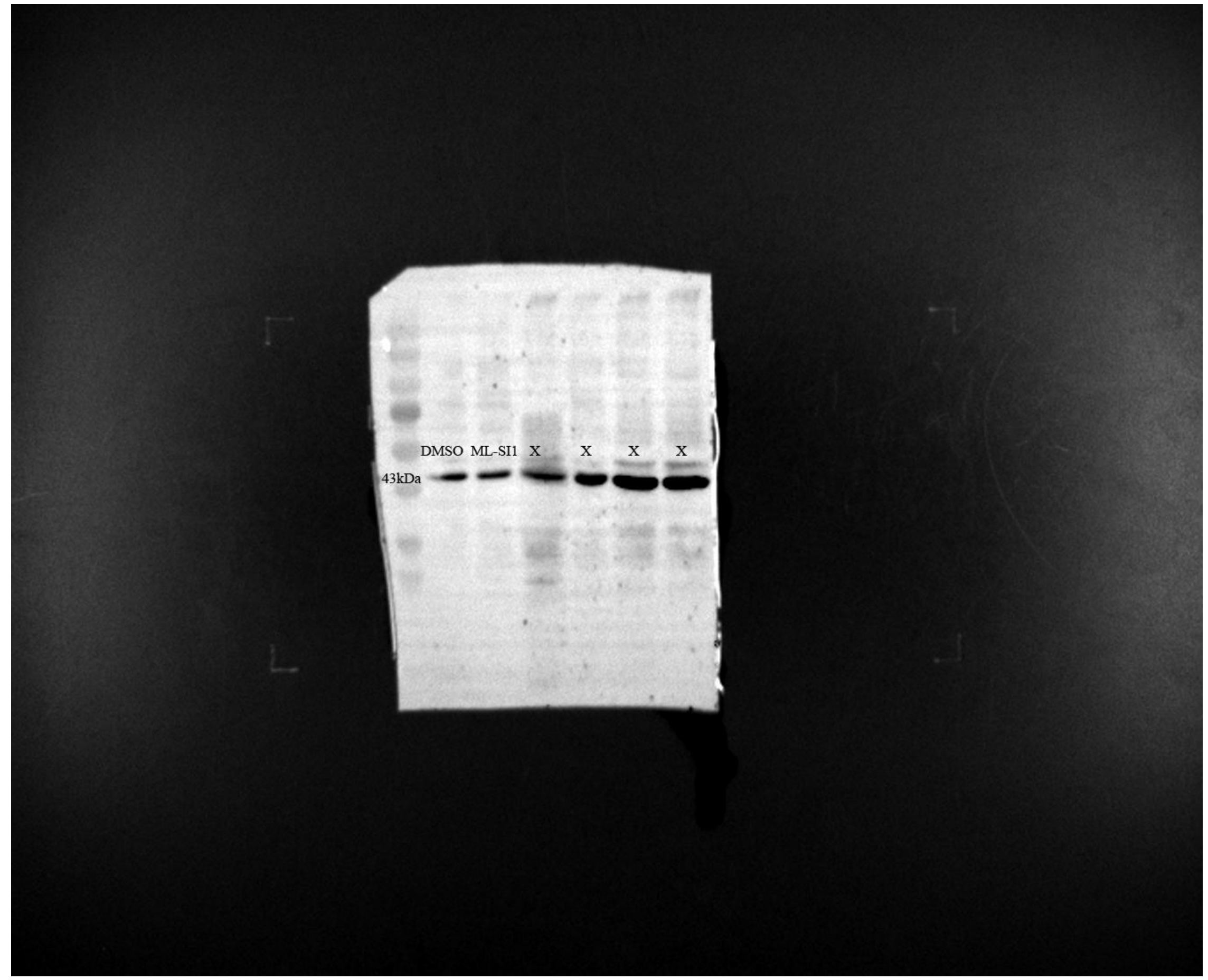

Supplement: Supplementary file 1 [file Supplementaryfile1.pdf]
