# Supplementary material for: TRPML1 inhibitor ML-SI1 suppresses prostate cancer through dual regulation of androgen receptor and polyamine metabolism
Source: Front Med (Lausanne). 2026 Sep 28;13:1959805. doi: 10.3389/fmed.2026.1959805 (PMC13654739; doi:10.3389/fmed.2026.1959805)
Supplement: Supplementary file 2 [file Datasheet1.docx]

Supplementary Material

# 1 Supplementary Figures

**Supplementary Figure S1.** TRPMLs distribution in various tissues and organs in the human body. **(A)** Schematic diagram of distribution of TRPMLs in the male body. RNA and protein expression of **(B)** TRPML1, **(C)** TRPML2, and **(D)** TRPML3, respectively.

**Supplementary Figure S2.** Effect of ML-SI1 on cell-cycle distribution in 22Rv1 cells. 22Rv1 cells were treated with DMSO or 15 μM ML-SI1 for 24 h, and the percentages of cells in the G0/G1, S, and G2/M phases were determined by flow cytometry. No significant differences were observed in cell-cycle distribution between the two groups. Data are presented as mean ± SEM. ns, not significant.

**Supplementary Figure S3.** Transcriptomic analysis of the effect of ML-SI1 on LNCaP cells. (A) Histogram, (B) Volcano plot, and (C) Heat map.

**Supplementary Figure S4.** Effect of ML-SI1 on AR and AR-V7 protein abundance in 22Rv1 cells. (A) Representative western blot images of AR and AR-V7 in 22Rv1 cells treated with DMSO or ML-SI1. (B) Densitometric analysis of AR and AR-V7 protein abundance shown in (A), normalized to β-actin. ns, not significant.
